# Functional oncogene signatures guide rationally designed combination therapies to synergistically induce breast cancer cell death

**DOI:** 10.18632/oncotarget.9147

**Published:** 2016-05-02

**Authors:** Stephen T. Guest, Zachary R. Kratche, Jonathan C. Irish, Robert C. Wilson, Ramsi Haddad, Joe W. Gray, Elizabeth Garrett-Mayer, Stephen P. Ethier

**Affiliations:** ^1^ Department of Pathology and Laboratory Medicine, Hollings Cancer Center, Medical University of South Carolina, Charleston, South Carolina, USA; ^2^ Department of Public Health Science, Medical University of South Carolina, Charleston, South Carolina, USA; ^3^ Department of Biomedical Engineering, Oregon Health and Sciences University, Portland, Oregon, USA; ^4^ Knight Cancer Institute, Oregon Health and Sciences University, Portland, Oregon, USA

**Keywords:** breast cancer, functional genomics, BCL2L1, FGFR, AKT

## Abstract

A critical first step in the personalized approach to cancer treatment is the identification of activated oncogenes that drive each tumor. The Identification of driver oncogenes on a patient-by-patient basis is complicated by the complexity of the cancer genome and the fact that a particular genetic alteration may serve as a driver event only in a subset of tumors that harbor it. In this study, we set out to identify the complete set of functional oncogenes in a small panel of breast cancer cell lines. The cell lines in this panel were chosen because they each contain a known receptor tyrosine kinase (RTK) oncogene. To identify additional drivers, we integrated functional genetic screens with copy number and mutation analysis, and cancer genome knowledge databases. The resulting functional oncogene signatures were able to predict responsiveness of cell lines to targeted inhibitors. However, as single agents, these drugs had little effect on clonogenic potential. By contrast, treatment with drug combinations that targeted multiple oncogenes in the signatures, even at very low doses, resulted in the induction of apoptosis and striking synergistic effects on clonogenicity. In particular, targeting a driver oncogene that mediates AKT phosphorylation in combination with targeting the anti-apoptotic BCL2L1 protein had profound effects on cell viability. Importantly, because the synergistic induction of cell death was achieved using low levels of each individual drug, it suggests that a therapeutic strategy based on this approach could avoid the toxicities that have been associated with the combined use of multiple-targeted agents.

## INTRODUCTION

Cancer is a genomic disease that results from the progressive acquisition of dominant genomic alterations in driving oncogenes and recessive mutations in tumor suppressor genes. Laboratory and clinical investigations have demonstrated that human cancer cells become dependent on the activity of driver oncogenes for the expression of transformed phenotypes and for their survival. This idea, known as oncogene addiction [1, 2], has become a cornerstone for the development of targeted cancer therapeutics because it results in cells that are dependent on the oncogene for survival, and increases the sensitivity of the addicted cells to oncogene-targeted drugs which provides the therapeutic index needed for cancer cell specific effects on viability [2]. There is ample evidence in support of this hypothesis from laboratory studies, but the most compelling evidence comes from clinical studies involving targeted agents such as imatinib [3–6], trastuzumab [7–9], erlotinib [10], and vemurafinib [11] which have yielded dramatic clinical responses when the cognate driving oncogene is present in the treated cancer cells. However, it has also been repeatedly observed that clinical responses to these targeted agents, even when they are dramatic, are transient and followed by recurrences of the disease. It is increasingly clear that multiple driver oncogenes become activated during the evolution of most solid tumors, resulting in cancer cells that harbor complex oncogene signatures, and we hypothesize that these complex oncogene signatures help to explain the transient response of cancer cells to attack on a single oncogene.

In this study, we set out to identify the complete set of driver oncogenes that are functioning in a small panel of breast cancer cell lines. We refer to this complete set of oncogenes as the functional oncogene signature of the cell line. In order to determine functional oncogene signatures for each cell line, we combined results of genome-wide functional genetic screens with copy number analysis, exome sequencing data, and cancer knowledge databases. Elucidation of functional oncogene signatures allowed us to identify novel driver oncogenes in each cell line, and accurately predicted the responsiveness and sensitivity of the cell lines to targeted drugs. The oncogene signatures also allowed us to identify drug combinations that resulted in striking, synergistic effects on breast cancer cell viability. Our results specifically support a therapeutic strategy targeting a driver oncogene that mediates AKT phosphorylation in combination with targeting the anti-apoptotic BCL2L1 protein when it is part of the functional oncogene signature. The synergy observed using this approach suggests that an effective response can be achieved using low levels of each individual drug and thereby avoid toxicities that have hampered the use of combination therapies in the clinic.

## RESULTS

### Elucidation of functional oncogene signatures in a small panel of breast cancer cell lines

A primary goal of the experiments reported here was to elucidate complete functional oncogene signatures for a small panel of SUM breast cancer cell lines [12], each of which is known to harbor a driving RTK oncogene activated by amplification (SUM-52/FGFR2, SUM-185/FGFR3, SUM-225/HER2, SUM-190/HER2) [13, 14]. In order to elucidate the functional oncogene signature for each of these cell lines, we first determined gene copy number status in each cell line because gene amplification is a common mechanism of oncogene activation. Array comparative genomic hybridization analysis revealed that each cell line harbors hundreds of genes that are copy number amplified (Supplementary Tables 1–4). This large number of amplified genes is a common feature of breast cancers [15]; however, only a small number of the amplified genes are likely to be functioning as activated driver oncogenes, with the remaining alterations being likely passenger genes.

To determine which of the amplified genes in each cell line play a functional role in driving cell growth and viability, and are therefore likely activated driver oncogenes, we performed a genome-scale shRNA growth and viability screen on each cell line. For each screen, cells were transduced with a library of 82,000 lentiviral vectors expressing shRNAs targeting 15,377 cellular genes, with a minimum of four shRNAs per gene. Cells were harvested at day 3 after selection and again after approximately 5–7 population doublings. The abundance of shRNAs at both time points was determined by next-generation sequencing of PCR-amplified shRNA-associated barcodes. Fold depletion values were used to calculate a gene level score for each gene and gene level scores were then used to identify significantly depleted genes (See Methods and [16, 17]) (Supplementary Tables 5–8).

Integrating the data from the shRNA screens with the gene copy number data allowed us to identify the copy number amplified genes that were also essential for growth and viability of the cells. Tables 1–4 show the results of this analysis for each of the four cell lines. This analysis dramatically reduced the number of candidate oncogenes in each of the cell lines from hundreds, to less than 50. Interestingly, we observed that in all four cell lines, the majority of amplicons harbored multiple genes that were hits in the screen. This result is consistent with the hypothesis that amplicons are selected for during cancer evolution because they harbor multiple genes important to the growth and survival of the cancer cells.

**Table 1:** SUM-52 copy number amplified genes that are hits in the shRNA screen

Gene symbol	Locus	Gene ranker score^1^	GISTIC^2^
CDK6	7q21	7	Yes
TFR2	7q22	1	Yes
CPSF4	7q22.2	1	Yes
BET1	7q21.1	0.5	Yes
KAT6A	8p11	3.5	Yes
STAR	8p11.2	1	Yes
CPSF1	8q24.2	0	Yes
MAF1	8q24.3	0	Yes
PUF60	8q24.3	0	Yes
DUSP26	8p12	3.5	No
NRG1	8p12	0	No
FGFR2	10q26	6.25	Yes
RPS3	11q13.3	1	Yes
CAPN5	11q14	0	Yes
TBK1	12q14.1	2.5	Yes
IL22	12q15	0	Yes
KRR1	12q21.2	1	No
SLTM	15q22.1	0	No
DDX5	17q21	1.5	Yes
CD79B	17q23	1	Yes
TLK2	17q23.2	1	Yes
TBX2	17q23.2	0.5	Yes
PTPRH	19q13.4	2	Yes
NDUFB7	19q13.12	1	Yes
TNNT1	19q13.4	0	Yes
PLAUR	19q13	1.75	No
PRPF31	19q13	1.5	No
RELB	19q13.3	1.5	No
RUVBL2	19q13.3	1.5	No
SNRPD2	19q13.2	0.5	No
RPS9	19q13.4	0	No
GNAS	20q13.3	5.5	Yes
PTPN1	20q13.1	3.5	Yes
NTSR1	20q13	2	Yes
NELFCD	20q13.33	1	Yes
TFAP2C	20q13.2	0.5	Yes
CTSZ	20q13.33	0.5	Yes
PRPF6	20q13.33	0	Yes
PSMA7	20q13.33	0	Yes
RPS21	20q13.33	0	Yes
CHRNA4	20q13.33	0	Yes
KCNB1	20q13.2	2.5	No
PIGA	Xp22.1	1	No
PIR	Xp22.2	0.5	No

1 Gene Ranker scores are from http://cbio.mskcc.org/tcga-generanker/index.jsp.

2 Indicates whether or not the gene is located in a region of recurrent amplification as determined by GISTIC analysis of 10844 samples in the TCGA database (TCGA database version 2015-06-01 stddata).

**Table 2:** SUM-185 copy number amplified genes that are hits in the shRNA screen

Gene symbol	Locus	Gene ranker score^1^	GISTIC^2^
FGFR3	4p16.3b	8.5	Yes
CTBP1	4p16.3c	2.5	Yes
ANKRD17	4q13.3d	1	Yes
IDUA	4p16.3c	2	No
GPT2	16q11.2i	1	No
ISYNA1	19p13.11c	1	Yes
HSPBP1	19q13.42b	0	Yes
NDUFA13	19p13.11a	2.25	No
BCL2L1	20q11.21b	3.5	Yes
ID1	20q11.21b	2.5	Yes
POFUT1	20q11.21b	2.25	Yes
COX4I2	20q11.21b	1	Yes
ACSS1	20p11.21a	2	No
SSTR4	20p11.21c	0	No
NKX2-2	20p11.22b	0	No

1 Gene Ranker scores are from http://cbio.mskcc.org/tcga-generanker/index.jsp.

2 Indicates whether or not the gene is located in a region of recurrent amplification as determined by GISTIC analysis of 10844 samples in the TCGA database (TCGA database version 2015-06-01 stddata).

**Table 3:** SUM-190 copy number amplified genes that are hits in the shRNA screen

Gene symbol	Locus	Gene ranker score^1^	GISTIC^2^
CXCL5	4q13.3d	0	Yes
EPHA5	4q13.1f	3.25	No
STK3	8q22.2a	1	Yes
C8ORF59	8q21.2b	0	Yes
PLEKHF2	8q22.1c	0	Yes
RPL30	8q22.2a	0	Yes
SPAG1	8q22.2b	0	Yes
LRP12	8q22.3d	0	Yes
EMC2	8q23.1d	0	Yes
RTN4RL2	11q12.1a	0	No
UBN1	16p13.3b	0	No
NR1D1	17q21.1c	1	Yes
FNDC8	17q12a	0	No
RPL27	17q21.31a	0	No
TMEM106A	17q21.31b	0	No
MTCP1	Xq28h	2	Yes

1 Gene Ranker scores are from http://cbio.mskcc.org/tcga-generanker/index.jsp.

2 Indicates whether or not the gene is located in a region of recurrent amplification as determined by GISTIC analysis of 10844 samples in the TCGA database (TCGA database version 2015-06-01 stddata).

**Table 4:** SUM-225 copy number amplified genes that are hits in the shRNA screen

Gene symbol	Locus	Gene ranker score^1^	GISTIC^2^
CDKL2	4q21.1a	1.5	Yes
CXCL3	4q13.3d	0	Yes
CYP51A1	7q21.2a	2	Yes
ANKIB1	7q21.2a	1	Yes
RAD21	8q24.11a	2.5	Yes
GPT	8q24.3h	2.25	Yes
ST3GAL1	8q24.22c	2	Yes
CYP11B1	8q24.3f	2	Yes
HSF1	8q24.3g	1.25	Yes
HNF4G	8q21.11c	1	Yes
TAF2	8q24.12b	1	Yes
CCNE2	8q22.1c	0	Yes
RPL30	8q22.2a	0	Yes
EIF3E	8q23.1c	0	Yes
ENY2	8q23.1d	0	Yes
EIF3H	8q24.11a	0	Yes
MED30	8q24.11b	0	Yes
WISP1	8q24.22c	0	Yes
TSNARE1	8q24.3e	0	Yes
MAF1	8q24.3g	0	Yes
BOP1	8q24.3g	0	Yes
PUF60	8q24.3g	0	Yes
ZNF707	8q24.3g	0	Yes
VPS28	8q24.3h	0	Yes
RPL8	8q24.3h	0	Yes
DNM1L	12p11.21a	1	Yes
POLR2C	16q13c	2	No
ATP6V0D1	16q22.1b	1	No
CAPNS2	16q12.2c	0	No
MT1B	16q13b	0	No
MT1M	16q13b	0	No
CNOT1	16q21a	0	No
CTRL	16q22.1b	0	No
ERBB2	17q12c	9.25	Yes
GRB7	17q12c	1	Yes

1 Gene Ranker scores are from http://cbio.mskcc.org/tcga-generanker/index.jsp.

2 Indicates whether or not the gene is located in a region of recurrent amplification as determined by GISTIC analysis of 10844 samples in the TCGA database (TCGA database version 2015-06-01 stddata).

Next, to define functional oncogene signatures with the greatest relevance to human cancer generally, we filtered the list of amplified genes that were hits in the shRNA screen using the Genomic Identification of Significant Targets in Cancer (GISTIC) database (http://www.broadinstitute.org/tcga/home) [18, 19] and the cancer Gene Ranker database (http://cbio.mskcc.org/tcga-generanker/batch.jsp) (Tables 1–4). This approach allowed us to focus on candidate oncogenes that are located in regions of recurrent amplification in primary human cancers as defined by GISTIC, and are considered to be important cancer genes as determined by Gene Ranker (Gene Ranker score ≥ 1). This analysis again significantly reduced the number of candidate oncogenes in each of the cell lines (Table 5).

**Table 5:** Functional oncogene signatures

	SUM-52	SUM-185	SUM-190	SUM-225
**Amplified**	**FGFR2**	**FGFR3**	**MTCP1**	**ERBB2**
	**CDK6**	**BCL2L1**	**NR1D1**	**RAD21**
	**GNAS**	**ID1**	**STK3**	**GPT**
	**PTPN1**	**CTBP1**		**CYP51A1**
	**KAT6A**	**POFUT1**		**ST3GAL1**
	**TBK1**	**ISYNA1**		**CYP11B1**
	**PTPRH**	**COX4I2**		**CDKL2**
	**NTSR1**	**ANKRD17**		**HSF1**
	**DDX5**			**DNM1L**
	**RPS3**			**GRB7**
	**CD79B**			**ANKIB1**
	**TLK2**			**HNF4G**
	**NDUFB7**			**TAF2**
	**NELFCD**			
	**TFR2**			
	**CPSF4**			
	**STAR**			
**Point Mutated**	**CDKN2A**	**PIK3CA**	**PIK3CA**	**BIRC6**

Point mutations are another mechanism by which oncogenes can become activated. To include oncogenes activated by point mutation in the functional oncogene signatures, we integrated the shRNA screen data with exome sequencing data. Before integrating the exome sequencing data with the shRNA screen data, the exome sequencing data was first filtered using the Catalog of Somatically Mutated Genes in Cancer (COSMIC; cancer.sanger.ac.uk) [20] database to identify only the variants that have been shown to be recurrent somatic mutations in human cancer. In each cell line, this reduced the number of genes harboring mutations to less than 30. In the SUM-190 and SUM-185 cell lines, merging the mutated gene lists with the shRNA screen hits identified a single gene, the well-characterized driver oncogene PIK3CA [21–24]. Both cell lines harbor the H1047R substitution mutation, which is one of the most commonly reported activating mutations for PIK3CA in breast cancer [21, 25, 26]. The SUM-52 and SUM-225 cell lines also each contained a single point mutated gene that was a hit in the shRNA screen although in both cases the identified mutation was only rarely observed in primary cancers. Combining this analysis of point mutated genes with the analysis of copy number amplified genes resulted in a final functional oncogene signature for each cell line (Table 5).

### Oncogene signatures predict sensitivity to molecularly targeted drugs

**Figure 1:**
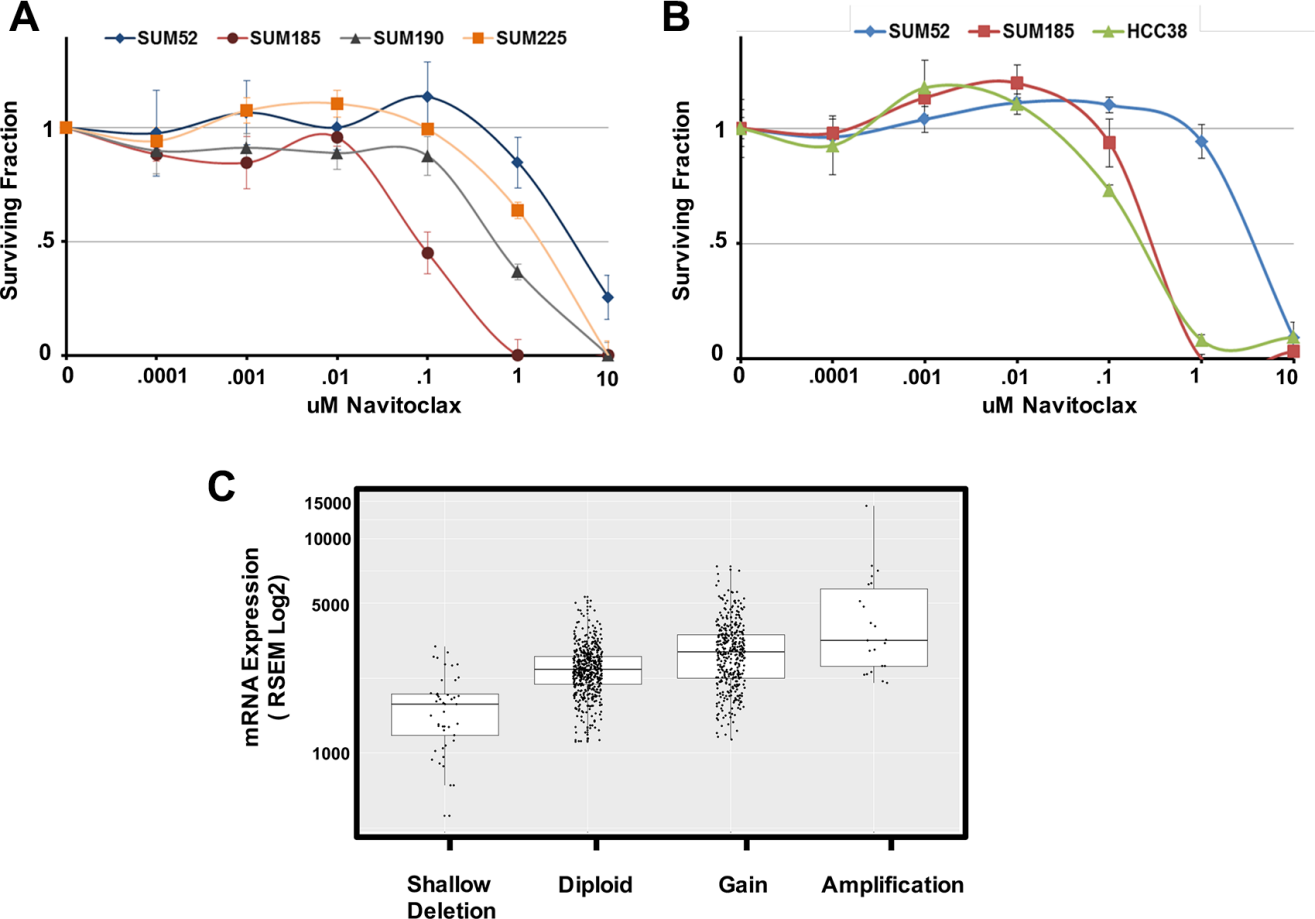
Oncogene signatures predict sensitivity to the BCL2l1 targeted drug Navitoclax (**A**) Cell number for each cell line in the panel expressed as fraction of control. Cells were treated with the indicated concentrations of Navitoclax for 96 hours. (**B**) Cell number for the BCL2L1-amplifeid breast cancer cell line HCC38 in comparison to SUM-185 and SUM-52 cells following exposure to the indicated concentrations of Navitoclax for 96 hours. (**C**) Boxplot of BCL2L1 mRNA expression levels for the 1105 primary breast tumors in the TCGA database. Tumors were grouped based on TCGA analysis of BCL2L1 gene copy number. Data for individual tumors is shown as data points over boxplots.

We hypothesized that the identified functional oncogene signatures would be able to accurately predict sensitivity to targeted therapies. To test this hypothesis, we identified genes in each functional oncogene signature that have an available targeted inhibitor. Six genes were identified overall (BCL2L1, CDK6, PIK3CA, FGFR2, FGFR3, and HER2), with each cell line signature containing at least one druggable driver oncogene. The BCL2L1 gene, which is part of the SUM-185 functional oncogene signature, was of particular interest because a role for amplified BCL2L1 as a driver oncogene in breast cancer has not been previously characterized. We therefore tested the cell line panel for sensitivity to the BCL2L1-targeted drug Navitoclax [27]. As predicted from the functional oncogene signature, SUM-185 cells were highly sensitive to this drug and were the most sensitive cell line in our panel with an IC50 in a growth assay of approximately 0.1 μM (Figure 1A). Because BCL2L1 has not been previously characterized as a driver oncogene in breast cancer, we used the TCGA cell line database (http://www.cbioportal.org/) to identify an additional breast cancer cell line, HCC38, which also harbors a focal amplification of BCL2L1. Interestingly, treatment of HCC38 cells with Navitoclax revealed a high level of sensitivity that was equivalent to that of SUM-185 cells (Figure 1B). Querying the TCGA database for BCL2L1 amplification in primary breast tumors revealed that 430/1105 (39%) samples harbor BCL2L1 copy number gain and 23/1105 (2%) harbor a high-level focal amplification as determined by GISTIC (Figure 1C). Additionally, there is a trend towards increased expression with increasing levels of gene copy number which is a characteristic of driver oncogenes that are activated by amplification (Figure 1C). Taken together, these results suggest that functional oncogene signatures can accurately predict drug sensitivity and specifically support a role for BCL2L1 as a novel driver oncogene and potential therapeutic target in a subset of breast cancers.

**Figure 2:**
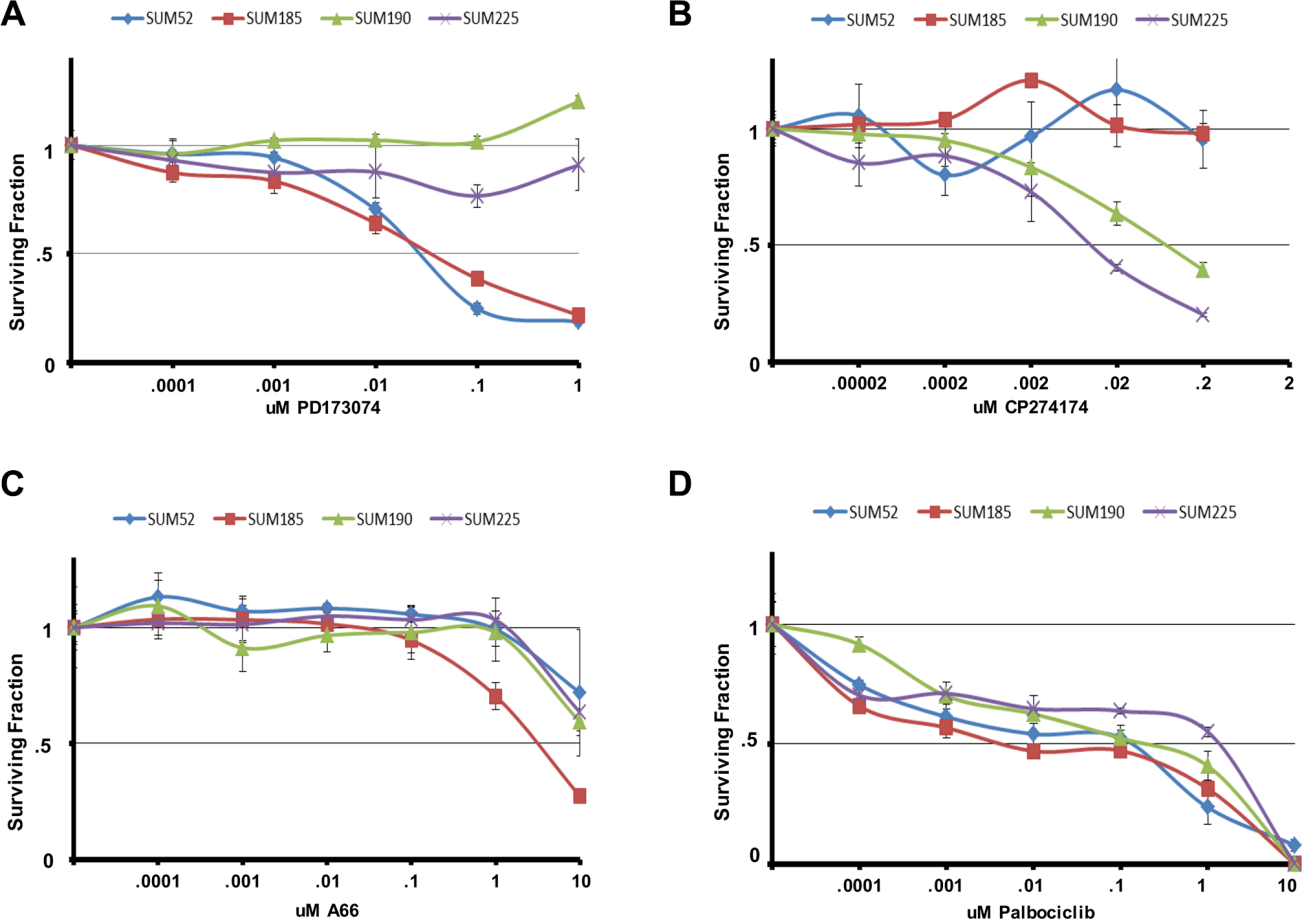
Oncogene signatures predict sensitivity to molecularly targeted drugs Cell number for each cell line in the panel following exposure to the indicated concentrations of (**A**) PD173074 (**B**) CP724714 (**C**) A66 or (**D**) Palbociclib for 96 hours. Cell number is shown as the fraction of control treated cells.

In order to further test the ability of the functional oncogene signatures to predict sensitivity to targeted therapies, we examined the response of our cell line panel to inhibitors that target each of the remaining druggable driver oncogenes. The SUM-52 and SUM-185 cell lines each have FGFR family members in their oncogene signatures and we and others have demonstrated previously the extreme sensitivity of SUM-52 cells to FGFR2 inhibitors [28–30]. Treating the cell line panel with the small molecule FGFR inhibitor, PD173074 [31], showed that SUM-52 and SUM-185 cells were both highly sensitive to this drug with IC50 values ~.05uM (Figure 2A). Thus, for these two cell lines, having an FGFR family member as part of the functional oncogene signature was predictive of sensitivity to the appropriate targeted drug.

The SUM-225 and SUM-190 cell lines were chosen for these studies because they were known to harbor an amplification and overexpression of the HER2 oncogene [14]. Interestingly, whereas HER2 (as well as its neighbor on the amplicon GRB7) was a hit in the SUM-225 shRNA screen, HER2 was not a hit in the SUM-190 screen. Examining the sensitivity of the cell line panel to the HER2 inhibitor CP724721 [32] showed that while SUM-52 and SUM-185 cells were unresponsive to this drug, SUM-225 cells were highly sensitive (Figure 2B). By contrast, while SUM-190 cells were responsive to this inhibitor, the IC50 value was 10-fold greater than for SUM-225 cells. This result helps to explain why HER2 was not a hit in the shRNA screen in SUM-190 cells, as these cells are relatively resistant to targeting HER2.

Examining the sensitivity of the cell line panel to the small molecule PIK3CA inhibitor A66 showed that SUM-185 cells which harbor an activating PIK3CA mutation were indeed, more than 10-fold more sensitive to A66 than any of the other cell lines, including SUM-190 (Figure 2C). The SUM-190 cells exhibited the same sensitivity to this drug as SUM-52 and SUM-225 cells even though they also harbor an activating PIK3CA mutation. It is possible that the presence of PIK3CA mutations in the context of HER2 amplifications could result in reduced sensitivity to either drug target alone by compensating for each other in the presence of a single agent [33].

Finally, since CDK6 was part of the functional oncogene signature in SUM-52 cells, we examined the cell line panel for sensitivity to the CDK4/6 inhibitor palbociclib [34]. All four cell lines displayed similar sensitivity to palbociclib thus, in this case, CDK6 being a functional oncogene did not predict enhanced sensitivity to palbociclib (Figure 2D). Looking only at shRNA screen data, CDK6 was a hit in both the SUM-52 and SUM-185 cell lines but not in the SUM-190 or SUM-225 cell lines. Therefore, the shRNA screen data alone was also not predictive of response to palbociclib.

### Oncogene signatures guide rational combination therapies that synergistically induce cancer cell death

Beyond predicting sensitivity to individual targeted agents, elucidation of functional oncogene signatures offers an opportunity to correctly predict effective targeted drug combinations. Combining multiple targeted therapies has been proposed as an approach for improving patient response rates and also for serving as a barrier to the development of resistance [35–42]. We therefore performed experiments to determine if targeting multiple oncogenes in a signature would yield synergistic interactions specific to an individual cell line. For these experiments we performed clonogenic survival assays (colony-forming assays) to determine if drug combinations could have irreversible effects on the proliferative capacity of the cells. We have previously demonstrated that for SUM-52 cells, 72 hour exposure to the FGFR inhibitor PD173074, while resulting in complete inhibition of proliferation, had only a small effect on colony-forming ability once the drug was removed [17], indicating that the effects of this drug on growth were reversible. We have reported similar results with other small molecule kinase inhibitors in other breast cancer cell lines [43]. Thus, the experiments reported here were aimed at identifying drug combinations that would result in irreversible effects on growth and clonogenicity.

**Figure 3:**
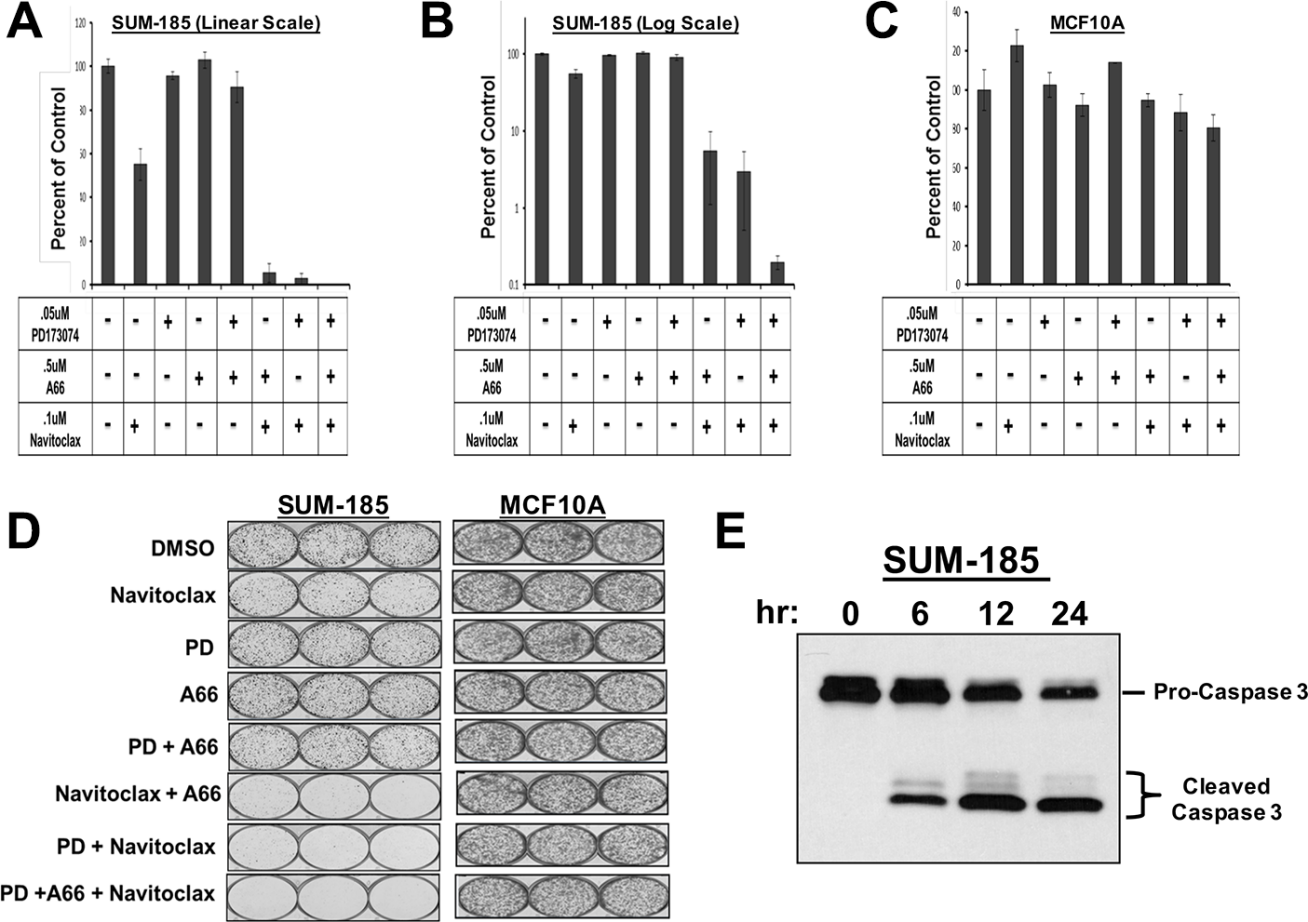
Oncogene signatures guide rational combination therapies that synergistically induce cancer cell death (**A**) Clonogenic survival results for SUM-185 cells treated with the indicated small molecule inhibitors for 72 hours. Colonies were stained and counted when colony sizes reached 50–100 cells per colony. Colony counts were normalized to DMSO treated cells. (**B**) Data from panel A plotted on a semi-log scale. (**C**) Clonogenic survival assay results for MCF10A cells treated with the indicated small molecule inhibitors. Colony counts were normalized to DMSO treated cells. (**D**) Images of colony forming assays shown in panels (A–C). (**E**) Western blot analysis of Caspase 3 following treatment of SUM-185 cells for 6, 12, or 24 hours with the three drug combination of.05 uM PD173074,.5 uM A66 and.1 uM Navitoclax.

The SUM-185 cell line functional oncogene signature suggested an obvious combination strategy involving small molecule inhibitors that target each of three oncogenes in the signature, FGFR3, PIK3CA and BCL2L1. We therefore performed a clonogenic survival assay in which SUM-185 cells were treated with IC50 concentrations of the FGFR, PI3′K, or BCL2L1 inhibitors alone, or the two- and three-drug combinations. As expected from previous experiments, we observed that treatment with individual drugs resulted in little or no effect on colony forming ability (Figure 3A and 3B). In stark contrast, combined treatment of SUM-185 cells with IC50 concentrations of the drugs that target the three functional oncogenes resulted in an 800 fold reduction in colony forming (Figure 3A, 3B and 3D). Examining the results of the two-drug combination treatments revealed that combining Navitoclax with either the FGFR or the PI3′kinase inhibitor reduced colony-forming ability by approximately 50-fold. The similarity of the results for the FGFR and PI3′K inhibitors in combination with the BCL2L1 inhibitor is in agreement with our recently published results showing that in SUM-185 cells, FGFR3 and PIK3CA both function to drive AKT phosphorylation [44]. These results suggest that, in the context of a functional oncogene signature, targeting a driver oncogene that mediates AKT phosphorylation in combination with targeting BCL2L1 has synergistic effects on cell viability even when the drugs are used at low doses. Importantly, treatment of the non-transformed breast epithelial cell line MCF10A with these drug combinations showed no effect on colony-forming ability (Figure 3C and 3D), demonstrating the specificity of this response for transformed cells. Treatment of SUM-185 cells with the three-drug combination induced changes in cell morphology indicative of an apoptotic response (Supplementary Figure S1) and subsequent examination of cell extracts indeed showed potent activation of caspase 3 even at the earliest time point (Figure 3E) suggesting that the observed loss of cell viability was due to induction of apoptosis.

### Testing combinatorial treatment strategies including an inhibitor of BCL2L1 in multiple breast cancer cell lines

**Figure 4:**
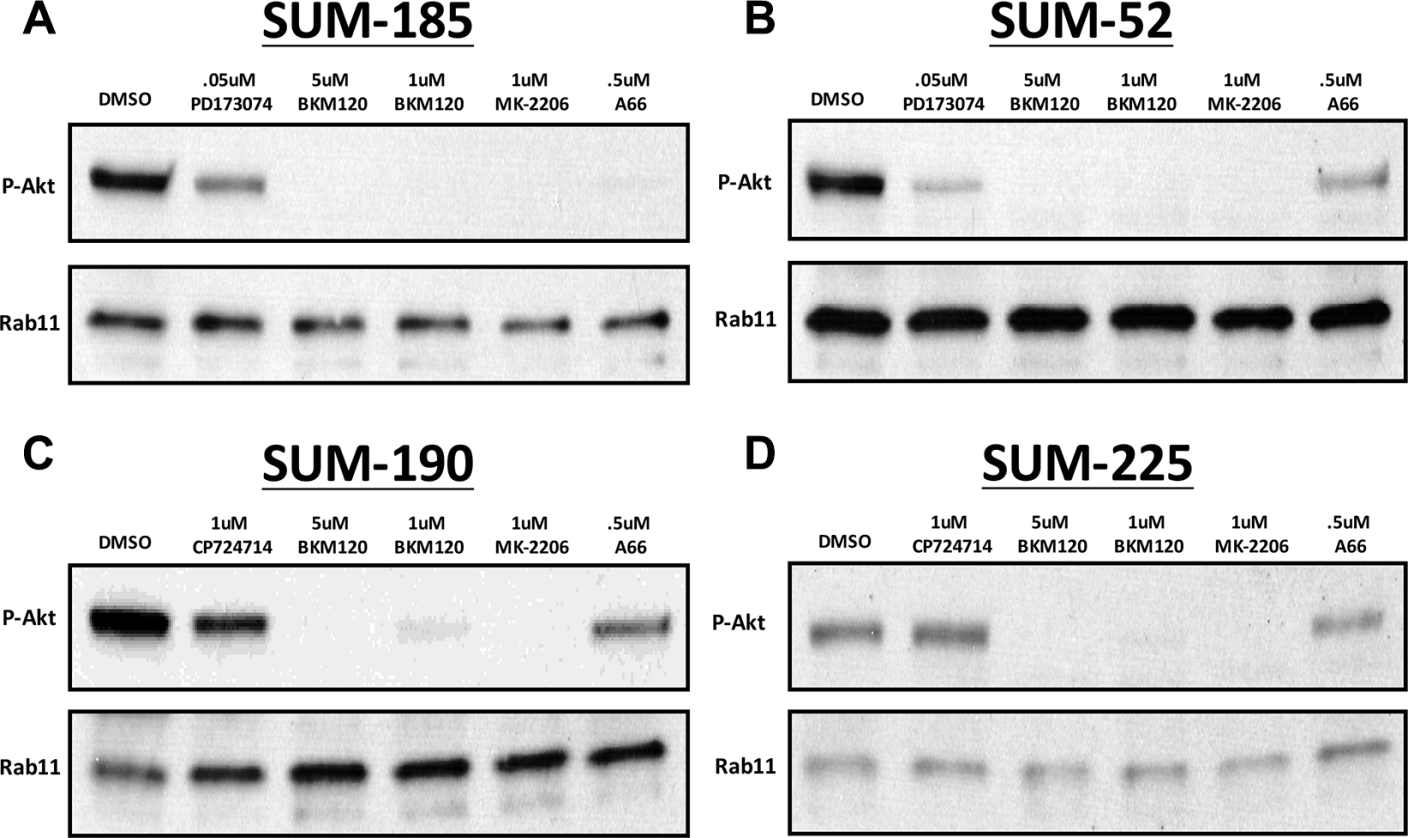
Identification of small molecule inhibitors that inhibit AKT phosphorylation in each of the cell lines in the panel Western blot analysis of phospho-AKT (Ser473) protein levels following treatment with the indicated small molecule inhibitors in (**A**) SUM-185 (**B**) SUM-52 (**C**) SUM-190 or (**D**) SUM-225 cells. Western blot analysis of Rab11 protein levels served as a protein loading control.

**Figure 5:**
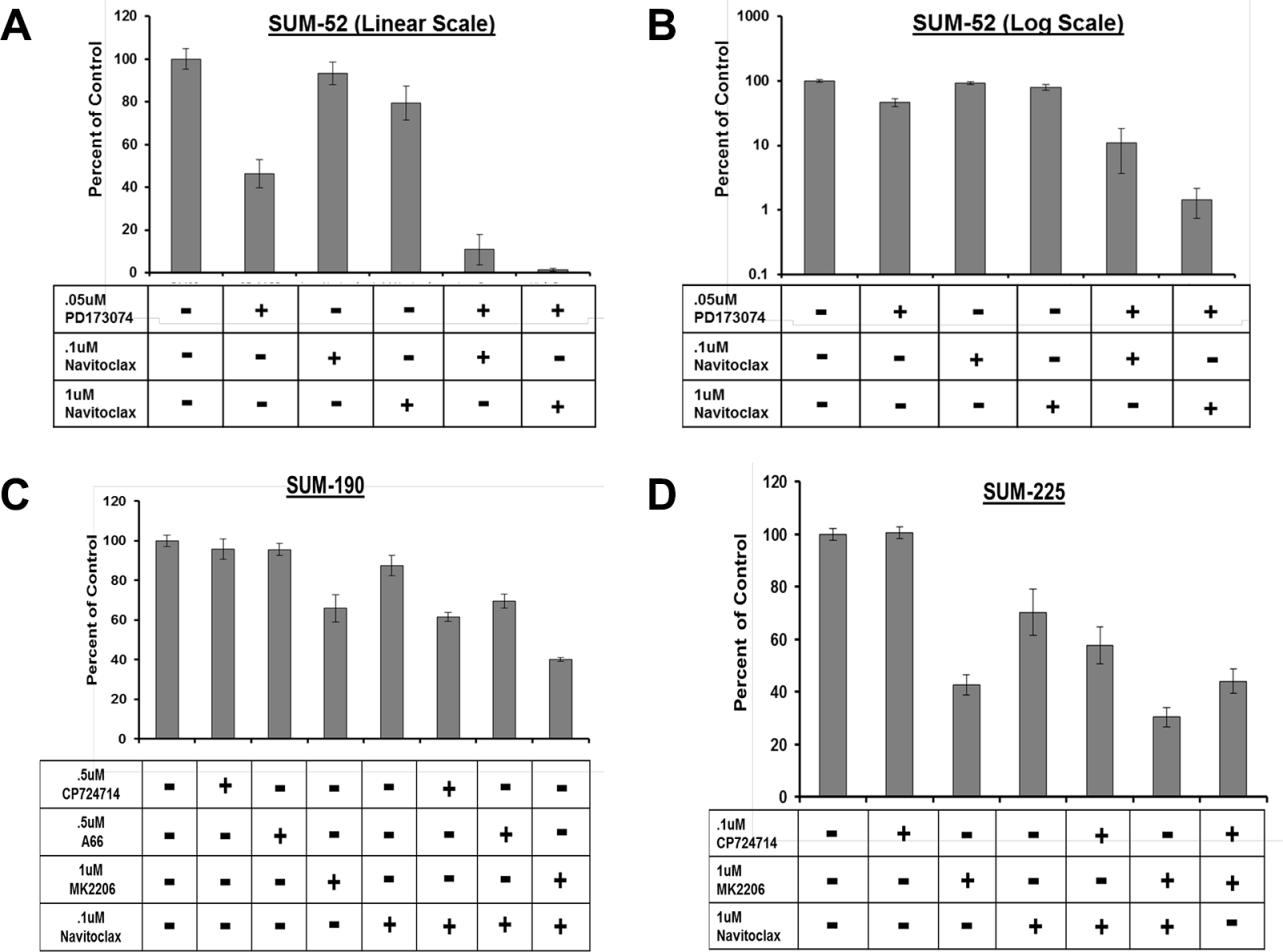
Combination treatment strategy targeting BCL2L1 activity and AKT phosphorylation in multiple breast cancer cell lines Clonogenic survival analysis for (**A**) SUM-52 (**B**) SUM-52 plotted on a semi-log scale (**C**) SUM-190 or (**D**) SUM-225. Cells were treated with the indicated small molecule inhibitors for 72 hours. Colonies were stained and counted when colony sizes reached 50–100 cells per colony. Colony counts were normalized to control treated cells.

Based on the results described above, we hypothesized that inhibition of AKT phosphorylation in combination with BCL2L1 inhibition may induce synergistic cancer cell death in other cancer cell lines. In order to test this hypothesis, we first identified small molecule inhibitors that were capable of inhibiting AKT phosphorylation in each of the cell lines in our panel (Figure 4). For these experiments, we examined levels of AKT phosphorylation in each cell line following treatment with an inhibitor of the RTK oncogene (PD173074 or CP724721), a pan-PI3′K inhibitor (BKM-120), a PIK3CA specific inhibitor (A66), or an allosteric AKT inhibitor (MK-2206). In SUM-52 cells, treatment with the FGFR inhibitor resulted in effective inhibition of AKT phosphorylation similar to what was observed in SUM-185 cells (Figure 4A and 4B). We therefore tested the effect of the FGFR/BCL2L1 combination therapy in a clonogenic survival assay on SUM-52 cells but because SUM-52 cells were more resistant than SUM-185 cells to the BCL2L1 inhibitor (Figure 1A and 1B), we tested Navitoclax at both 1 μM and at the IC50 concentration for SUM-185 cells of 0.1 μM. The results of this experiment demonstrated that, similar to what we observed for SUM-185 cells, there was a synergistic effect of the FGFR/BCL2L1 drug combination on colony-forming ability (Figure 5A and 5B). The synergy was more profound at the higher Navitoclax dose of 1 μM however, it should be noted that this level of Navitoclax treatment also reduces colony-forming ability in the non-transformed MCF10A cell line (data not shown).

In SUM-190 cells, treatment with a 1uM dose of the allosteric AKT inhibitor MK-2206 effectively inhibits AKT phosphorylation (Figure 5C). Combined treatment of SUM-190 cells with 1uM MK-2206 and the BCL2L1 inhibitor did not result in a synergistic effect on colony-forming ability. Similarly, in SUM-225 cells 1uM MK-2206 effectively inhibited AKT phosphorylation (Figure 5D) and combining this treatment with the BCL2L1 inhibitor did not result in a synergistic effect. These results indicate that the synergistic response to dual inhibition of BCL2L1 activity and AKT phosphorylation is cancer cell line-specific and can be predicted by the presence of drug targets in the cell line functional oncogene signature.

## DISCUSSION

In this study, we defined functional oncogene signatures for a panel of breast cancer cell lines that share the common feature of each having one known amplified and over-expressed RTK oncogene. To define functional oncogene signatures, we integrated genomic profiling for each cell line with genome-wide functional genetic screens and data from cancer knowledge databases. This analysis identified known as well as novel driver oncogenes for each cell line. Using the functional oncogene signatures, we accurately predicted sensitivity to small molecule inhibitors and rationally designed combination therapies that synergistically induced cancer cell death.

In most, but not all cases, the functional oncogene signatures accurately predicted increased sensitivity to drugs that target functional oncogenes. Thus, the SUM-52 and SUM-185 signatures contain FGFR2 and FGFR3 respectively and these cells are exquisitely sensitive to the small molecule FGFR inhibitor PD173074. Similarly, SUM-185 cells, which have a PIK3CA mutation were most sensitive to the PI3′K inhibitor A66. SUM-225 cells, with HER2 (and GRB7) in the oncogene signature were highly sensitive to the HER2 specific drug CP274174. Interestingly, SUM-190 cells also have a HER2 amplification and over-expression, but neither HER2 nor GRB7 were hits in the screen, and thus neither gene is part of the SUM-190 functional oncogene signature. SUM-190 cells, while responsive to the HER2 inhibitor, were 10-fold more resistant than SUM-225 cells. SUM-190 cells also harbor an activating PIK3CA mutation and, surprisingly, were no more sensitive to PI3′K inhibitors than other cell lines without PIK3CA mutations. Also, in that regard, CDK6 amplification was not predictive of increased sensitivity to palbociclib.

Among the novel breast cancer oncogenes identified in this study was the anti-apoptotic protein BCL2L1. BCL2L1 plays a role in promoting survival in several solid tumor types [45–48] and has been recently implicated as an amplified driver oncogene in colorectal cancer and gastric cancer [18, 49–51]. Small molecules that bind to and inhibit the anti-apoptotic function of BCL2L1 and the related BCL2 protein have been developed and are currently in phase I/II clinical trials. In our study, we observed that BCL2L1 was amplified and over-expressed in the SUM-185 cell line and was also a strong hit in the functional genetic screen (5th ranked gene out of 1266 hits). Growth assay experiments showed that SUM-185 cells were highly sensitive to the BCL2L1 inhibitor Navitoclax, which is consistent with a role for BCL2L1 as a driver oncogene in this cell line. We also identified an additional breast cancer cell line with amplification and over-expression of BCL2L1 and showed that this cell line was similarly sensitive to BCL2L1 inhibition. Data from the TCGA database indicates that a significant portion of breast cancers harbor amplification and/or overexpression of BCL2L1. These results suggest that BCL2L1 is a driver oncogene in a subset of breast cancers and that those patients with BCL2L1 as part of their oncogene signature may benefit from therapies targeting BCL2L1.

We also found that when multiple oncogenes in the oncogene signature were targeted, cells were not only inhibited in their proliferation, but their clonogenic capacity was dramatically reduced. This was particularly true when a drug that targeted a driving oncogene that resulted in down-regulation of AKT-phosphorylation was combined with inhibition of BCL2L1. In a previous study, we demonstrated that amplified FGFR2 and mutant PIK3CA were linked to AKT phosphorylation in SUM-185 cells, and that targeting either oncogene alone induced potent inhibition of cell proliferation. However, this growth inhibition was reversible as indicated by small decreases in clonogenic capacity following drug removal [44]. By contrast, simultaneous inhibition of FGFR2 and BCL2L1, or PIK3CA and BCL2L1 in SUM-185 cells resulted in dramatic reductions in clonogenic survival. Interestingly, targeting BCL2L1 alone at concentrations that inhibited cell proliferation had only marginal effects on clonogenic capacity. This result suggests that AKT activity can support cell survival in the presence of BCL2L1 inhibition, and vice versa, but that simultaneous inhibition of both oncogenes results in profound and irreversible loss of proliferative capacity. Extending this result to SUM-52 cells which do not harbor BCL2L1 amplification but in which BCL2L1 was a hit in the shRNA screen, we found that we could induce synergistic loss of clonogenic capacity by combining the FGFR inhibitor with Navitoclax, but because BCL2L1 was not part of the oncogene signature in these cells, higher concentrations of drug were required to achieve a strong effect. Finally, combined inhibition of BCL2L1 activity and AKT phosphorylation did not result in synergistic effects in either the SUM-190 or SUM-225 cell lines, which is consistent with the failure of BCL2L1 to be a hit in the shRNA screen in these cell lines. Synergistic responses are therefore cell type-specific and depend on the addiction of the breast cancer cells to the targeted functional oncogenes.

Recently, Engelman et al. performed a genome-wide screen to identify genes that, when inhibited, cooperate with MEK inhibitors to kill KRAS mutant cancer cells [52]. The top hit identified in this screen was BCL2L1. Grant et al. [53] also observed potent synergy from combining BCL2L1 inhibitors with inhibition of AKT in leukemias and have suggested this as a strategy to treat AML. Taken together, these results and ours provide strong rationale for the development of combination therapies that target BCL2L1 and AKT, particularly in light of the observed synergy which allows for the use of very low doses of the targeted drugs.

AKT is an important protein that regulates, among other things, cell survival, and is considered a potential therapeutic target. However, given the importance of AKT activity in many cellular pathways, targeting AKT directly is often associated with significant side effects. Thus, it is preferable, from a therapeutic standpoint, to inactivate AKT indirectly by targeting the oncogene that is responsible for its activity. This approach will allow cancer cell-specific inactivation of AKT, which is vital to obtaining a high therapeutic index. Our results demonstrate that functional oncogene signatures can identify driver oncogenes that are responsible for driving AKT signaling and in turn allow for rational targeting of AKT through these drivers.

A personalized medicine approach for cancer treatment holds the promise of treating patients with highly effective targeted therapeutics that are tailored to the genetic alterations that drive their cancer. Realizing this promise will require accurate identification of the complete set of oncogenes to which each patient's cancer cells are addicted. Understanding the complete set of activated oncogenes that drive a patients’ tumor will allow for rationally designed combination therapies that induce profound decreases in clonogenic survival with minimal effects on normal cells and tissues.

## MATERIALS AND METHODS

### Reagents and cell lines

All inhibitors were purchased from Selleckchem. The PathScan^®^ (#5301) and Caspase-3(#9665) antibodies were purchased from Cell Signaling. The SUM breast cancer cell lines were maintained as described previously [14, 54, 55]. MCF10A cells were a gift from Dr. Herb Soule at the Michigan Cancer Foundation [56].

### Array comparative genomic hybridization

Microarrays with an average resolution of 35 kb (Agilent Human Genome CGH Microarray 44k chip) were hybridized after direct labeling of DNA with fluorescent dyes. DNA extraction was performed using standard column purification (Qiagen) and normal human female DNA was used as the reference. Dye-reversed replicates were performed. Regions of chromosomal amplification and deletion were determined based on circular binary segmentation provided by the Bioconductor DNA copy library.

### Genome-scale RNAi-based growth and viability screen

Virus pools expressing shRNA constructs were prepared according to the Cellecta Pooled Lentiviral shRNA Libraries User Manual protocol (www.cellecta.com). HEK 293T cells were transfected with each of the three Cellecta library plasmid DNA pools (Human Modules 1-3) and the Cellecta Ready-to-Use Packaging Mix (Cat #CPCP-K2A). For each module, virus was titered and used to transduce 5 × 10^7 target cells at an MOI of ~.5 in the presence of 5ug/ml polybrene. Following transduction, cells were cultured for 3 days to allow expression of the resistance marker and non-transduced cells were eliminated from the culture by addition of the selective agent puromycin to the growth media at 6 ug/ml for SUM-52 cells or 2 ug/ml for SUM-185, SUM-190, and SUM-225 cells. Three days after the addition of puromycin, cells were trypsinized and one half of the total population was harvested for genomic DNA preparation. This DNA served as the reference time point DNA. The remaining cells were plated and grown for ~5–7 population doublings before harvesting for genomic DNA preparation. Genomic DNA was prepared by phenol:chloroform extraction according to the Cellecta Pooled Lentiviral shRNA Libraries User Manual protocol.

Barcode sequences were amplified from genomic DNA by two rounds of PCR. For first round PCR, 50–100 μg of genomic DNA was used for 4 × 100 μl PCR reactions. Each 100 μl reaction contained 2 μl Titanium Taq polymerase (Clontech Catalog #639210), 200 nM dNTP mix (Clontech Cat# 639210) and 10 μM of each primer (FWDHTS 5′-TTCTCTGGCAAGCAAAAGACGGCATA-3′ and RevHTS1 5′-TAGCCAACGCATCGCACAAGCCA-3′). First round PCR reactions began with activation of the polymerase by incubation at 94°C for 3 minutes followed by 16 cycles of denaturation at 94°C for 30 sec, annealing at 65°C for 10 sec, and elongation at 72°C for 20 sec. Final extension was performed by incubation at 68°C for 2 minutes. First round PCR reactions were pooled and 100 μl was used to seed a 4 × 100 μl second round PCR reaction. Each 100 μl second round reaction contained 2 μl Titanium Taq polymerase, 200 nM dNTP mix and 10 μM of each primer (FWDGEX 5′-CAAGCAGAAGACGGCATACGAGA-3′ and RevGEX 5′-AATGATACGGCGACCACCGAGA-3′). Second round PCR reactions began with activation of the polymerase by incubation at 94°C for 3 minutes followed by 12 to 16 cycles of denaturat′ion at 94°C for 30 sec, annealing at 65°C for 10 sec, and elongation at 72°C for 10 sec. Final extension was performed by incubation at 68°C for 2 minutes.

Amplified barcode sequences were run on a 3.5% agarose gel and purified using a QIAquick Gel Extraction Kit (Qiagen) according to manufacturer's instructions. Isolated barcode sequences were further purified using the PureLink Quick PCR Purification Kit (Invitrogen) according to the manufacturer's instructions. For sequencing, purified barcodes were diluted to.75 ng/μl using buffer EB (Qiagen). Amplicons were clustered at 17 pM including 30% (v/v) PhiX to add sequence diversity. Single end (SE) clustering was performed on a Cbot according to the manufacturer's protocol (Illumina, San Diego, CA). A total of 36 cycles of SE sequencing were performed on an Illumina HiScanSQ. Custom primer GexSeqS (5′ AGAGGTTCAGAGTTCTACAGTCCGAA-3′, HPLC Purified) was added to the Illumina sequencing primers at 0.5 μM. Fastq files were generated using CASAVA 1.8.2 and processed using Trimmomatic software (www.usadellab.org) to trim read lengths to 18 nucleotides. Trimmed reads were deconvoluted using Cellecta Barcode Analyzer and Deconvoluter software. Fold depletion scores for each shRNA were calculated as the ratio of the read count at the reference time point versus the final time point.

In the Cellecta shRNA library, the vast majority of genes were targeted by either 5 (67%) or 6 different shRNAs (32%), and a small fraction of genes (1%) were targeted by 2 to 69 different shRNAs. The genes targeted by large numbers of shRNAs were housekeeping genes that would be expected to be hits in most, if not all, cell lines tested. Luciferase, which was used as the non-silencing control was also targeted by over 60 shRNAs and helped to establish the range of scores for known negative hits. Because of the varying numbers of shRNAs per gene, simply ranking hits based on the 2nd highest score or on a weighted average of the top two genes would have biased analysis towards genes with a larger number of shRNAs per gene. To account for this potential bias, log-transformed depletion scores and a quantile estimation approach in which the 80th percentile for each gene was calculated from its empirical distribution were used. This avoided the bias induced by the varying number of scores per gene and accounted for the skewness of the empirical distributions. Genes were then ranked by this log-quantile score and the empirical distribution of the log-quantile score was calculated.

To generate a null distribution of log fold-depletion scores, it was assumed that the majority of genes (> 95%) would not be depleted, and their log-quantile scores would have a normal distribution. Based on this assumption, the median of the empirical distribution was used as an estimate of the mean of the null distribution. The estimate of the standard deviation of the null distribution was defined as the 97.5th quantile minus the 2.5th quantile, divided by 4. This was based on the knowledge that 95% of the data in a normally distributed variable falls between +/− two standard deviations from the mean. Using this null distribution, all genes having log-fold depletion scores that were larger than the 95th percentile of the null distribution were identified as ‘hits’. Using this method, all genes that were hits in the screen had at least two, and usually more, shRNAs with depletion scores above the cut-point.

### Exome sequencing

Exome sequencing of SUM cell line DNA was performed essentially as described previously [57]. Briefly, Agilent Sure Select XT reagents were used to prepare sequencing libraries. Hybrid capture was performed using Agilent Sure SelectXT Human All Exon V4+UTRs, and 100 bp paired-end sequencing was performed on a HighSeq2000.

### Small molecule inhibitor dose response assays

Cells were plated in 24-well plates at a density of 15–30,000 cells per well. Cells were allowed to recover for 4 days before being treated in triplicate with the indicated inhibitors or DMSO control every 24 hours for 4 days. On the 5th day cell number was determined by harvesting and counting nuclei on a Z1 Coulter Counter (Beckman Coulter, Brea, CA, USA). To prepare nuclei for counting, cells were washed three times with PBS, incubated on a rocker table with 0.5 ml per well Hepes/MgCl_2_ buffer (0.01 mM HEPES and 0.015 mM MgCl_2_) for 5 minutes and lysed for 10 minutes with ethyl hexadecyldimethylammonium solution.

### Clonogenic survival assays

Cells were seeded at clonal density in triplicate in 6-well plates and treated with the indicated small molecule inhibitors at 24 and 48 hours after plating. At 72 hours cells were washed and cultured in normal growth media until colony sizes reached ~50–100 cells. For staining, colonies were fixed with 1 mL/well 3.7% paraformaldehyde for 20 min at RT. Colonies were stained with 1 mL/well 0.2% crystal violet for 15 minutes at RT and de-stained with dH2O. Colony counts were generated using a GelCount™ colony counter (Oxford Optronix, Oxfordshire, United Kingdom).

### Western blot analysis

Cells were treated with the indicated concentrations of small molecule inhibitors for 15 hours before preparation of whole cell lysates in RIPA buffer (Sigma Aldrich, R0278) containing 1mM Na_3_VO_4_, and 1× Protease Inhibitor cocktail (Calbiochem, 539131), and protein concentrations were measured by Bradford assay (Bio-Rad). Equal amounts of protein were combined with Laemmli sample buffer (BioRad, 161-0747), boiled for 5 minutes and separated on SDS polyacrylamide gels (BioRad). Proteins were transferred to polyvinylidene difluoride (PVDF) membranes using the Trans-Blot Turbo System (Bio-Rad) and membranes were probed overnight at 4°C with the PathScan^®^ antibody cocktail (1:500) or Caspase-3 antibody (1:1000).

## SUPPLEMENTARY MATERIALS FIGURES AND TABLES










