# Supplementary material for: Functional oncogene signatures guide rationally designed combination therapies to synergistically induce breast cancer cell death
Source: Oncotarget. 2016 May 2;7(24):36138–53. doi: 10.18632/oncotarget.9147 (PMC5094989; doi:10.18632/oncotarget.9147)
Supplement: Supplementary file 1 [file oncotarget-07-36138-s001.pdf]

Functional oncogene signatures guide rationally designed combination therapies to synergistically induce breast cancer cell death

Supplementary Materials

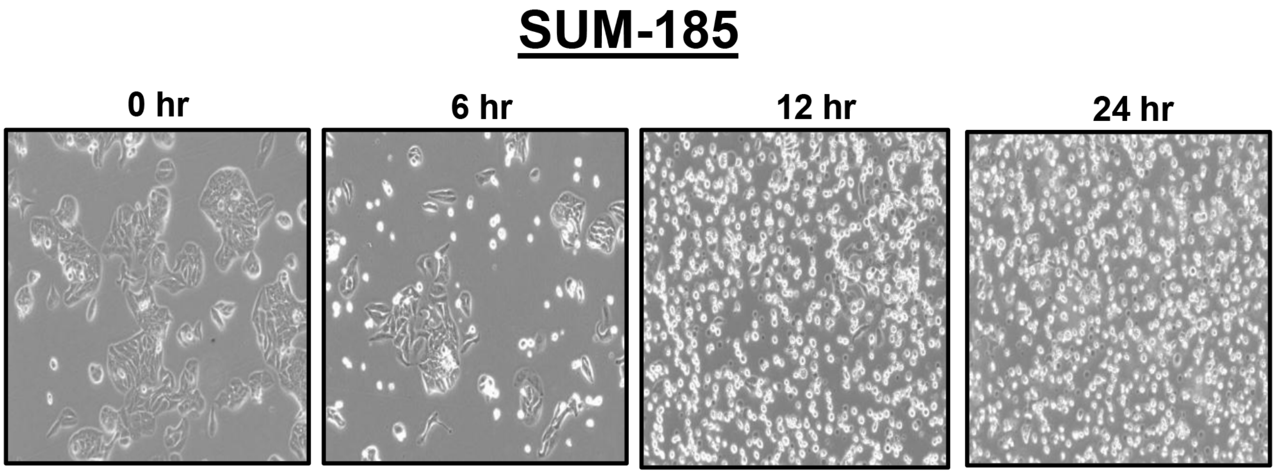

Supplementary Figure S1: Targeting multiple drivers in the SUM-185 functional oncogene signature induces apoptotic cell morphology. Phase contrast images of SUM-185 cells treated with .05 uM PD173074, .1 uM Navitoclax, and .5 uM A66 for the indicated times.

Supplementary Table S1: SUM-52 cell line copy number amplified genes.

Cell line	Symbol	Gene name	Chromosome
SUM52	FLJ45717	FLJ45717 protein	1
SUM52	ZNF669	zinc finger protein 669	1
SUM52	VN1R5	vomeronasal 1 receptor 5	1
SUM52	ZNF670	zinc finger protein 670	1
SUM52	ZNF124	zinc finger protein 124	1
SUM52	TMSL3	thymosin-like 3	4
SUM52	BRI3	brain protein I3	7
SUM52	PTCD1	pentatricopeptide repeat domain 1	7
SUM52	ZNF394	zinc finger protein 394	7
SUM52	DYNC1I1	dynein, cytoplasmic 1, intermediate chain 1	7
SUM52	CPSF4	cleavage and polyadenylation specific factor 4, 30kDa	7
SUM52	ARPC1A	actin related protein 2/3 complex, subunit 1A, 41kDa	7
SUM52	TRRAP	transformation/transcription domain-associated protein	7
SUM52	BUD31	BUD31 homolog (S. cerevisiae)	7
SUM52	PDAP1	PDGFA associated protein 1	7
SUM52	SLC25A13	solute carrier family 25, member 13 (citrin)	7
SUM52	SHFM1	split hand/foot malformation (ectrodactyly) type 1	7
SUM52	ACN9	ACN9 homolog (S. cerevisiae)	7
SUM52	DKFZP434B0335	DKFZP434B0335 protein	7
SUM52	ATP5J2	ATP synthase, H+ transporting, mitochondrial F0 complex, subunit F2	7
SUM52	SMURF1	SMAD specific E3 ubiquitin protein ligase 1	7
SUM52	ZNF789	zinc finger protein 789	7
SUM52	PON2	paraoxonase 2	7
SUM52	ZKSCAN5	zinc finger with KRAB and SCAN domains 5	7
SUM52	ASNS	asparagine synthetase	7
SUM52	BAIAP2L1	BAI1-associated protein 2-like 1	7
SUM52	ZNF498	zinc finger protein 498	7
SUM52	ARPC1B	actin related protein 2/3 complex, subunit 1B, 41kDa	7
SUM52	PON3	paraoxonase 3	7
SUM52	TMEM130	transmembrane protein 130	7
SUM52	NPTX2	neuronal pentraxin II	7
SUM52	BHLHB8	basic helix-loop-helix domain containing, class B, 8	7
SUM52	PDK4	pyruvate dehydrogenase kinase, isozyme 4	7
SUM52	DLX6	distal-less homeobox 6	7
SUM52	ASB4	ankyrin repeat and SOCS box-containing 4	7
SUM52	LMTK2	lemur tyrosine kinase 2	7
SUM52	DLX5	distal-less homeobox 5	7
SUM52	LOC4951	parvalbumin	7
SUM52	TAC1	tachykinin, precursor 1 (substance K, substance P, neurokinin 1, neurokinin 2, neuromedin L, neurokinin alpha, neuropeptide K, neuropeptide gamma)	7
SUM52	AKAP9	A kinase (PRKA) anchor protein (yotiao) 9	7

SUM52	FLJ21062	hypothetical protein FLJ21062	7
SUM52	PFTK1	PFTAIRE protein kinase 1	7
SUM52	GTPBP10	GTP-binding protein 10 (putative)	7
SUM52	PEX1	peroxisome biogenesis factor 1	7
SUM52	MTERF	mitochondrial transcription termination factor	7
SUM52	KRIT1	KRIT1, ankyrin repeat containing	7
SUM52	CDK6	cyclin-dependent kinase 6	7
SUM52	ANKIB1	ankyrin repeat and IBR domain containing 1	7
SUM52	GATAD1	GATA zinc finger domain containing 1	7
SUM52	PON2	paraoxonase 2	7
SUM52	STEAP1	six transmembrane epithelial antigen of the prostate 1	7
SUM52	FAM133B	family with sequence similarity 133, member B	7
SUM52	PEG10	paternally expressed 10	7
SUM52	STEAP2	six transmembrane epithelial antigen of the prostate 2	7
SUM52	DKFZP564O0523	hypothetical protein DKFZp564O0523	7
SUM52	SGCE	sarcoglycan, epsilon	7
SUM52	PON3	paraoxonase 3	7
SUM52	CYP51A1	cytochrome P450, family 51, subfamily A, polypeptide 1	7
SUM52	BET1	BET1 homolog (S. cerevisiae)	7
SUM52	CCDC132	coiled-coil domain containing 132	7
SUM52	CASD1	CAS1 domain containing 1	7
SUM52	GNG11	guanine nucleotide binding protein (G protein), gamma 11	7
SUM52	GNGT1	guanine nucleotide binding protein (G protein), gamma transducing activity polypeptide 1	7
SUM52	PON1	paraoxonase 1	7
SUM52	PPP1R9A	protein phosphatase 1, regulatory (inhibitor) subunit 9A	7
SUM52	FZD1	frizzled homolog 1 (Drosophila)	7
SUM52	CALCR	calcitonin receptor	7
SUM52	TFPI2	tissue factor pathway inhibitor 2	7
SUM52	SAMD9L	sterile alpha motif domain containing 9-like	7
SUM52	SAMD9	sterile alpha motif domain containing 9	7
SUM52	ZNF804B	zinc finger protein 804B	7
SUM52	COL1A2	collagen, type I, alpha 2	7
SUM52	LOC253012	hypothetical protein LOC253012	7
SUM52	MGC26647	hypothetical protein MGC26647	7
SUM52	POP7	processing of precursor 7, ribonuclease P/MRP subunit (S. cerevisiae)	7
SUM52	CYP3A7	cytochrome P450, family 3, subfamily A, polypeptide 7	7
SUM52	TAF6	TAF6 RNA polymerase II, TATA box binding protein (TBP)-associated factor, 80kDa	7
SUM52	ARS2	ARS2 protein	7
SUM52	PILRB	paired immunoglobin-like type 2 receptor beta	7
SUM52	MCM7	minichromosome maintenance complex component 7	7
SUM52	COPS6	COP9 constitutive photomorphogenic homolog subunit 6 (Arabidopsis)	7

SUM52	PILRA	paired immunoglobin-like type 2 receptor alpha	7
SUM52	AP4M1	adaptor-related protein complex 4, mu 1 subunit	7
SUM52	SLC12A9	solute carrier family 12 (potassium/chloride transporters), member 9	7
SUM52	ZKSCAN5	zinc finger with KRAB and SCAN domains 5	7
SUM52	C7orf47	chromosome 7 open reading frame 47	7
SUM52	MEPCE	methylphosphate capping enzyme	7
SUM52	TSC22D4	TSC22 domain family, member 4	7
SUM52	ZNHIT1	zinc finger, HIT type 1	7
SUM52	HRBL	HIV-1 Rev binding protein-like	7
SUM52	ZNF3	zinc finger protein 3	7
SUM52	STAG3	stromal antigen 3	7
SUM52	ZKSCAN1	zinc finger with KRAB and SCAN domains 1	7
SUM52	LOC255374	similar to hypothetical protein MGC49416	7
SUM52	RABL5	RAB, member RAS oncogene family-like 5	7
SUM52	ZCWPW1	zinc finger, CW type with PWWP domain 1	7
SUM52	ZNF498	zinc finger protein 498	7
SUM52	CYP3A4	cytochrome P450, family 3, subfamily A, polypeptide 4	7
SUM52	FIS1	fission 1 (mitochondrial outer membrane) homolog (S. cerevisiae)	7
SUM52	MGC40499	PRotein Associated with Tlr4	7
SUM52	MOSPD3	motile sperm domain containing 3	7
SUM52	TRIM56	tripartite motif-containing 56	7
SUM52	TRIM4	tripartite motif-containing 4	7
SUM52	TFR2	transferrin receptor 2	7
SUM52	PVRIG	poliovirus receptor related immunoglobulin domain containing	7
SUM52	AP1S1	adaptor-related protein complex 1, sigma 1 subunit	7
SUM52	ZSCAN21	zinc finger and SCAN domain containing 21	7
SUM52	ACTL6B	actin-like 6B	7
SUM52	ZAN	zonadhesin	7
SUM52	GNB2	guanine nucleotide binding protein (G protein), beta polypeptide 2	7
SUM52	EPO	erythropoietin	7
SUM52	CYP3A5	cytochrome P450, family 3, subfamily A, polypeptide 5	7
SUM52	ACHE	acetylcholinesterase (Yt blood group)	7
SUM52	EPHB4	EPH receptor B4	7
SUM52	MOGAT3	monoacylglycerol O-acyltransferase 3	7
SUM52	PLOD3	procollagen-lysine, 2-oxoglutarate 5-dioxygenase 3	7
SUM52	GJE1	gap junction protein, epsilon 1, 29kDa	7
SUM52	GATS	opposite strand transcription unit to STAG3	7
SUM52	LOC402573	hypothetical LOC402573	7
SUM52	VGF	VGF nerve growth factor inducible	7
SUM52	C7orf43	chromosome 7 open reading frame 43	7
SUM52	GPC2	glypican 2	7

SUM52	SERPINE1	serpin peptidase inhibitor, clade E (nexin, plasminogen activator inhibitor type 1), member 1	7
SUM52	AZGP1	alpha-2-glycoprotein 1, zinc-binding	7
SUM52	FBXO24	F-box protein 24	7
SUM52	MYLC2PL	myosin light chain 2, precursor lymphocyte-specific	7
SUM52	PERQ1	PERQ amino acid rich, with GYF domain 1	7
SUM52	CLDN15	claudin 15	7
SUM52	CYP3A43	cytochrome P450, family 3, subfamily A, polypeptide 43	7
SUM52	GAL3ST4	galactose-3-O-sulfotransferase 4	7
SUM52	TRIP6	thyroid hormone receptor interactor 6	7
SUM52	EMID2	EMI domain containing 2	7
SUM52	C7orf51	chromosome 7 open reading frame 51	7
SUM52	MUC17	mucin 17, cell surface associated	7
SUM52	PCOLCE	procollagen C-endopeptidase enhancer	7
SUM52	CD36	CD36 molecule (thrombospondin receptor)	7
SUM52	HGF	hepatocyte growth factor (hepapoietin A; scatter factor)	7
SUM52	SEMA3E	sema domain, immunoglobulin domain (Ig), short basic domain, secreted, (semaphorin) 3E	7
SUM52	CACNA2D1	calcium channel, voltage-dependent, alpha 2/delta subunit 1	7
SUM52	SEMA3C	sema domain, immunoglobulin domain (Ig), short basic domain, secreted, (semaphorin) 3C	7
SUM52	ZNF479	zinc finger protein 479	7
SUM52	ZNF107	zinc finger protein 107	7
SUM52	ZNF138	zinc finger protein 138	7
SUM52	ZNF680	zinc finger protein 680	7
SUM52	CHCHD2	coiled-coil-helix-coiled-coil-helix domain containing 2	7
SUM52	SEMA3A	sema domain, immunoglobulin domain (Ig), short basic domain, secreted, (semaphorin) 3A	7
SUM52	SEMA3D	sema domain, immunoglobulin domain (Ig), short basic domain, secreted, (semaphorin) 3D	7
SUM52	SEMA3E	sema domain, immunoglobulin domain (Ig), short basic domain, secreted, (semaphorin) 3E	7
SUM52	GRM3	glutamate receptor, metabotropic 3	7
SUM52	CACNA2D1	calcium channel, voltage-dependent, alpha 2/delta subunit 1	7
SUM52	CUTL1	cut-like 1, CCAAT displacement protein (Drosophila)	7
SUM52	SH2B2	SH2B adaptor protein 2	7
SUM52	MYLC2PL	myosin light chain 2, precursor lymphocyte-specific	7
SUM52	EMID2	EMI domain containing 2	7
SUM52	INDO	indoleamine-pyrrole 2,3 dioxygenase	8
SUM52	C8orf4	chromosome 8 open reading frame 4	8
SUM52	PPAPDC1B	phosphatidic acid phosphatase type 2 domain containing 1B	8
SUM52	TM2D2	TM2 domain containing 2	8
SUM52	EIF4EBP1	eukaryotic translation initiation factor 4E binding protein 1	8
SUM52	ADAM9	ADAM metallopeptidase domain 9 (meltrin gamma)	8
SUM52	LSM1	LSM1 homolog, U6 small nuclear RNA associated (S. cerevisiae)	8

SUM52	ASH2L	ash2 (absent, small, or homeotic)-like (Drosophila)	8
SUM52	BRF2	BRF2, subunit of RNA polymerase III transcription initiation factor, BRF1-like	8
SUM52	DDHD2	DDHD domain containing 2	8
SUM52	WHSC1L1	Wolf-Hirschhorn syndrome candidate 1-like 1	8
SUM52	PROSC	proline synthetase co-transcribed homolog (bacterial)	8
SUM52	FKSG2	apoptosis inhibitor	8
SUM52	HTRA4	HtrA serine peptidase 4	8
SUM52	ERLIN2	ER lipid raft associated 2	8
SUM52	BAG4	BCL2-associated athanogene 4	8
SUM52	LETM2	leucine zipper-EF-hand containing transmembrane protein 2	8
SUM52	STAR	steroidogenic acute regulatory protein	8
SUM52	ZMAT4	zinc finger, matrin type 4	8
SUM52	GOT1L1	glutamic-oxaloacetic transaminase 1-like 1	8
SUM52	RAB11FIP1	RAB11 family interacting protein 1 (class I)	8
SUM52	GPR124	G protein-coupled receptor 124	8
SUM52	ZNF703	zinc finger protein 703	8
SUM52	SFRP1	secreted frizzled-related protein 1	8
SUM52	ADRB3	adrenergic, beta-3-, receptor	8
SUM52	ADAM32	ADAM metallopeptidase domain 32	8
SUM52	PLEKHA2	pleckstrin homology domain containing, family A (phosphoinositide binding specific) member 2	8
SUM52	TACC1	transforming, acidic coiled-coil containing protein 1	8
SUM52	UNC5D	unc-5 homolog D (C. elegans)	8
SUM52	FGFR1	fibroblast growth factor receptor 1 (fms-related tyrosine kinase 2, Pfeiffer syndrome)	8
SUM52	ADAM18	ADAM metallopeptidase domain 18	8
SUM52	ADAM2	ADAM metallopeptidase domain 2 (fertilin beta)	8
SUM52	C8orf41	chromosome 8 open reading frame 41	8
SUM52	FUT10	fucosyltransferase 10 (alpha (1,3) fucosyltransferase)	8
SUM52	RBM13	RNA binding motif protein 13	8
SUM52	RNF122	ring finger protein 122	8
SUM52	FKSG2	apoptosis inhibitor	8
SUM52	WRN	Werner syndrome	8
SUM52	DUSP26	dual specificity phosphatase 26 (putative)	8
SUM52	NRG1	neuregulin 1	8
SUM52	UNC5D	unc-5 homolog D (C. elegans)	8
SUM52	LY6E	lymphocyte antigen 6 complex, locus E	8
SUM52	PYCRL	pyrroline-5-carboxylate reductase-like	8
SUM52	LYNX1	Ly6/neurotoxin 1	8
SUM52	PSCA	prostate stem cell antigen	8
SUM52	C8orf30A	chromosome 8 open reading frame 30A	8
SUM52	C8orf55	chromosome 8 open reading frame 55	8
SUM52	GPAA1	glycosylphosphatidylinositol anchor attachment protein 1 homolog (yeast)	8

SUM52	LY6K	lymphocyte antigen 6 complex, locus K	8
SUM52	GPT	glutamic-pyruvate transaminase (alanine aminotransferase)	8
SUM52	EPPK1	epiplakin 1	8
SUM52	PPP1R16A	protein phosphatase 1, regulatory (inhibitor) subunit 16A	8
SUM52	MAFA	v-maf musculoaponeurotic fibrosarcoma oncogene homolog A (avian)	8
SUM52	HSF1	heat shock transcription factor 1	8
SUM52	CYHR1	cysteine/histidine-rich 1	8
SUM52	TSTA3	tissue specific transplantation antigen P35B	8
SUM52	DGAT1	diacylglycerol O-acyltransferase homolog 1 (mouse)	8
SUM52	CPSF1	cleavage and polyadenylation specific factor 1, 160kDa	8
SUM52	EEF1D	eukaryotic translation elongation factor 1 delta (guanine nucleotide exchange protein)	8
SUM52	ZC3H3	zinc finger CCCH-type containing 3	8
SUM52	ZFP41	zinc finger protein 41 homolog (mouse)	8
SUM52	SLC39A4	solute carrier family 39 (zinc transporter), member 4	8
SUM52	MAF1	MAF1 homolog (S. cerevisiae)	8
SUM52	ZNF707	zinc finger protein 707	8
SUM52	JRK	jerky homolog (mouse)	8
SUM52	ARC	activity-regulated cytoskeleton-associated protein	8
SUM52	EXOSC4	exosome component 4	8
SUM52	VPS28	vacuolar protein sorting 28 homolog (S. cerevisiae)	8
SUM52	TIGD5	tigger transposable element derived 5	8
SUM52	C8orf31	chromosome 8 open reading frame 31	8
SUM52	CYC1	cytochrome c-1	8
SUM52	MAPK15	mitogen-activated protein kinase 15	8
SUM52	NFKBIL2	nuclear factor of kappa light polypeptide gene enhancer in B-cells inhibitor-like 2	8
SUM52	LY6H	lymphocyte antigen 6 complex, locus H	8
SUM52	LY6D	lymphocyte antigen 6 complex, locus D	8
SUM52	TOP1MT	topoisomerase (DNA) I, mitochondrial	8
SUM52	KIFC2	kinesin family member C2	8
SUM52	NAPRT1	nicotinate phosphoribosyltransferase domain containing 1	8
SUM52	GRINA	glutamate receptor, ionotropic, N-methyl D-asparate-associated protein 1 (glutamate binding)	8
SUM52	KIAA1833	hypothetical protein KIAA1833	8
SUM52	OPLAH	5-oxoprolinase (ATP-hydrolysing)	8
SUM52	PUF60	poly-U binding splicing factor 60KDa	8
SUM52	BOP1	block of proliferation 1	8
SUM52	SLURP1	secreted LY6/PLAUR domain containing 1	8
SUM52	RHPN1	rhophilin, Rho GTPase binding protein 1	8
SUM52	GPR172A	G protein-coupled receptor 172A	8
SUM52	CYP11B1	cytochrome P450, family 11, subfamily B, polypeptide 1	8
SUM52	FAM83H	family with sequence similarity 83, member H	8
SUM52	GML	GPI anchored molecule like protein	8

SUM52	PLEC1	plectin 1, intermediate filament binding protein 500kDa	8
SUM52	SCRT1	scratch homolog 1, zinc finger protein (Drosophila)	8
SUM52	LYPD2	LY6/PLAUR domain containing 2	8
SUM52	FBXL6	F-box and leucine-rich repeat protein 6	8
SUM52	ZNF696	zinc finger protein 696	8
SUM52	ZNF623	zinc finger protein 623	8
SUM52	CYP11B2	cytochrome P450, family 11, subfamily B, polypeptide 2	8
SUM52	NRBP2	nuclear receptor binding protein 2	8
SUM52	LOC338328	high density lipoprotein-binding protein	8
SUM52	ADCK5	aarF domain containing kinase 5	8
SUM52	SCRIB	scribbled homolog (Drosophila)	8
SUM52	PARP10	poly (ADP-ribose) polymerase family, member 10	8
SUM52	GSDMDC1	gasdermin domain containing 1	8
SUM52	FOXH1	forkhead box H1	8
SUM52	GLI4	GLI-Kruppel family member GLI4	8
SUM52	CEBPD	CCAAT/enhancer binding protein (C/EBP), delta	8
SUM52	MYST3	MYST histone acetyltransferase (monocytic leukemia) 3	8
SUM52	RNF170	ring finger protein 170	8
SUM52	VDAC3	voltage-dependent anion channel 3	8
SUM52	THAP1	THAP domain containing, apoptosis associated protein 1	8
SUM52	GOLGA7	golgi autoantigen, golgin subfamily a, 7	8
SUM52	IKBKB	inhibitor of kappa light polypeptide gene enhancer in B-cells, kinase beta	8
SUM52	GINS4	GINS complex subunit 4 (Sld5 homolog)	8
SUM52	SLC20A2	solute carrier family 20 (phosphate transporter), member 2	8
SUM52	AP3M2	adaptor-related protein complex 3, mu 2 subunit	8
SUM52	HGSNAT	heparan-alpha-glucosaminide N-acetyltransferase	8
SUM52	AGPAT6	1-acylglycerol-3-phosphate O-acyltransferase 6 (lysophosphatidic acid acyltransferase, zeta)	8
SUM52	PLAT	plasminogen activator, tissue	8
SUM52	POLB	polymerase (DNA directed), beta	8
SUM52	C8orf40	chromosome 8 open reading frame 40	8
SUM52	FNTA	farnesyltransferase, CAAX box, alpha	8
SUM52	ZMAT4	zinc finger, matrin type 4	8
SUM52	FLJ23356	hypothetical protein FLJ23356	8
SUM52	ANK1	ankyrin 1, erythrocytic	8
SUM52	HOOK3	hook homolog 3 (Drosophila)	8
SUM52	CHRNA6	cholinergic receptor, nicotinic, alpha 6	8
SUM52	CHRNB3	cholinergic receptor, nicotinic, beta 3	8
SUM52	SFRP1	secreted frizzled-related protein 1	8
SUM52	DKK4	dickkopf homolog 4 (Xenopus laevis)	8
SUM52	IKZF5	IKAROS family zinc finger 5 (Pegasus)	10
SUM52	C10orf88	chromosome 10 open reading frame 88	10
SUM52	PSTK	phosphoseryl-tRNA kinase	10
SUM52	ACADSB	acyl-Coenzyme A dehydrogenase, short/branched chain	10

SUM52	FAM24B	family with sequence similarity 24, member B	10
SUM52	CUZD1	CUB and zona pellucida-like domains 1	10
SUM52	FAM24A	family with sequence similarity 24, member A	10
SUM52	HMX2	H6 family homeobox 2	10
SUM52	DMBT1	deleted in malignant brain tumors 1	10
SUM52	NHLRC2	NHL repeat containing 2	10
SUM52	DCLRE1A	DNA cross-link repair 1A (PSO2 homolog, S. cerevisiae)	10
SUM52	CASP7	caspase 7, apoptosis-related cysteine peptidase	10
SUM52	C10orf81	chromosome 10 open reading frame 81	10
SUM52	C10orf118	chromosome 10 open reading frame 118	10
SUM52	NRAP	nebulin-related anchoring protein	10
SUM52	ADRB1	adrenergic, beta-1-, receptor	10
SUM52	ODZ4	odz, odd Oz/ten-m homolog 4 (Drosophila)	11
SUM52	PRKRIR	protein-kinase, interferon-inducible double stranded RNA dependent inhibitor, repressor of (P58 repressor)	11
SUM52	C11orf30	chromosome 11 open reading frame 30	11
SUM52	UVRAG	UV radiation resistance associated gene	11
SUM52	USP35	ubiquitin specific peptidase 35	11
SUM52	CLNS1A	chloride channel, nucleotide-sensitive, 1A	11
SUM52	ALG8	asparagine-linked glycosylation 8 homolog (S. cerevisiae, alpha-1,3-glucosyltransferase)	11
SUM52	RSF1	remodeling and spacing factor 1	11
SUM52	INTS4	integrator complex subunit 4	11
SUM52	PHCA	phytoceramidase, alkaline	11
SUM52	NARS2	asparaginyl-tRNA synthetase 2, mitochondrial (putative)	11
SUM52	THRSP	thyroid hormone responsive (SPOT14 homolog, rat)	11
SUM52	TSKU	tsukushin	11
SUM52	NDUFC2	NADH dehydrogenase (ubiquinone) 1, subcomplex unknown, 2, 14.5kDa	11
SUM52	RPS3	ribosomal protein S3	11
SUM52	C11orf67	chromosome 11 open reading frame 67	11
SUM52	PAK1	p21/Cdc42/Rac1-activated kinase 1 (STE20 homolog, yeast)	11
SUM52	GDPD5	glycerophosphodiester phosphodiesterase domain containing 5	11
SUM52	GAB2	GRB2-associated binding protein 2	11
SUM52	DGAT2	diacylglycerol O-acyltransferase homolog 2 (mouse)	11
SUM52	MOGAT2	monoacylglycerol O-acyltransferase 2	11
SUM52	ARRB1	arrestin, beta 1	11
SUM52	B3GNT6	UDP-GlcNAc:betaGal beta-1,3-N-acetylglucosaminyltransferase 6 (core 3 synthase)	11
SUM52	OMP	olfactory marker protein	11
SUM52	FLJ33790	hypothetical protein FLJ33790	11
SUM52	WNT11	wingless-type MMTV integration site family, member 11	11
SUM52	MYO7A	myosin VIIA	11
SUM52	AQP11	aquaporin 11	11

SUM52	GDPD4	glycerophosphodiester phosphodiesterase domain containing 4	11
SUM52	SERPINH1	serpin peptidase inhibitor, clade H (heat shock protein 47), member 1, (collagen binding protein 1)	11
SUM52	KCTD21	potassium channel tetramerisation domain containing 21	11
SUM52	MAP6	microtubule-associated protein 6	11
SUM52	LRRC32	leucine rich repeat containing 32	11
SUM52	MGC33846	hypothetical protein MGC33846	11
SUM52	CAPN5	calpain 5	11
SUM52	C12orf29	chromosome 12 open reading frame 29	12
SUM52	WDR51B	WD repeat domain 51B	12
SUM52	TMTC3	transmembrane and tetratricopeptide repeat containing 3	12
SUM52	CEP290	centrosomal protein 290kDa	12
SUM52	DUSP6	dual specificity phosphatase 6	12
SUM52	GALNT4	UDP-N-acetyl-alpha-D-galactosamine:polypeptide N-acetylgalactosaminyltransferase 4 (GalNAc-T4)	12
SUM52	KITLG	KIT ligand	12
SUM52	ATP2B1	ATPase, Ca++ transporting, plasma membrane 1	12
SUM52	MGAT4C	mannosyl (alpha-1,3-)-glycoprotein beta-1,4-N-acetylglucosaminyltransferase, isozyme C (putative)	12
SUM52	C12orf50	chromosome 12 open reading frame 50	12
SUM52	C12orf12	chromosome 12 open reading frame 12	12
SUM52	CCT2	chaperonin containing TCP1, subunit 2 (beta)	12
SUM52	MDM1	Mdm4, transformed 3T3 cell double minute 1, p53 binding protein (mouse)	12
SUM52	LGR5	leucine-rich repeat-containing G protein-coupled receptor 5	12
SUM52	SLC35E3	solute carrier family 35, member E3	12
SUM52	NUP107	nucleoporin 107kDa	12
SUM52	LEMD3	LEM domain containing 3	12
SUM52	CCDC131	coiled-coil domain containing 131	12
SUM52	YEATS4	YEATS domain containing 4	12
SUM52	FRS2	fibroblast growth factor receptor substrate 2	12
SUM52	CAND1	cullin-associated and neddylation-dissociated 1	12
SUM52	TMBIM4	transmembrane BAX inhibitor motif containing 4	12
SUM52	TBC1D15	TBC1 domain family, member 15	12
SUM52	LYZ	lysozyme (renal amyloidosis)	12
SUM52	RAB3IP	RAB3A interacting protein (rabin3)	12
SUM52	C12orf31	chromosome 12 open reading frame 31	12
SUM52	GLIPR1L2	GLI pathogenesis-related 1 like 2	12
SUM52	PHLDA1	pleckstrin homology-like domain, family A, member 1	12
SUM52	DYRK2	dual-specificity tyrosine-(Y)-phosphorylation regulated kinase 2	12
SUM52	RAP1B	RAP1B, member of RAS oncogene family	12
SUM52	CNOT2	CCR4-NOT transcription complex, subunit 2	12
SUM52	PTPRR	protein tyrosine phosphatase, receptor type, R	12
SUM52	MSRB3	methionine sulfoxide reductase B3	12

SUM52	RASSF3	Ras association (RalGDS/AF-6) domain family 3	12
SUM52	GNS	glucosamine (N-acetyl)-6-sulfatase (Sanfilippo disease IIID)	12
SUM52	CPSF6	cleavage and polyadenylation specific factor 6, 68kDa	12
SUM52	TMEM19	transmembrane protein 19	12
SUM52	TBK1	TANK-binding kinase 1	12
SUM52	KCNC2	potassium voltage-gated channel, Shaw-related subfamily, member 2	12
SUM52	RAB21	RAB21, member RAS oncogene family	12
SUM52	KCNMB4	potassium large conductance calcium-activated channel, subfamily M, beta member 4	12
SUM52	WIF1	WNT inhibitory factor 1	12
SUM52	IRAK3	interleukin-1 receptor-associated kinase 3	12
SUM52	IL26	interleukin 26	12
SUM52	CAPS2	calcyphosine 2	12
SUM52	CPM	carboxypeptidase M	12
SUM52	TPH2	tryptophan hydroxylase 2	12
SUM52	IFNG	interferon, gamma	12
SUM52	GLIPR1	GLI pathogenesis-related 1 (glioma)	12
SUM52	MDM2	Mdm2, transformed 3T3 cell double minute 2, p53 binding protein (mouse)	12
SUM52	XPOT	exportin, tRNA (nuclear export receptor for tRNAs)	12
SUM52	KRR1	KRR1, small subunit (SSU) processome component, homolog (yeast)	12
SUM52	IL22	interleukin 22	12
SUM52	HMGA2	high mobility group AT-hook 2	12
SUM52	THAP2	THAP domain containing, apoptosis associated protein 2	12
SUM52	HELB	helicase (DNA) B	12
SUM52	GLIPR1L1	GLI pathogenesis-related 1 like 1	12
SUM52	PTPRB	protein tyrosine phosphatase, receptor type, B	12
SUM52	BEST3	bestrophin 3	12
SUM52	TSPAN8	tetraspanin 8	12
SUM52	TRHDE	thyrotropin-releasing hormone degrading enzyme	12
SUM52	RNF111	ring finger protein 111	15
SUM52	ADAM10	ADAM metallopeptidase domain 10	15
SUM52	FAM63B	family with sequence similarity 63, member B	15
SUM52	SLTM	SAFB-like, transcription modulator	15
SUM52	CCNB2	cyclin B2	15
SUM52	LIPC	lipase, hepatic	15
SUM52	ALDH1A2	aldehyde dehydrogenase 1 family, member A2	15
SUM52	FAM81A	family with sequence similarity 81, member A	15
SUM52	AQP9	aquaporin 9	15
SUM52	MYO1E	myosin IE	15
SUM52	LDHAL6B	lactate dehydrogenase A-like 6B	15
SUM52	CCDC44	coiled-coil domain containing 44	17
SUM52	PSMC5	proteasome (prosome, macropain) 26S subunit, ATPase, 5	17

SUM52	FTSJ3	FtsJ homolog 3 (E. coli)	17
SUM52	TLK2	tousled-like kinase 2	17
SUM52	BCAS3	breast carcinoma amplified sequence 3	17
SUM52	CCDC47	coiled-coil domain containing 47	17
SUM52	POLG2	polymerase (DNA directed), gamma 2, accessory subunit	17
SUM52	DDX42	DEAD (Asp-Glu-Ala-Asp) box polypeptide 42	17
SUM52	SMARCD2	SWI/SNF related, matrix associated, actin dependent regulator of chromatin, subfamily d, member 2	17
SUM52	TEX2	testis expressed 2	17
SUM52	BRIP1	BRCA1 interacting protein C-terminal helicase 1	17
SUM52	CCDC45	coiled-coil domain containing 45	17
SUM52	WDR68	WD repeat domain 68	17
SUM52	INTS2	integrator complex subunit 2	17
SUM52	MED13	mediator complex subunit 13	17
SUM52	DDX5	DEAD (Asp-Glu-Ala-Asp) box polypeptide 5	17
SUM52	NACA2	nascent polypeptide-associated complex alpha subunit 2	17
SUM52	TBX2	T-box 2	17
SUM52	CYB561	cytochrome b-561	17
SUM52	MAP3K3	mitogen-activated protein kinase kinase kinase 3	17
SUM52	LIMD2	LIM domain containing 2	17
SUM52	LYK5	protein kinase LYK5	17
SUM52	ACE	angiotensin I converting enzyme (peptidyl-dipeptidase A) 1	17
SUM52	SMURF2	SMAD specific E3 ubiquitin protein ligase 2	17
SUM52	SCN4A	sodium channel, voltage-gated, type IV, alpha subunit	17
SUM52	GH1	growth hormone 1	17
SUM52	CD79B	CD79b molecule, immunoglobulin-associated beta	17
SUM52	PECAM1	platelet/endothelial cell adhesion molecule (CD31 antigen)	17
SUM52	KCNH6	potassium voltage-gated channel, subfamily H (eag-related), member 6	17
SUM52	GH2	growth hormone 2	17
SUM52	CSHL1	chorionic somatomammotropin hormone-like 1	17
SUM52	CSH2	chorionic somatomammotropin hormone 2	17
SUM52	CSH1	chorionic somatomammotropin hormone 1 (placental lactogen)	17
SUM52	MRC2	mannose receptor, C type 2	17
SUM52	LRRC37A3	leucine rich repeat containing 37, member A3	17
SUM52	TBX4	T-box 4	17
SUM52	ERN1	endoplasmic reticulum to nucleus signaling 1	17
SUM52	10-Mar	membrane-associated ring finger (C3HC4) 10	17
SUM52	ICAM2	intercellular adhesion molecule 2	17
SUM52	EFCAB3	EF-hand calcium binding domain 3	17
SUM52	ZNF813	zinc finger protein 813	19
SUM52	NDUFA3	NADH dehydrogenase (ubiquinone) 1 alpha subcomplex, 3, 9kDa	19
SUM52	ZNF331	zinc finger protein 331	19

SUM52	CACNG6	calcium channel, voltage-dependent, gamma subunit 6	19
SUM52	TFPT	TCF3 (E2A) fusion partner (in childhood Leukemia)	19
SUM52	CACNG8	calcium channel, voltage-dependent, gamma subunit 8	19
SUM52	PRPF31	PRP31 pre-mRNA processing factor 31 homolog (S. cerevisiae)	19
SUM52	PRKCG	protein kinase C, gamma	19
SUM52	OSCAR	osteoclast associated, immunoglobulin-like receptor	19
SUM52	MYADM	myeloid-associated differentiation marker	19
SUM52	CNOT3	CCR4-NOT transcription complex, subunit 3	19
SUM52	NLRP12	NLR family, pyrin domain containing 12	19
SUM52	BIRC8	baculoviral IAP repeat-containing 8	19
SUM52	CACNG7	calcium channel, voltage-dependent, gamma subunit 7	19
SUM52	ZNF180	zinc finger protein 180	19
SUM52	SYNGR4	synaptogyrin 4	19
SUM52	XRCC1	X-ray repair complementing defective repair in Chinese hamster cells 1	19
SUM52	TMEM145	transmembrane protein 145	19
SUM52	KDELR1	KDEL (Lys-Asp-Glu-Leu) endoplasmic reticulum protein retention receptor 1	19
SUM52	TRAPPC6A	trafficking protein particle complex 6A	19
SUM52	FLJ10781	hypothetical protein FLJ10781	19
SUM52	SNRPD2	small nuclear ribonucleoprotein D2 polypeptide 16.5kDa	19
SUM52	ZNF404	zinc finger protein 404	19
SUM52	ZNF233	zinc finger protein 233	19
SUM52	LIG1	ligase I, DNA, ATP-dependent	19
SUM52	AP2S1	adaptor-related protein complex 2, sigma 1 subunit	19
SUM52	CARD8	caspase recruitment domain family, member 8	19
SUM52	RPL18	ribosomal protein L18	19
SUM52	SAE1	SUMO1 activating enzyme subunit 1	19
SUM52	SLC8A2	solute carrier family 8 (sodium-calcium exchanger), member 2	19
SUM52	SFRS16	splicing factor, arginine/serine-rich 16	19
SUM52	ZNF226	zinc finger protein 226	19
SUM52	IRF2BP1	interferon regulatory factor 2 binding protein 1	19
SUM52	GEMIN7	gem (nuclear organelle) associated protein 7	19
SUM52	ZNF234	zinc finger protein 234	19
SUM52	DMWD	dystrophia myotonica, WD repeat containing	19
SUM52	CALM3	calmodulin 3 (phosphorylase kinase, delta)	19
SUM52	SEPW1	selenoprotein W, 1	19
SUM52	RUVBL2	RuvB-like 2 (E. coli)	19
SUM52	RTN2	reticulon 2	19
SUM52	LIN7B	lin-7 homolog B (C. elegans)	19
SUM52	GYS1	glycogen synthase 1 (muscle)	19
SUM52	FBXO46	F-box protein 46	19
SUM52	CLPTM1	cleft lip and palate associated transmembrane protein 1	19

SUM52	DMPK	dystrophia myotonica-protein kinase	19
SUM52	TMEM160	transmembrane protein 160	19
SUM52	ZNF225	zinc finger protein 225	19
SUM52	PPFIA3	protein tyrosine phosphatase, receptor type, f polypeptide (PTPRF), interacting protein (liprin), alpha 3	19
SUM52	EXOC3L2	exocyst complex component 3-like 2	19
SUM52	ZNF45	zinc finger protein 45	19
SUM52	PSCD2	pleckstrin homology, Sec7 and coiled-coil domains 2 (cytohesin-2)	19
SUM52	EMP3	epithelial membrane protein 3	19
SUM52	CNFN	cornifelin	19
SUM52	ZNF224	zinc finger protein 224	19
SUM52	PPP1R15A	protein phosphatase 1, regulatory (inhibitor) subunit 15A	19
SUM52	PLA2G4C	phospholipase A2, group IVC (cytosolic, calcium-independent)	19
SUM52	SYMPK	symplekin	19
SUM52	CCDC9	coiled-coil domain containing 9	19
SUM52	STRN4	striatin, calmodulin binding protein 4	19
SUM52	KIAA1183	KIAA1183 protein	19
SUM52	ERCC1	excision repair cross-complementing rodent repair deficiency, complementation group 1 (includes overlapping antisense sequence)	19
SUM52	ZNF223	zinc finger protein 223	19
SUM52	NUCB1	nucleobindin 1	19
SUM52	LYPD5	LY6/PLAUR domain containing 5	19
SUM52	CCDC8	coiled-coil domain containing 8	19
SUM52	PVRL2	poliovirus receptor-related 2 (herpesvirus entry mediator B)	19
SUM52	GLTSCR2	glioma tumor suppressor candidate region gene 2	19
SUM52	CIC	capicua homolog (Drosophila)	19
SUM52	ZNF284	zinc finger protein 284	19
SUM52	KCNJ14	potassium inwardly-rectifying channel, subfamily J, member 14	19
SUM52	ZNF575	zinc finger protein 575	19
SUM52	ZNF230	zinc finger protein 230	19
SUM52	ZNF227	zinc finger protein 227	19
SUM52	APOC1	apolipoprotein C-I	19
SUM52	BCL3	B-cell CLL/lymphoma 3	19
SUM52	DBP	D site of albumin promoter (albumin D-box) binding protein	19
SUM52	FTL	ferritin, light polypeptide	19
SUM52	GRWD1	glutamate-rich WD repeat containing 1	19
SUM52	SULT2A1	sulfotransferase family, cytosolic, 2A, dehydroepiandrosterone (DHEA)-preferring, member 1	19
SUM52	ZNF235	zinc finger protein 235	19
SUM52	CCDC114	coiled-coil domain containing 114	19
SUM52	EML2	echinoderm microtubule associated protein like 2	19
SUM52	LYPD3	LY6/PLAUR domain containing 3	19

SUM52	ZNF155	zinc finger protein 155	19
SUM52	CGB1	chorionic gonadotropin, beta polypeptide 1	19
SUM52	HRC	histidine rich calcium binding protein	19
SUM52	FOXA3	forkhead box A3	19
SUM52	SULT2B1	sulfotransferase family, cytosolic, 2B, member 1	19
SUM52	APOC2	apolipoprotein C-II	19
SUM52	FAM83E	family with sequence similarity 83, member E	19
SUM52	ELSPBP1	epididymal sperm binding protein 1	19
SUM52	PSG1	pregnancy specific beta-1-glycoprotein 1	19
SUM52	PAFAH1B3	platelet-activating factor acetylhydrolase, isoform Ib, gamma subunit 29kDa	19
SUM52	LOC126147	hypothetical protein BC018697	19
SUM52	CGB	chorionic gonadotropin, beta polypeptide	19
SUM52	GPR77	G protein-coupled receptor 77	19
SUM52	C19orf7	chromosome 19 open reading frame 7	19
SUM52	QPCTL	glutaminyl-peptide cyclotransferase-like	19
SUM52	RELB	v-rel reticuloendotheliosis viral oncogene homolog B, nuclear factor of kappa light polypeptide gene enhancer in B-cells 3 (avian)	19
SUM52	GRLF1	glucocorticoid receptor DNA binding factor 1	19
SUM52	NTF5	neurotrophin 5 (neurotrophin 4/5)	19
SUM52	FGF21	fibroblast growth factor 21	19
SUM52	ZNF576	zinc finger protein 576	19
SUM52	NAPA	N-ethylmaleimide-sensitive factor attachment protein, alpha	19
SUM52	C19orf61	chromosome 19 open reading frame 61	19
SUM52	PPP1R13L	protein phosphatase 1, regulatory (inhibitor) subunit 13 like	19
SUM52	DACT3	dapper, antagonist of beta-catenin, homolog 3 (Xenopus laevis)	19
SUM52	TMEM143	transmembrane protein 143	19
SUM52	PPP5C	protein phosphatase 5, catalytic subunit	19
SUM52	SLC1A5	solute carrier family 1 (neutral amino acid transporter), member 5	19
SUM52	DHX34	DEAH (Asp-Glu-Ala-His) box polypeptide 34	19
SUM52	RASIP1	Ras interacting protein 1	19
SUM52	PGLYRP1	peptidoglycan recognition protein 1	19
SUM52	FOSB	FBJ murine osteosarcoma viral oncogene homolog B	19
SUM52	C5AR1	complement component 5a receptor 1	19
SUM52	PSG11	pregnancy specific beta-1-glycoprotein 11	19
SUM52	NOVA2	neuro-oncological ventral antigen 2	19
SUM52	GIPR	gastric inhibitory polypeptide receptor	19
SUM52	MEIS3	Meis homeobox 3	19
SUM52	TOMM40	translocase of outer mitochondrial membrane 40 homolog (yeast)	19
SUM52	FLJ10490	hypothetical protein FLJ10490	19
SUM52	FUT1	fucosyltransferase 1 (galactoside 2-alpha-L-fucosyltransferase, H blood group)	19

SUM52	CEACAM8	carcinoembryonic antigen-related cell adhesion molecule 8	19
SUM52	CRX	cone-rod homeobox	19
SUM52	BAX	BCL2-associated X protein	19
SUM52	CKM	creatine kinase, muscle	19
SUM52	MARK4	MAP/microtubule affinity-regulating kinase 4	19
SUM52	ETHE1	ethylmalonic encephalopathy 1	19
SUM52	ZNF228	zinc finger protein 228	19
SUM52	ZNF342	zinc finger protein 342	19
SUM52	GRIN2D	glutamate receptor, ionotropic, N-methyl D-aspartate 2D	19
SUM52	CEACAM1	carcinoembryonic antigen-related cell adhesion molecule 1 (biliary glycoprotein)	19
SUM52	PLAUR	plasminogen activator, urokinase receptor	19
SUM52	KCNA7	potassium voltage-gated channel, shaker-related subfamily, member 7	19
SUM52	EHD2	EH-domain containing 2	19
SUM52	SLC6A16	solute carrier family 6, member 16	19
SUM52	ERCC2	excision repair cross-complementing rodent repair deficiency, complementation group 2 (xeroderma pigmentosum D)	19
SUM52	SPACA4	sperm acrosome associated 4	19
SUM52	APOE	apolipoprotein E	19
SUM52	TEX101	testis expressed 101	19
SUM52	NPAS1	neuronal PAS domain protein 1	19
SUM52	CD3EAP	CD3e molecule, epsilon associated protein	19
SUM52	NKPD1	NTPase, KAP family P-loop domain containing 1	19
SUM52	GNG8	guanine nucleotide binding protein (G protein), gamma 8	19
SUM52	PLEKHA4	pleckstrin homology domain containing, family A (phosphoinositide binding specific) member 4	19
SUM52	CADM4	cell adhesion molecule 4	19
SUM52	BCAM	basal cell adhesion molecule (Lutheran blood group)	19
SUM52	TRPM4	transient receptor potential cation channel, subfamily M, member 4	19
SUM52	CABP5	calcium binding protein 5	19
SUM52	PTGIR	prostaglandin I2 (prostacyclin) receptor (IP)	19
SUM52	LHB	luteinizing hormone beta polypeptide	19
SUM52	CGB2	chorionic gonadotropin, beta polypeptide 2	19
SUM52	APOC4	apolipoprotein C-IV	19
SUM52	KLC3	kinesin light chain 3	19
SUM52	GLTSCR1	glioma tumor suppressor candidate region gene 1	19
SUM52	RSHL1	radial spokehead-like 1	19
SUM52	ZNF114	zinc finger protein 114	19
SUM52	LIPE	lipase, hormone-sensitive	19
SUM52	HIF3A	hypoxia inducible factor 3, alpha subunit	19
SUM52	PSG8	pregnancy specific beta-1-glycoprotein 8	19
SUM52	ZNF428	zinc finger protein 428	19
SUM52	SIX5	SIX homeobox 5	19

SUM52	CBLC	Cas-Br-M (murine) ecotropic retroviral transforming sequence c	19
SUM52	MEGF8	multiple EGF-like-domains 8	19
SUM52	BBC3	BCL2 binding component 3	19
SUM52	FUT2	fucosyltransferase 2 (secretor status included)	19
SUM52	VASP	vasodilator-stimulated phosphoprotein	19
SUM52	UNQ473	DMC	19
SUM52	PRKD2	protein kinase D2	19
SUM52	OPA3	optic atrophy 3 (autosomal recessive, with chorea and spastic paraplegia)	19
SUM52	DHDH	dihydrodiol dehydrogenase (dimeric)	19
SUM52	PSG6	pregnancy specific beta-1-glycoprotein 6	19
SUM52	ZNF283	zinc finger protein 283	19
SUM52	IRGC	immunity-related GTPase family, cinema	19
SUM52	LOC339344	hypothetical protein LOC339344	19
SUM52	PSG5	pregnancy specific beta-1-glycoprotein 5	19
SUM52	ZNF221	zinc finger protein 221	19
SUM52	TPRX1	tetra-peptide repeat homeobox 1	19
SUM52	CA11	carbonic anhydrase XI	19
SUM52	CEACAM19	carcinoembryonic antigen-related cell adhesion molecule 19	19
SUM52	PVR	poliovirus receptor	19
SUM52	IGFL2	IGF-like family member 2	19
SUM52	KCNN4	potassium intermediate/small conductance calcium-activated channel, subfamily N, member 4	19
SUM52	KPTN	kaptin (actin binding protein)	19
SUM52	PSG9	pregnancy specific beta-1-glycoprotein 9	19
SUM52	CD177	CD177 molecule	19
SUM52	SNRP70	small nuclear ribonucleoprotein 70kDa polypeptide (RNP antigen)	19
SUM52	BCAT2	branched chain aminotransferase 2, mitochondrial	19
SUM52	GPR4	G protein-coupled receptor 4	19
SUM52	HSD17B14	hydroxysteroid (17-beta) dehydrogenase 14	19
SUM52	TULP2	tubby like protein 2	19
SUM52	IZUMO1	izumo sperm-egg fusion 1	19
SUM52	FKRP	fukutin related protein	19
SUM52	FLJ36070	likely ortholog of MEF2-activating SAP transcriptional regulator	19
SUM52	PSG3	pregnancy specific beta-1-glycoprotein 3	19
SUM52	DNAJB1	DnaJ (Hsp40) homolog, subfamily B, member 1	19
SUM52	NDUFB7	NADH dehydrogenase (ubiquinone) 1 beta subcomplex, 7, 18kDa	19
SUM52	EMR2	egf-like module containing, mucin-like, hormone receptor-like 2	19
SUM52	PTGER1	prostaglandin E receptor 1 (subtype EP1), 42kDa	19
SUM52	EMR3	egf-like module containing, mucin-like, hormone receptor-like 3	19

SUM52	GIPC1	GIPC PDZ domain containing family, member 1	19
SUM52	DDX39	DEAD (Asp-Glu-Ala-Asp) box polypeptide 39	19
SUM52	ZNF333	zinc finger protein 333	19
SUM52	GPSN2	glycoprotein, synaptic 2	19
SUM52	PKN1	protein kinase N1	19
SUM52	NCR1	natural cytotoxicity triggering receptor 1	19
SUM52	SYT5	synaptotagmin V	19
SUM52	TNNI3	troponin I type 3 (cardiac)	19
SUM52	EPS8L1	EPS8-like 1	19
SUM52	FCAR	Fc fragment of IgA, receptor for	19
SUM52	ISOC2	isochorismatase domain containing 2	19
SUM52	TMC4	transmembrane channel-like 4	19
SUM52	TNNT1	troponin T type 1 (skeletal, slow)	19
SUM52	TSEN34	tRNA splicing endonuclease 34 homolog (S. cerevisiae)	19
SUM52	TTYH1	tweety homolog 1 (Drosophila)	19
SUM52	ZNF444	zinc finger protein 444	19
SUM52	LENG4	leukocyte receptor cluster (LRC) member 4	19
SUM52	ZNF579	zinc finger protein 579	19
SUM52	NLRP7	NLR family, pyrin domain containing 7	19
SUM52	HSPBP1	hsp70-interacting protein	19
SUM52	ZNF784	zinc finger protein 784	19
SUM52	RPS9	ribosomal protein S9	19
SUM52	LAIR1	leukocyte-associated immunoglobulin-like receptor 1	19
SUM52	CDC42EP5	CDC42 effector protein (Rho GTPase binding) 5	19
SUM52	NLRP2	NLR family, pyrin domain containing 2	19
SUM52	LENG9	leukocyte receptor cluster (LRC) member 9	19
SUM52	TMEM86B	transmembrane protein 86B	19
SUM52	PPP1R12C	protein phosphatase 1, regulatory (inhibitor) subunit 12C	19
SUM52	ZNF524	zinc finger protein 524	19
SUM52	PTPRH	protein tyrosine phosphatase, receptor type, H	19
SUM52	LILRB3	leukocyte immunoglobulin-like receptor, subfamily B (with TM and ITIM domains), member 3	19
SUM52	CNOT3	CCR4-NOT transcription complex, subunit 3	19
SUM52	LILRB4	leukocyte immunoglobulin-like receptor, subfamily B (with TM and ITIM domains), member 4	19
SUM52	TMEM190	transmembrane protein 190	19
SUM52	RDH13	retinol dehydrogenase 13 (all-trans/9-cis)	19
SUM52	UBE2S	ubiquitin-conjugating enzyme E2S	19
SUM52	ZNF580	zinc finger protein 580	19
SUM52	LILRB5	leukocyte immunoglobulin-like receptor, subfamily B (with TM and ITIM domains), member 5	19
SUM52	IL11	interleukin 11	19
SUM52	SAPS1	SAPS domain family, member 1	19
SUM52	KIR2DL1	killer cell immunoglobulin-like receptor, two domains, long cytoplasmic tail, 1	19

SUM52	LILRA4	leukocyte immunoglobulin-like receptor, subfamily A (with TM domain), member 4	19
SUM52	U2AF2	U2 small nuclear RNA auxiliary factor 2	19
SUM52	NAT14	N-acetyltransferase 14	19
SUM52	ZNF581	zinc finger protein 581	19
SUM52	RPL28	ribosomal protein L28	19
SUM52	KIR3DL3	killer cell immunoglobulin-like receptor, three domains, long cytoplasmic tail, 3	19
SUM52	KIR2DL4	killer cell immunoglobulin-like receptor, two domains, long cytoplasmic tail, 4	19
SUM52	COX6B2	cytochrome c oxidase subunit VIb polypeptide 2 (testis)	19
SUM52	KIR3DL1	killer cell immunoglobulin-like receptor, three domains, long cytoplasmic tail, 1	19
SUM52	GP6	glycoprotein VI (platelet)	19
SUM52	ZNF787	zinc finger protein 787	19
SUM52	SUV420H2	suppressor of variegation 4-20 homolog 2 (Drosophila)	19
SUM52	NLRP11	NLR family, pyrin domain containing 11	19
SUM52	ZSCAN5	zinc finger and SCAN domain containing 5	19
SUM52	LENG8	leukocyte receptor cluster (LRC) member 8	19
SUM52	ZNF582	zinc finger protein 582	19
SUM52	CCDC106	coiled-coil domain containing 106	19
SUM52	LILRB1	leukocyte immunoglobulin-like receptor, subfamily B (with TM and ITIM domains), member 1	19
SUM52	LENG1	leukocyte receptor cluster (LRC) member 1	19
SUM52	ZNF628	zinc finger protein 628	19
SUM52	LILRB2	leukocyte immunoglobulin-like receptor, subfamily B (with TM and ITIM domains), member 2	19
SUM52	LILRA3	leukocyte immunoglobulin-like receptor, subfamily A (without TM domain), member 3	19
SUM52	EPN1	epsin 1	19
SUM52	NLRP4	NLR family, pyrin domain containing 4	19
SUM52	LAIR2	leukocyte-associated immunoglobulin-like receptor 2	19
SUM52	FIZ1	FLT3-interacting zinc finger 1	19
SUM52	BRSK1	BR serine/threonine kinase 1	19
SUM52	NLRP5	NLR family, pyrin domain containing 5	19
SUM52	NLRP9	NLR family, pyrin domain containing 9	19
SUM52	NLRP13	NLR family, pyrin domain containing 13	19
SUM52	NLRP8	NLR family, pyrin domain containing 8	19
SUM52	GALP	galanin-like peptide precursor	19
SUM52	ZNF137	zinc finger protein 137	19
SUM52	ZNF813	zinc finger protein 813	19
SUM52	ZNF665	zinc finger protein 665	19
SUM52	ZNF415	zinc finger protein 415	19
SUM52	ZNF347	zinc finger protein 347	19
SUM52	ZNF160	zinc finger protein 160	19
SUM52	ZNF610	zinc finger protein 610	19

SUM52	ZNF600	zinc finger protein 600	19
SUM52	ZNF320	zinc finger protein 320	19
SUM52	ZNF83	zinc finger protein 83	19
SUM52	ZNF528	zinc finger protein 528	19
SUM52	ZNF28	zinc finger protein 28	19
SUM52	ZNF468	zinc finger protein 468	19
SUM52	ZNF808	zinc finger protein 808	19
SUM52	ZNF480	zinc finger protein 480	19
SUM52	BIRC8	baculoviral IAP repeat-containing 8	19
SUM52	VN1R2	vomeronasal 1 receptor 2	19
SUM52	VN1R4	vomeronasal 1 receptor 4	19
SUM52	PEG3	paternally expressed 3	19
SUM52	ZNF304	zinc finger protein 304	19
SUM52	ZNF264	zinc finger protein 264	19
SUM52	ZNF17	zinc finger protein 17	19
SUM52	ZIM3	zinc finger, imprinted 3	19
SUM52	ZNF543	zinc finger protein 543	19
SUM52	AURKC	aurora kinase C	19
SUM52	BC37295_3	hypothetical BC37295_3	19
SUM52	USP29	ubiquitin specific peptidase 29	19
SUM52	CABLES2	Cdk5 and Abl enzyme substrate 2	20
SUM52	ADRM1	adhesion regulating molecule 1	20
SUM52	OSBPL2	oxysterol binding protein-like 2	20
SUM52	TAF4	TAF4 RNA polymerase II, TATA box binding protein (TBP)-associated factor, 135kDa	20
SUM52	SS18L1	synovial sarcoma translocation gene on chromosome 18-like 1	20
SUM52	LAMA5	laminin, alpha 5	20
SUM52	RPS21	ribosomal protein S21	20
SUM52	PSMA7	proteasome (prosome, macropain) subunit, alpha type, 7	20
SUM52	LSM14B	LSM14B, SCD6 homolog B (S. cerevisiae)	20
SUM52	GTPBP5	GTP binding protein 5 (putative)	20
SUM52	HRH3	histamine receptor H3	20
SUM52	C20orf151	chromosome 20 open reading frame 151	20
SUM52	GATA5	GATA binding protein 5	20
SUM52	SERINC3	serine incorporator 3	20
SUM52	ADA	adenosine deaminase	20
SUM52	YWHAB	tyrosine 3-monooxygenase/tryptophan 5-monooxygenase activation protein, beta polypeptide	20
SUM52	C20orf121	chromosome 20 open reading frame 121	20
SUM52	STK4	serine/threonine kinase 4	20
SUM52	SLPI	secretory leukocyte peptidase inhibitor	20
SUM52	WISP2	WNT1 inducible signaling pathway protein 2	20
SUM52	TOMM34	translocase of outer mitochondrial membrane 34	20

SUM52	KCNS1	potassium voltage-gated channel, delayed-rectifier, subfamily S, member 1	20
SUM52	KCNK15	potassium channel, subfamily K, member 15	20
SUM52	RIMS4	regulating synaptic membrane exocytosis 4	20
SUM52	MATN4	matrilin 4	20
SUM52	PKIG	protein kinase (cAMP-dependent, catalytic) inhibitor gamma	20
SUM52	SEMG2	semenogelin II	20
SUM52	PI3	peptidase inhibitor 3, skin-derived (SKALP)	20
SUM52	RBPJL	recombination signal binding protein for immunoglobulin kappa J region-like	20
SUM52	SEMG1	semenogelin I	20
SUM52	SDC4	syndecan 4	20
SUM52	WFDC12	WAP four-disulfide core domain 12	20
SUM52	WFDC5	WAP four-disulfide core domain 5	20
SUM52	DIDO1	death inducer-obliterator 1	20
SUM52	ARFGAP1	ADP-ribosylation factor GTPase activating protein 1	20
SUM52	STMN3	stathmin-like 3	20
SUM52	PCMTD2	protein-L-isoaspartate (D-aspartate) O-methyltransferase domain containing 2	20
SUM52	SRMS	src-related kinase lacking C-terminal regulatory tyrosine and N-terminal myristylation sites	20
SUM52	GATA5	GATA binding protein 5	20
SUM52	C20orf195	chromosome 20 open reading frame 195	20
SUM52	TCEA2	transcription elongation factor A (SII), 2	20
SUM52	YTHDF1	YTH domain family, member 1	20
SUM52	CHRNA4	cholinergic receptor, nicotinic, alpha 4	20
SUM52	C20orf11	chromosome 20 open reading frame 11	20
SUM52	ZNF512B	zinc finger protein 512B	20
SUM52	TNFRSF6B	tumor necrosis factor receptor superfamily, member 6b, decoy	20
SUM52	PRPF6	PRP6 pre-mRNA processing factor 6 homolog (S. cerevisiae)	20
SUM52	EEF1A2	eukaryotic translation elongation factor 1 alpha 2	20
SUM52	BHLHB4	basic helix-loop-helix domain containing, class B, 4	20
SUM52	C20orf149	chromosome 20 open reading frame 149	20
SUM52	C20orf135	chromosome 20 open reading frame 135	20
SUM52	COL9A3	collagen, type IX, alpha 3	20
SUM52	ARFRP1	ADP-ribosylation factor related protein 1	20
SUM52	SOX18	SRY (sex determining region Y)-box 18	20
SUM52	SLC2A4RG	SLC2A4 regulator	20
SUM52	GMEB2	glucocorticoid modulatory element binding protein 2	20
SUM52	OGFR	opioid growth factor receptor	20
SUM52	MYT1	myelin transcription factor 1	20
SUM52	LOC198437	bA299N6.3	20
SUM52	KCNQ2	potassium voltage-gated channel, KQT-like subfamily, member 2	20
SUM52	SAMD10	sterile alpha motif domain containing 10	20

SUM52	PRR17	proline rich 17	20
SUM52	ZBTB46	zinc finger and BTB domain containing 46	20
SUM52	C20orf58	chromosome 20 open reading frame 58	20
SUM52	C20orf59	chromosome 20 open reading frame 59	20
SUM52	PTK6	PTK6 protein tyrosine kinase 6	20
SUM52	NPBWR2	neuropeptides B/W receptor 2	20
SUM52	UCKL1	uridine-cytidine kinase 1-like 1	20
SUM52	RGS19	regulator of G-protein signaling 19	20
SUM52	OPRL1	opiate receptor-like 1	20
SUM52	PRIC285	peroxisomal proliferator-activated receptor A interacting complex 285	20
SUM52	C20orf20	chromosome 20 open reading frame 20	20
SUM52	BIRC7	baculoviral IAP repeat-containing 7 (livin)	20
SUM52	TCFL5	transcription factor-like 5 (basic helix-loop-helix)	20
SUM52	C20orf200	chromosome 20 open reading frame 200	20
SUM52	LIME1	Lck interacting transmembrane adaptor 1	20
SUM52	NTSR1	neurotensin receptor 1 (high affinity)	20
SUM52	TPD52L2	tumor protein D52-like 2	20
SUM52	COL20A1	collagen, type XX, alpha 1	20
SUM52	SLCO4A1	solute carrier organic anion transporter family, member 4A1	20
SUM52	C20orf166	chromosome 20 open reading frame 166	20
SUM52	NPEPL1	aminopeptidase-like 1	20
SUM52	TFAP2C	transcription factor AP-2 gamma (activating enhancer binding protein 2 gamma)	20
SUM52	C20orf106	chromosome 20 open reading frame 106	20
SUM52	TH1L	TH1-like (Drosophila)	20
SUM52	ATP5E	ATP synthase, H+ transporting, mitochondrial F1 complex, epsilon subunit	20
SUM52	SYCP2	synaptonemal complex protein 2	20
SUM52	RAB22A	RAB22A, member RAS oncogene family	20
SUM52	APCDD1L	adenomatosis polyposis coli down-regulated 1-like	20
SUM52	C20orf177	chromosome 20 open reading frame 177	20
SUM52	TAF4	TAF4 RNA polymerase II, TATA box binding protein (TBP)-associated factor, 135kDa	20
SUM52	BMP7	bone morphogenetic protein 7 (osteogenic protein 1)	20
SUM52	TMEPAI	transmembrane, prostate androgen induced RNA	20
SUM52	HMG1L1	high-mobility group (nonhistone chromosomal) protein 1-like 1	20
SUM52	GNAS	GNAS complex locus	20
SUM52	CDH26	cadherin-like 26	20
SUM52	PPP1R3D	protein phosphatase 1, regulatory (inhibitor) subunit 3D	20
SUM52	VAPB	VAMP (vesicle-associated membrane protein)-associated protein B and C	20
SUM52	CTCFL	CCCTC-binding factor (zinc finger protein)-like	20
SUM52	CTSZ	cathepsin Z	20
SUM52	PCK1	phosphoenolpyruvate carboxykinase 1 (soluble)	20

SUM52	C20orf43	chromosome 20 open reading frame 43	20
SUM52	LSM14B	LSM14B, SCD6 homolog B (S. cerevisiae)	20
SUM52	RBM38	RNA binding motif protein 38	20
SUM52	STX16	syntaxin 16	20
SUM52	SLMO2	slowmo homolog 2 (Drosophila)	20
SUM52	TUBB1	tubulin, beta 1	20
SUM52	CDH4	cadherin 4, type 1, R-cadherin (retinal)	20
SUM52	RAE1	RAE1 RNA export 1 homolog (S. pombe)	20
SUM52	C20orf197	chromosome 20 open reading frame 197	20
SUM52	PHACTR3	phosphatase and actin regulator 3	20
SUM52	C20orf85	chromosome 20 open reading frame 85	20
SUM52	SPO11	SPO11 meiotic protein covalently bound to DSB homolog (S. cerevisiae)	20
SUM52	ZBP1	Z-DNA binding protein 1	20
SUM52	EDN3	endothelin 3	20
SUM52	SNX21	sorting nexin family member 21	20
SUM52	CD40	CD40 molecule, TNF receptor superfamily member 5	20
SUM52	PTGIS	prostaglandin I2 (prostacyclin) synthase	20
SUM52	SLC12A5	solute carrier family 12, (potassium-chloride transporter) member 5	20
SUM52	KCNG1	potassium voltage-gated channel, subfamily G, member 1	20
SUM52	ZNF334	zinc finger protein 334	20
SUM52	MMP9	matrix metallopeptidase 9 (gelatinase B, 92kDa gelatinase, 92kDa type IV collagenase)	20
SUM52	ACOT8	acyl-CoA thioesterase 8	20
SUM52	UBE2V1	ubiquitin-conjugating enzyme E2 variant 1	20
SUM52	STAU1	staufen, RNA binding protein, homolog 1 (Drosophila)	20
SUM52	ARFGEF2	ADP-ribosylation factor guanine nucleotide-exchange factor 2 (brefeldin A-inhibited)	20
SUM52	ZNF313	zinc finger protein 313	20
SUM52	ZSWIM1	zinc finger, SWIM-type containing 1	20
SUM52	ZSWIM3	zinc finger, SWIM-type containing 3	20
SUM52	TP53RK	TP53 regulating kinase	20
SUM52	ELMO2	engulfment and cell motility 2	20
SUM52	DNTTIP1	deoxynucleotidyltransferase, terminal, interacting protein 1	20
SUM52	DBNDD2	dysbindin (dystrobrevin binding protein 1) domain containing 2	20
SUM52	SLC35C2	solute carrier family 35, member C2	20
SUM52	SLC13A3	solute carrier family 13 (sodium-dependent dicarboxylate transporter), member 3	20
SUM52	ADNP	activity-dependent neuroprotector homeobox	20
SUM52	CTSA	cathepsin A	20
SUM52	KCNB1	potassium voltage-gated channel, Shab-related subfamily, member 1	20
SUM52	NEURL2	neuralized homolog 2 (Drosophila)	20

SUM52	B4GALT5	UDP-Gal:betaGlcNAc beta 1,4- galactosyltransferase, polypeptide 5	20
SUM52	CSE1L	CSE1 chromosome segregation 1-like (yeast)	20
SUM52	ZNF335	zinc finger protein 335	20
SUM52	WFDC3	WAP four-disulfide core domain 3	20
SUM52	BCAS4	breast carcinoma amplified sequence 4	20
SUM52	PREX1	phosphatidylinositol 3,4,5-trisphosphate-dependent RAC exchanger 1	20
SUM52	PIGT	phosphatidylinositol glycan anchor biosynthesis, class T	20
SUM52	C20orf175	chromosome 20 open reading frame 175	20
SUM52	SLC9A8	solute carrier family 9 (sodium/hydrogen exchanger), member 8	20
SUM52	SNAI1	snail homolog 1 (Drosophila)	20
SUM52	CEBPB	CCAAT/enhancer binding protein (C/EBP), beta	20
SUM52	NFATC2	nuclear factor of activated T-cells, cytoplasmic, calcineurin-dependent 2	20
SUM52	SLC2A10	solute carrier family 2 (facilitated glucose transporter), member 10	20
SUM52	C20orf10	chromosome 20 open reading frame 10	20
SUM52	UBE2C	ubiquitin-conjugating enzyme E2C	20
SUM52	DDX27	DEAD (Asp-Glu-Ala-Asp) box polypeptide 27	20
SUM52	MOCS3	molybdenum cofactor synthesis 3	20
SUM52	WFDC8	WAP four-disulfide core domain 8	20
SUM52	WFDC13	WAP four-disulfide core domain 13	20
SUM52	ZNFX1	zinc finger, NFX1-type containing 1	20
SUM52	SPATA2	spermatogenesis associated 2	20
SUM52	DPM1	dolichyl-phosphate mannosyltransferase polypeptide 1, catalytic subunit	20
SUM52	NCOA3	nuclear receptor coactivator 3	20
SUM52	WFDC9	WAP four-disulfide core domain 9	20
SUM52	PTPN1	protein tyrosine phosphatase, non-receptor type 1	20
SUM52	WFDC6	WAP four-disulfide core domain 6	20
SUM52	C20orf67	chromosome 20 open reading frame 67	20
SUM52	WFDC10B	WAP four-disulfide core domain 10B	20
SUM52	PARD6B	par-6 partitioning defective 6 homolog beta (C. elegans)	20
SUM52	WFDC11	WAP four-disulfide core domain 11	20
SUM52	C20orf165	chromosome 20 open reading frame 165	20
SUM52	PLTP	phospholipid transfer protein	20
SUM52	NCOA5	nuclear receptor coactivator 5	20
SUM52	SALL4	sal-like 4 (Drosophila)	20
SUM52	ZFP64	zinc finger protein 64 homolog (mouse)	20
SUM52	ATP9A	ATPase, Class II, type 9A	20
SUM52	TNNC2	troponin C type 2 (fast)	20
SUM52	CDH22	cadherin-like 22	20
SUM52	RBPJL	recombination signal binding protein for immunoglobulin kappa J region-like	20

SUM52	SULF2	sulfatase 2	20
SUM52	ZMYND8	zinc finger, MYND-type containing 8	20
SUM52	SDC4	syndecan 4	20
SUM52	EYA2	eyes absent homolog 2 (Drosophila)	20
SUM52	SPINLW1	serine peptidase inhibitor-like, with Kunitz and WAP domains 1 (eppin)	20
SUM52	WFDC10A	WAP four-disulfide core domain 10A	20
SUM52	POLA1	polymerase (DNA directed), alpha 1	X
SUM52	MAGEB6	melanoma antigen family B, 6	X
SUM52	ARX	aristaless related homeobox	X
SUM52	MAGEB18	melanoma antigen family B, 18	X
SUM52	MAGEB10	melanoma antigen family B, 10	X
SUM52	GEMIN8	gem (nuclear organelle) associated protein 8	X
SUM52	OFD1	oral-facial-digital syndrome 1	X
SUM52	FANCB	Fanconi anemia, complementation group B	X
SUM52	PRPS2	phosphoribosyl pyrophosphate synthetase 2	X
SUM52	MOSPD2	motile sperm domain containing 2	X
SUM52	TMEM27	transmembrane protein 27	X
SUM52	GPM6B	glycoprotein M6B	X
SUM52	TRAPPC2	trafficking protein particle complex 2	X
SUM52	ACE2	angiotensin I converting enzyme (peptidyl-dipeptidase A) 2	X
SUM52	TMSB4X	thymosin, beta 4, X-linked	X
SUM52	PIR	pirin (iron-binding nuclear protein)	X
SUM52	BMX	BMX non-receptor tyrosine kinase	X
SUM52	PIGA	phosphatidylinositol glycan anchor biosynthesis, class A (paroxysmal nocturnal hemoglobinuria)	X
SUM52	FRMPD4	FERM and PDZ domain containing 4	X
SUM52	RAB9A	RAB9A, member RAS oncogene family	X
SUM52	ASB9	ankyrin repeat and SOCS box-containing 9	X
SUM52	EGFL6	EGF-like-domain, multiple 6	X
SUM52	TLR7	toll-like receptor 7	X
SUM52	GLRA2	glycine receptor, alpha 2	X
SUM52	FIGF	c-fos induced growth factor (vascular endothelial growth factor D)	X
SUM52	FAM9C	family with sequence similarity 9, member C	X
SUM52	ASB11	ankyrin repeat and SOCS box-containing 11	X

Supplementary Table S2: SUM-185 cell line copy number amplified genes.

Cell line	Symbol	Gene name	Chromosome
SUM185	TACC3	transforming, acidic coiled-coil containing protein 3	4
SUM185	FGFR3	fibroblast growth factor receptor 3 (achondroplasia, thanatophoric dwarfism)	4
SUM185	CTBP1	C-terminal binding protein 1	4
SUM185	MAEA	macrophage erythroblast attacher	4
SUM185	CRIPAK	cysteine-rich PAK1 inhibitor	4
SUM185	FGFRL1	fibroblast growth factor receptor-like 1	4
SUM185	IDUA	iduronidase, alpha-L-	4
SUM185	MGC21675	hypothetical protein MGC21675	4
SUM185	KIAA1530	KIAA1530 protein	4
SUM185	TMEM129	transmembrane protein 129	4
SUM185	LETM1	leucine zipper-EF-hand containing transmembrane protein 1	4
SUM185	SLBP	stem-loop (histone) binding protein	4
SUM185	WHSC2	Wolf-Hirschhorn syndrome candidate 2	4
SUM185	WHSC1	Wolf-Hirschhorn syndrome candidate 1	4
SUM185	POLN	polymerase (DNA directed) nu	4
SUM185	TMEM175	transmembrane protein 175	4
SUM185	SLC26A1	solute carrier family 26 (sulfate transporter), member 1	4
SUM185	SPON2	spondin 2, extracellular matrix protein	4
SUM185	MXD4	MAX dimerization protein 4	4
SUM185	RNF212	ring finger protein 212	4
SUM185	NAT8L	N-acetyltransferase 8-like	4
SUM185	DGKQ	diacylglycerol kinase, theta 110kDa	4
SUM185	CXCL3	chemokine (C-X-C motif) ligand 3	4
SUM185	CXCL2	chemokine (C-X-C motif) ligand 2	4
SUM185	MTHFD2L	methylenetetrahydrofolate dehydrogenase (NADP+ dependent) 2-like	4
SUM185	CXCL1	chemokine (C-X-C motif) ligand 1 (melanoma growth stimulating activity, alpha)	4
SUM185	IL8	interleukin 8	4
SUM185	EPGN	epithelial mitogen homolog (mouse)	4
SUM185	ALB	albumin	4
SUM185	ANKRD17	ankyrin repeat domain 17	4
SUM185	COX18	COX18 cytochrome c oxidase assembly homolog (S. cerevisiae)	4
SUM185	PF4	platelet factor 4 (chemokine (C-X-C motif) ligand 4)	4
SUM185	AFP	alpha-fetoprotein	4
SUM185	ADAMTS3	ADAM metallopeptidase with thrombospondin type 1 motif, 3	4
SUM185	RASSF6	Ras association (RalGDS/AF-6) domain family 6	4
SUM185	CXCL5	chemokine (C-X-C motif) ligand 5	4
SUM185	AFM	afamin	4
SUM185	CXCL6	chemokine (C-X-C motif) ligand 6 (granulocyte chemotactic protein 2)	4

SUM185	PF4V1	platelet factor 4 variant 1	4
SUM185	PPBP	pro-platelet basic protein (chemokine (C-X-C motif) ligand 7)	4
SUM185	LRAP	leukocyte-derived arginine aminopeptidase	5
SUM185	RIOK2	RIO kinase 2 (yeast)	5
SUM185	LNPEP	leucyl/cystinyl aminopeptidase	5
SUM185	LIX1	Lix1 homolog (mouse)	5
SUM185	RGMB	RGM domain family, member B	5
SUM185	FAM81B	family with sequence similarity 81, member B	5
SUM185	C5orf36	chromosome 5 open reading frame 36	5
SUM185	MCTP1	multiple C2 domains, transmembrane 1	5
SUM185	ANKRD32	ankyrin repeat domain 32	5
SUM185	VPS35	vacuolar protein sorting 35 homolog (S. cerevisiae)	16
SUM185	GPT2	glutamic pyruvate transaminase (alanine aminotransferase) 2	16
SUM185	LOC388272	similar to RIKEN cDNA 4921524J17	16
SUM185	DNAJA2	DnaJ (Hsp40) homolog, subfamily A, member 2	16
SUM185	SHCBP1	SHC SH2-domain binding protein 1	16
SUM185	PHKB	phosphorylase kinase, beta	16
SUM185	ORC6L	origin recognition complex, subunit 6 like (yeast)	16
SUM185	NETO2	neuropilin (NRP) and tolloid (TLL)-like 2	16
SUM185	ITFG1	integrin alpha FG-GAP repeat containing 1	16
SUM185	MLCK	MLCK protein	16
SUM185	ABCC12	ATP-binding cassette, sub-family C (CFTR/MRP), member 12	16
SUM185	NLRP5	NLR family, pyrin domain containing 5	19
SUM185	C19orf18	chromosome 19 open reading frame 18	19
SUM185	ZNF444	zinc finger protein 444	19
SUM185	ZNF530	zinc finger protein 530	19
SUM185	ZNF71	zinc finger protein 71	19
SUM185	UBE2S	ubiquitin-conjugating enzyme E2S	19
SUM185	NAT14	N-acetyltransferase 14	19
SUM185	ZNF417	zinc finger protein 417	19
SUM185	ZNF582	zinc finger protein 582	19
SUM185	SUV420H2	suppressor of variegation 4-20 homolog 2 (Drosophila)	19
SUM185	ZNF543	zinc finger protein 543	19
SUM185	ZNF587	zinc finger protein 587	19
SUM185	ZNF274	zinc finger protein 274	19
SUM185	ISOC2	isochorismatase domain containing 2	19
SUM185	ZNF580	zinc finger protein 580	19
SUM185	ZSCAN5	zinc finger and SCAN domain containing 5	19
SUM185	ZNF418	zinc finger protein 418	19
SUM185	ZNF304	zinc finger protein 304	19
SUM185	FIZ1	FLT3-interacting zinc finger 1	19
SUM185	VN1R1	vomeronasal 1 receptor 1	19
SUM185	ZNF552	zinc finger protein 552	19
SUM185	ZNF549	zinc finger protein 549	19

SUM185	CCDC106	coiled-coil domain containing 106	19
SUM185	ZNF583	zinc finger protein 583	19
SUM185	ZNF497	zinc finger protein 497	19
SUM185	GALP	galanin-like peptide precursor	19
SUM185	ZNF8	zinc finger protein 8	19
SUM185	ZNF17	zinc finger protein 17	19
SUM185	ZNF628	zinc finger protein 628	19
SUM185	SYT5	synaptotagmin V	19
SUM185	ZNF581	zinc finger protein 581	19
SUM185	ZNF134	zinc finger protein 134	19
SUM185	ZFP28	zinc finger protein 28 homolog (mouse)	19
SUM185	ZNF606	zinc finger protein 606	19
SUM185	U2AF2	U2 small nuclear RNA auxiliary factor 2	19
SUM185	ZNF211	zinc finger protein 211	19
SUM185	ZSCAN4	zinc finger and SCAN domain containing 4	19
SUM185	TNNI3	troponin I type 3 (cardiac)	19
SUM185	ZNF524	zinc finger protein 524	19
SUM185	ZNF551	zinc finger protein 551	19
SUM185	ZNF329	zinc finger protein 329	19
SUM185	TMEM190	transmembrane protein 190	19
SUM185	ZNF671	zinc finger protein 671	19
SUM185	PPP1R12C	protein phosphatase 1, regulatory (inhibitor) subunit 12C	19
SUM185	ZNF776	zinc finger protein 776	19
SUM185	ZSCAN22	zinc finger and SCAN domain containing 22	19
SUM185	SAPS1	SAPS domain family, member 1	19
SUM185	ZNF667	zinc finger protein 667	19
SUM185	ZNF416	zinc finger protein 416	19
SUM185	ZNF419	zinc finger protein 419	19
SUM185	ZNF264	zinc finger protein 264	19
SUM185	ZNF784	zinc finger protein 784	19
SUM185	NLRP11	NLR family, pyrin domain containing 11	19
SUM185	ZNF787	zinc finger protein 787	19
SUM185	ZNF256	zinc finger protein 256	19
SUM185	EPS8L1	EPS8-like 1	19
SUM185	AURKC	aurora kinase C	19
SUM185	ZNF135	zinc finger protein 135	19
SUM185	HSPBP1	hsp70-interacting protein	19
SUM185	ZNF579	zinc finger protein 579	19
SUM185	ZNF471	zinc finger protein 471	19
SUM185	TMEM86B	transmembrane protein 86B	19
SUM185	RPL28	ribosomal protein L28	19
SUM185	ZIK1	zinc finger protein interacting with K protein 1 homolog (mouse)	19
SUM185	RDH13	retinol dehydrogenase 13 (all-trans/9-cis)	19
SUM185	BRSK1	BR serine/threonine kinase 1	19

SUM185	NLRP4	NLR family, pyrin domain containing 4	19
SUM185	TNNT1	troponin T type 1 (skeletal, slow)	19
SUM185	ZNF544	zinc finger protein 544	19
SUM185	ZIM3	zinc finger, imprinted 3	19
SUM185	EPN1	epsin 1	19
SUM185	ZSCAN1	zinc finger and SCAN domain containing 1	19
SUM185	PEG3	paternally expressed 3	19
SUM185	COX6B2	cytochrome c oxidase subunit VIb polypeptide 2 (testis)	19
SUM185	IL11	interleukin 11	19
SUM185	BC37295_3	hypothetical BC37295_3	19
SUM185	PTPRH	protein tyrosine phosphatase, receptor type, H	19
SUM185	ZSCAN18	zinc finger and SCAN domain containing 18	19
SUM185	RPS5	ribosomal protein S5	19
SUM185	NLRP9	NLR family, pyrin domain containing 9	19
SUM185	NLRP13	NLR family, pyrin domain containing 13	19
SUM185	NLRP8	NLR family, pyrin domain containing 8	19
SUM185	USP29	ubiquitin specific peptidase 29	19
SUM185	ZNF14	zinc finger protein 14	19
SUM185	ZNF486	zinc finger protein 486	19
SUM185	UPF1	UPF1 regulator of nonsense transcripts homolog (yeast)	19
SUM185	ZNF626	zinc finger protein 626	19
SUM185	ISYNA1	myo-inositol 1-phosphate synthase A1	19
SUM185	ZNF253	zinc finger protein 253	19
SUM185	CRLF1	cytokine receptor-like factor 1	19
SUM185	GATAD2A	GATA zinc finger domain containing 2A	19
SUM185	ZNF682	zinc finger protein 682	19
SUM185	KIAA0892	KIAA0892	19
SUM185	ZNF93	zinc finger protein 93	19
SUM185	SF4	splicing factor 4	19
SUM185	KLHL26	kelch-like 26 (Drosophila)	19
SUM185	ATP13A1	ATPase type 13A1	19
SUM185	ELL	elongation factor RNA polymerase II	19
SUM185	DDX49	DEAD (Asp-Glu-Ala-Asp) box polypeptide 49	19
SUM185	COPE	coatomer protein complex, subunit epsilon	19
SUM185	SFRS14	splicing factor, arginine/serine-rich 14	19
SUM185	NDUFA13	NADH dehydrogenase (ubiquinone) 1 alpha subcomplex, 13	19
SUM185	SSBP4	single stranded DNA binding protein 4	19
SUM185	GMIP	GEM interacting protein	19
SUM185	TRA16	TR4 orphan receptor associated protein TRA16	19
SUM185	TMEM161A	transmembrane protein 161A	19
SUM185	LASS1	LAG1 homolog, ceramide synthase 1	19
SUM185	RFXANK	regulatory factor X-associated ankyrin-containing protein	19
SUM185	COMP	cartilage oligomeric matrix protein	19
SUM185	CILP2	cartilage intermediate layer protein 2	19
SUM185	UBA52	ubiquitin A-52 residue ribosomal protein fusion product 1	19

SUM185	FKBP8	FK506 binding protein 8, 38kDa	19
SUM185	ARMC6	armadillo repeat containing 6	19
SUM185	HOMER3	homer homolog 3 (Drosophila)	19
SUM185	ZNF101	zinc finger protein 101	19
SUM185	NCAN	neurocan	19
SUM185	EDG4	endothelial differentiation, lysophosphatidic acid G-protein-coupled receptor, 4	19
SUM185	SLC25A42	solute carrier family 25, member 42	19
SUM185	FLJ44894	similar to zinc finger protein 91	19
SUM185	PBX4	pre-B-cell leukemia homeobox 4	19
SUM185	TM6SF2	transmembrane 6 superfamily member 2	19
SUM185	TMEM59L	transmembrane protein 59-like	19
SUM185	TSSK6	testis-specific serine kinase 6	19
SUM185	HAPLN4	hyaluronan and proteoglycan link protein 4	19
SUM185	C19orf50	chromosome 19 open reading frame 50	19
SUM185	PRPF31	PRP31 pre-mRNA processing factor 31 homolog (S. cerevisiae)	19
SUM185	TFPT	TCF3 (E2A) fusion partner (in childhood Leukemia)	19
SUM185	CNOT3	CCR4-NOT transcription complex, subunit 3	19
SUM185	NDUFA3	NADH dehydrogenase (ubiquinone) 1 alpha subcomplex, 3, 9kDa	19
SUM185	CACNG8	calcium channel, voltage-dependent, gamma subunit 8	19
SUM185	CACNG7	calcium channel, voltage-dependent, gamma subunit 7	19
SUM185	OSCAR	osteoclast associated, immunoglobulin-like receptor	19
SUM185	CACNG6	calcium channel, voltage-dependent, gamma subunit 6	19
SUM185	ZNF766	zinc finger protein 766	19
SUM185	ZNF649	zinc finger protein 649	19
SUM185	ZNF616	zinc finger protein 616	19
SUM185	ZNF28	zinc finger protein 28	19
SUM185	ZNF320	zinc finger protein 320	19
SUM185	ZNF432	zinc finger protein 432	19
SUM185	ZNF468	zinc finger protein 468	19
SUM185	ZNF350	zinc finger protein 350	19
SUM185	ZNF613	zinc finger protein 613	19
SUM185	ZNF83	zinc finger protein 83	19
SUM185	ZNF610	zinc finger protein 610	19
SUM185	ZNF615	zinc finger protein 615	19
SUM185	ZNF137	zinc finger protein 137	19
SUM185	CLDND2	claudin domain containing 2	19
SUM185	ZNF528	zinc finger protein 528	19
SUM185	ZNF808	zinc finger protein 808	19
SUM185	PPP2R1A	protein phosphatase 2 (formerly 2A), regulatory subunit A , alpha isoform	19
SUM185	ZNF480	zinc finger protein 480	19
SUM185	ZNF577	zinc finger protein 577	19
SUM185	ETFB	electron-transfer-flavoprotein, beta polypeptide	19

SUM185	ZNF175	zinc finger protein 175	19
SUM185	ZNF600	zinc finger protein 600	19
SUM185	SIGLEC6	sialic acid binding Ig-like lectin 6	19
SUM185	ZNF160	zinc finger protein 160	19
SUM185	ZNF614	zinc finger protein 614	19
SUM185	FPR1	formyl peptide receptor 1	19
SUM185	FPRL1	formyl peptide receptor-like 1	19
SUM185	NKG7	natural killer cell group 7 sequence	19
SUM185	LIM2	lens intrinsic membrane protein 2, 19kDa	19
SUM185	SIGLEC5	sialic acid binding Ig-like lectin 5	19
SUM185	FPRL2	formyl peptide receptor-like 2	19
SUM185	FLJ40235	hypothetical protein FLJ40235	19
SUM185	SIGLEC10	sialic acid binding Ig-like lectin 10	19
SUM185	SIGLEC8	sialic acid binding Ig-like lectin 8	19
SUM185	SIGLEC12	sialic acid binding Ig-like lectin 12	19
SUM185	HAS1	hyaluronan synthase 1	19
SUM185	PLAGL2	pleiomorphic adenoma gene-like 2	20
SUM185	KIF3B	kinesin family member 3B	20
SUM185	TM9SF4	transmembrane 9 superfamily protein member 4	20
SUM185	PDRG1	p53 and DNA damage regulated 1	20
SUM185	HM13	histocompatibility (minor) 13	20
SUM185	ASXL1	additional sex combs like 1 (Drosophila)	20
SUM185	MYLK2	myosin light chain kinase 2, skeletal muscle	20
SUM185	POFUT1	protein O-fucosyltransferase 1	20
SUM185	C20orf112	chromosome 20 open reading frame 112	20
SUM185	FKHL18	forkhead-like 18 (Drosophila)	20
SUM185	TPX2	TPX2, microtubule-associated, homolog (Xenopus laevis)	20
SUM185	C20orf160	chromosome 20 open reading frame 160	20
SUM185	BCL2L1	BCL2-like 1	20
SUM185	ID1	inhibitor of DNA binding 1, dominant negative helix-loop-helix protein	20
SUM185	HCK	hemopoietic cell kinase	20
SUM185	COMMD7	COMM domain containing 7	20
SUM185	REM1	RAS (RAD and GEM)-like GTP-binding 1	20
SUM185	COX4I2	cytochrome c oxidase subunit IV isoform 2 (lung)	20
SUM185	DUSP15	dual specificity phosphatase 15	20
SUM185	C20orf3	chromosome 20 open reading frame 3	20
SUM185	GZF1	GDNF-inducible zinc finger protein 1	20
SUM185	ABHD12	abhydrolase domain containing 12	20
SUM185	NAPB	N-ethylmaleimide-sensitive factor attachment protein, beta	20
SUM185	CST5	cystatin D	20
SUM185	CST3	cystatin C (amyloid angiopathy and cerebral hemorrhage)	20
SUM185	ACSS1	acyl-CoA synthetase short-chain family member 1	20
SUM185	ZNF337	zinc finger protein 337	20
SUM185	NKX2-2	NK2 homeobox 2	20
SUM185	RP4-691N24.1	KIAA0980 protein	20

SUM185	NXT1	NTF2-like export factor 1	20
SUM185	NANP	N-acetylneuraminic acid phosphatase	20
SUM185	GGTLA4	gamma-glutamyltransferase-like activity 4	20
SUM185	ENTPD6	ectonucleoside triphosphate diphosphohydrolase 6 (putative function)	20
SUM185	THBD	thrombomodulin	20
SUM185	C20orf39	chromosome 20 open reading frame 39	20
SUM185	GINS1	GINS complex subunit 1 (Psf1 homolog)	20
SUM185	PYGB	phosphorylase, glycogen; brain	20
SUM185	FOXA2	forkhead box A2	20
SUM185	VSX1	visual system homeobox 1	20
SUM185	SSTR4	somatostatin receptor 4	20
SUM185	CST1	cystatin SN	20
SUM185	CD93	CD93 molecule	20
SUM185	CST2	cystatin SA	20
SUM185	PAX1	paired box 1	20
SUM185	CSTL1	cystatin-like 1	20
SUM185	CST11	cystatin 11	20
SUM185	CST8	cystatin 8 (cystatin-related epididymal specific)	20
SUM185	CST9L	cystatin 9-like (mouse)	20
SUM185	CST9	cystatin 9 (testatin)	20
SUM185	CST7	cystatin F (leukocystatin)	20
SUM185	DEFB118	defensin, beta 118	20

Supplementary Table S3: SUM-190 cell line copy number amplified genes.

Cell line	Symbol	Gene name	Chromosome
SUM190	OR10J5	olfactory receptor, family 10, subfamily J, member 5	1
SUM190	DUSP23	dual specificity phosphatase 23	1
SUM190	APCS	amyloid P component, serum	1
SUM190	OR10J1	olfactory receptor, family 10, subfamily J, member 1	1
SUM190	CRP	C-reactive protein, pentraxin-related	1
SUM190	CENPC1	centromere protein C 1	4
SUM190	TMPRSS11D	transmembrane protease, serine 11D	4
SUM190	UBE1L2	ubiquitin-activating enzyme E1-like 2	4
SUM190	STAP1	signal transducing adaptor family member 1	4
SUM190	GNRHR	gonadotropin-releasing hormone receptor	4
SUM190	EPHA5	EPH receptor A5	4
SUM190	TMPRSS11A	transmembrane protease, serine 11A	4
SUM190	TMPRSS11F	transmembrane protease, serine 11F	4
SUM190	EPGN	epithelial mitogen homolog (mouse)	4
SUM190	CXCL2	chemokine (C-X-C motif) ligand 2	4
SUM190	EREG	epiregulin	4
SUM190	CXCL3	chemokine (C-X-C motif) ligand 3	4
SUM190	MTHFD2L	methylenetetrahydrofolate dehydrogenase (NADP+ dependent) 2-like	4
SUM190	COX18	COX18 cytochrome c oxidase assembly homolog (S. cerevisiae)	4
SUM190	GRSF1	G-rich RNA sequence binding factor 1	4
SUM190	CXCL1	chemokine (C-X-C motif) ligand 1 (melanoma growth stimulating activity, alpha)	4
SUM190	SAS10	disrupter of silencing 10	4
SUM190	MOBKL1A	MOB1, Mps One Binder kinase activator-like 1A (yeast)	4
SUM190	CXCL5	chemokine (C-X-C motif) ligand 5	4
SUM190	ADAMTS3	ADAM metallopeptidase with thrombospondin type 1 motif, 3	4
SUM190	ANKRD17	ankyrin repeat domain 17	4
SUM190	DCK	deoxycytidine kinase	4
SUM190	AFP	alpha-fetoprotein	4
SUM190	ALB	albumin	4
SUM190	RUFY3	RUN and FYVE domain containing 3	4
SUM190	NPFFR2	neuropeptide FF receptor 2	4
SUM190	SLC4A4	solute carrier family 4, sodium bicarbonate cotransporter, member 4	4
SUM190	PF4	platelet factor 4 (chemokine (C-X-C motif) ligand 4)	4
SUM190	IGJ	immunoglobulin J polypeptide, linker protein for immunoglobulin alpha and mu polypeptides	4
SUM190	CXCL6	chemokine (C-X-C motif) ligand 6 (granulocyte chemotactic protein 2)	4
SUM190	CXCL3	chemokine (C-X-C motif) ligand 3	4
SUM190	RASSF6	Ras association (RalGDS/AF-6) domain family 6	4

**SUM190**	**IL8**	**interleukin 8**	**4**
**SUM190**	**AMBN**	**ameloblastin (enamel matrix protein)**	**4**
**SUM190**	**ENAM**	**enamelin**	**4**
**SUM190**	**GC**	**group-specific component (vitamin D binding protein)**	**4**
**SUM190**	**AFM**	**afamin**	**4**
**SUM190**	**PF4V1**	**platelet factor 4 variant 1**	**4**
**SUM190**	**PPBP**	**pro-platelet basic protein (chemokine (C-X-C motif) ligand 7)**	**4**
**SUM190**	**ASPH**	**aspartate beta-hydroxylase**	**8**
**SUM190**	**CHD7**	**chromodomain helicase DNA binding protein 7**	**8**
**SUM190**	**GGH**	**gamma-glutamyl hydrolase (conjugase, folylpolygammaglutamyl hydrolase)**	**8**
**SUM190**	**RAB2A**	**RAB2A, member RAS oncogene family**	**8**
**SUM190**	**RLBP1L1**	**retinaldehyde binding protein 1-like 1**	**8**
**SUM190**	**FAM77D**	**family with sequence similarity 77, member D**	**8**
**SUM190**	**YTHDF3**	**YTH domain family, member 3**	**8**
**SUM190**	**BHLHB5**	**basic helix-loop-helix domain containing, class B, 5**	**8**
**SUM190**	**GGH**	**gamma-glutamyl hydrolase (conjugase, folylpolygammaglutamyl hydrolase)**	**8**
**SUM190**	**TTPA**	**tocopherol (alpha) transfer protein**	**8**
**SUM190**	**FAM77D**	**family with sequence similarity 77, member D**	**8**
**SUM190**	**NCALD**	**neurocalcin delta**	**8**
**SUM190**	**DPYS**	**dihydropyrimidinase**	**8**
**SUM190**	**OSGIN2**	**oxidative stress induced growth inhibitor family member 2**	**8**
**SUM190**	**ATP6V0D2**	**ATPase, H+ transporting, lysosomal 38kDa, V0 subunit d2**	**8**
**SUM190**	**PABPC1**	**poly(A) binding protein, cytoplasmic 1**	**8**
**SUM190**	**EFCBP1**	**EF-hand calcium binding protein 1**	**8**
**SUM190**	**MATN2**	**matrilin 2**	**8**
**SUM190**	**TTC35**	**tetratricopeptide repeat domain 35**	**8**
**SUM190**	**RIMS2**	**regulating synaptic membrane exocytosis 2**	**8**
**SUM190**	**ATP6V1C1**	**ATPase, H+ transporting, lysosomal 42kDa, V1 subunit C1**	**8**
**SUM190**	**RNF19A**	**ring finger protein 19A**	**8**
**SUM190**	**NPAL2**	**NIPA-like domain containing 2**	**8**
**SUM190**	**LAPTM4B**	**lysosomal associated protein transmembrane 4 beta**	**8**
**SUM190**	**UQCRB**	**ubiquinol-cytochrome c reductase binding protein**	**8**
**SUM190**	**PGCP**	**plasma glutamate carboxypeptidase**	**8**
**SUM190**	**SLC26A7**	**solute carrier family 26, member 7**	**8**
**SUM190**	**INTS8**	**integrator complex subunit 8**	**8**
**SUM190**	**PMP2**	**peripheral myelin protein 2**	**8**
**SUM190**	**GDF6**	**growth differentiation factor 6**	**8**
**SUM190**	**TMEM67**	**transmembrane protein 67**	**8**
**SUM190**	**CA3**	**carbonic anhydrase III, muscle specific**	**8**
**SUM190**	**WWP1**	**WW domain containing E3 ubiquitin protein ligase 1**	**8**
**SUM190**	**SPAG1**	**sperm associated antigen 1**	**8**

SUM190	RGS22	regulator of G-protein signaling 22	8
SUM190	C8orf37	chromosome 8 open reading frame 37	8
SUM190	PPM2C	protein phosphatase 2C, magnesium-dependent, catalytic subunit	8
SUM190	TSPYL5	TSPY-like 5	8
SUM190	RPL30	ribosomal protein L30	8
SUM190	PLEKHF2	pleckstrin homology domain containing, family F (with FYVE domain) member 2	8
SUM190	OXR1	oxidation resistance 1	8
SUM190	EIF3E	eukaryotic translation initiation factor 3, subunit E	8
SUM190	POLR2K	polymerase (RNA) II (DNA directed) polypeptide K, 7.0kDa	8
SUM190	HRSP12	heat-responsive protein 12	8
SUM190	SDC2	syndecan 2	8
SUM190	GRHL2	grainyhead-like 2 (Drosophila)	8
SUM190	COX6C	cytochrome c oxidase subunit VIc	8
SUM190	VPS13B	vacuolar protein sorting 13 homolog B (yeast)	8
SUM190	ZNF706	zinc finger protein 706	8
SUM190	RBM35A	RNA binding motif protein 35A	8
SUM190	TM7SF4	transmembrane 7 superfamily member 4	8
SUM190	KIAA1429	KIAA1429	8
SUM190	CA13	carbonic anhydrase XIII	8
SUM190	C8orf47	chromosome 8 open reading frame 47	8
SUM190	LRRCC1	leucine rich repeat and coiled-coil domain containing 1	8
SUM190	OSR2	odd-skipped related 2 (Drosophila)	8
SUM190	CHMP4C	chromatin modifying protein 4C	8
SUM190	POP1	processing of precursor 1, ribonuclease P/MRP subunit (S. cerevisiae)	8
SUM190	NBN	nibrin	8
SUM190	CA1	carbonic anhydrase I	8
SUM190	CA2	carbonic anhydrase II	8
SUM190	ANKRD46	ankyrin repeat domain 46	8
SUM190	RAD54B	RAD54 homolog B (S. cerevisiae)	8
SUM190	PSKH2	protein serine kinase H2	8
SUM190	MMP16	matrix metallopeptidase 16 (membrane-inserted)	8
SUM190	SNX16	sorting nexin 16	8
SUM190	TMEM55A	transmembrane protein 55A	8
SUM190	DECR1	2,4-dienoyl CoA reductase 1, mitochondrial	8
SUM190	ANGPT1	angiopoietin 1	8
SUM190	GEM	GTP binding protein overexpressed in skeletal muscle	8
SUM190	BAALC	brain and acute leukemia, cytoplasmic	8
SUM190	LRP12	low density lipoprotein-related protein 12	8
SUM190	ZFPM2	zinc finger protein, multitype 2	8
SUM190	CPNE3	copine III	8
SUM190	IMPA1	inositol(myo)-1(or 4)-monophosphatase 1	8
SUM190	WDSOF1	WD repeats and SOF1 domain containing	8

SUM190	CALB1	calbindin 1, 28kDa	8
SUM190	C8orf38	chromosome 8 open reading frame 38	8
SUM190	RIPK2	receptor-interacting serine-threonine kinase 2	8
SUM190	KCNS2	potassium voltage-gated channel, delayed-rectifier, subfamily S, member 2	8
SUM190	TP53INP1	tumor protein p53 inducible nuclear protein 1	8
SUM190	UBR5	ubiquitin protein ligase E3 component n-recognin 5	8
SUM190	RBM12B	RNA binding motif protein 12B	8
SUM190	C8orf59	chromosome 8 open reading frame 59	8
SUM190	YWHAZ	tyrosine 3-monooxygenase/tryptophan 5-monooxygenase activation protein, zeta polypeptide	8
SUM190	MTERFD1	MTERF domain containing 1	8
SUM190	RRM2B	ribonucleotide reductase M2 B (TP53 inducible)	8
SUM190	RUNX1T1	runt-related transcription factor 1; translocated to, 1 (cyclin D-related)	8
SUM190	DPY19L4	dpy-19-like 4 (C. elegans)	8
SUM190	E2F5	E2F transcription factor 5, p130-binding	8
SUM190	SLC7A13	solute carrier family 7, (cationic amino acid transporter, y+ system) member 13	8
SUM190	TMEM64	transmembrane protein 64	8
SUM190	MGC39715	hypothetical protein MGC39715	8
SUM190	WDR21C	WD repeat domain 21C	8
SUM190	CTHRC1	collagen triple helix repeat containing 1	8
SUM190	OTUD6B	OTU domain containing 6B	8
SUM190	SLC25A32	solute carrier family 25, member 32	8
SUM190	FABP4	fatty acid binding protein 4, adipocyte	8
SUM190	CCNE2	cyclin E2	8
SUM190	RSPO2	R-spondin 2 homolog (Xenopus laevis)	8
SUM190	MTDH	metadherin	8
SUM190	ZFAND1	zinc finger, AN1-type domain 1	8
SUM190	PTDSS1	phosphatidylserine synthase 1	8
SUM190	STK3	serine/threonine kinase 3 (STE20 homolog, yeast)	8
SUM190	FAM82B	family with sequence similarity 82, member B	8
SUM190	FZD6	frizzled homolog 6 (Drosophila)	8
SUM190	AZIN1	antizyme inhibitor 1	8
SUM190	KLF10	Kruppel-like factor 10	8
SUM190	RALYL	RALY RNA binding protein-like	8
SUM190	REXO1L1	REX1, RNA exonuclease 1 homolog (S. cerevisiae)-like 1	8
SUM190	CNGB3	cyclic nucleotide gated channel beta 3	8
SUM190	CNBD1	cyclic nucleotide binding domain containing 1	8
SUM190	CDH17	cadherin 17, LI cadherin (liver-intestine)	8
SUM190	ODF1	outer dense fiber of sperm tails 1	8
SUM190	ABRA	actin-binding Rho activating protein	8
SUM190	TMEM74	transmembrane protein 74	8
SUM190	TRHR	thyrotropin-releasing hormone receptor	8
SUM190	DNAJC12	DnaJ (Hsp40) homolog, subfamily C, member 12	10

SUM190	SIRT1	sirtuin (silent mating type information regulation 2 homolog) 1 (S. cerevisiae)	10
SUM190	MYPN	myopalladin	10
SUM190	HERC4	hect domain and RLD 4	10
SUM190	ATOH7	atonal homolog 7 (Drosophila)	10
SUM190	LRRTM3	leucine rich repeat transmembrane neuronal 3	10
SUM190	PPME1	protein phosphatase methylesterase 1	11
SUM190	DKFZP586P0123	hypothetical protein	11
SUM190	POLD3	polymerase (DNA-directed), delta 3, accessory subunit	11
SUM190	SPCS2	signal peptidase complex subunit 2 homolog (S. cerevisiae)	11
SUM190	UCP3	uncoupling protein 3 (mitochondrial, proton carrier)	11
SUM190	ARRB1	arrestin, beta 1	11
SUM190	UCP2	uncoupling protein 2 (mitochondrial, proton carrier)	11
SUM190	P4HA3	procollagen-proline, 2-oxoglutarate 4-dioxygenase (proline 4-hydroxylase), alpha polypeptide III	11
SUM190	CHRDL2	chordin-like 2	11
SUM190	PGM2L1	phosphoglucomutase 2-like 1	11
SUM190	NEU3	sialidase 3 (membrane sialidase)	11
SUM190	SLCO2B1	solute carrier organic anion transporter family, member 2B1	11
SUM190	KCNE3	potassium voltage-gated channel, Isk-related family, member 3	11
SUM190	C11orf30	chromosome 11 open reading frame 30	11
SUM190	TSKU	tsukushin	11
SUM190	PHCA	phytoceramidase, alkaline	11
SUM190	PRKRIR	protein-kinase, interferon-inducible double stranded RNA dependent inhibitor, repressor of (P58 repressor)	11
SUM190	LRRC32	leucine rich repeat containing 32	11
SUM190	B3GNT6	UDP-GlcNAc:betaGal beta-1,3-N-acetylglucosaminyltransferase 6 (core 3 synthase)	11
SUM190	MED19	mediator complex subunit 19	11
SUM190	C11orf31	chromosome 11 open reading frame 31	11
SUM190	TXNDC14	thioredoxin domain containing 14	11
SUM190	ZDHHC5	zinc finger, DHHC-type containing 5	11
SUM190	TIMM10	translocase of inner mitochondrial membrane 10 homolog (yeast)	11
SUM190	SLC43A1	solute carrier family 43, member 1	11
SUM190	CTNND1	catenin (cadherin-associated protein), delta 1	11
SUM190	CLP1	CLP1, cleavage and polyadenylation factor I subunit, homolog (S. cerevisiae)	11
SUM190	UBE2L6	ubiquitin-conjugating enzyme E2L 6	11
SUM190	RTN4RL2	reticulon 4 receptor-like 2	11
SUM190	YPEL4	yippee-like 4 (Drosophila)	11
SUM190	OR1S2	olfactory receptor, family 1, subfamily S, member 2	11
SUM190	SERPING1	serpin peptidase inhibitor, clade G (C1 inhibitor), member 1, (angioedema, hereditary)	11

SUM190	LPXN	leupaxin	11
SUM190	TMEM126A	transmembrane protein 126A	11
SUM190	C11orf73	chromosome 11 open reading frame 73	11
SUM190	CREBZF	CREB/ATF bZIP transcription factor	11
SUM190	TMEM126B	transmembrane protein 126B	11
SUM190	EED	embryonic ectoderm development	11
SUM190	PICALM	phosphatidylinositol binding clathrin assembly protein	11
SUM190	SYTL2	synaptotagmin-like 2	11
SUM190	DLG2	discs, large homolog 2, chapsyn-110 (Drosophila)	11
SUM190	CCDC83	coiled-coil domain containing 83	11
SUM190	PRSS23	protease, serine, 23	11
SUM190	ME3	malic enzyme 3, NADP(+)-dependent, mitochondrial	11
SUM190	CCDC81	coiled-coil domain containing 81	11
SUM190	CCDC89	coiled-coil domain containing 89	11
SUM190	ORAOV1	oral cancer overexpressed 1	11
SUM190	PPFIA1	protein tyrosine phosphatase, receptor type, f polypeptide (PTPRF), interacting protein (liprin), alpha 1	11
SUM190	CCND1	cyclin D1	11
SUM190	FADD	Fas (TNFRSF6)-associated via death domain	11
SUM190	CTTN	cortactin	11
SUM190	MYEOV	myeloma overexpressed gene (in a subset of t(11;14) positive multiple myelomas)	11
SUM190	TPCN2	two pore segment channel 2	11
SUM190	DHCR7	7-dehydrocholesterol reductase	11
SUM190	FGF19	fibroblast growth factor 19	11
SUM190	SHANK2	SH3 and multiple ankyrin repeat domains 2	11
SUM190	FGF3	fibroblast growth factor 3 (murine mammary tumor virus integration site (v-int-2) oncogene homolog)	11
SUM190	TMEM16A	transmembrane protein 16A	11
SUM190	FGF4	fibroblast growth factor 4 (heparin secretory transforming protein 1, Kaposi sarcoma oncogene)	11
SUM190	THRSP	thyroid hormone responsive (SPOT14 homolog, rat)	11
SUM190	INTS4	integrator complex subunit 4	11
SUM190	PAK1	p21/Cdc42/Rac1-activated kinase 1 (STE20 homolog, yeast)	11
SUM190	C11orf67	chromosome 11 open reading frame 67	11
SUM190	CLNS1A	chloride channel, nucleotide-sensitive, 1A	11
SUM190	ALG8	asparagine-linked glycosylation 8 homolog (S. cerevisiae, alpha-1,3-glucosyltransferase)	11
SUM190	RSF1	remodeling and spacing factor 1	11
SUM190	NDUFC2	NADH dehydrogenase (ubiquinone) 1, subcomplex unknown, 2, 14.5kDa	11
SUM190	AQP11	aquaporin 11	11
SUM190	KCTD21	potassium channel tetramerisation domain containing 21	11
SUM190	C11orf30	chromosome 11 open reading frame 30	11
SUM190	WNT11	wingless-type MMTV integration site family, member 11	11

SUM190	UVRAG	UV radiation resistance associated gene	11
SUM190	PRKRIR	protein-kinase, interferon-inducible double stranded RNA dependent inhibitor, repressor of (P58 repressor)	11
SUM190	C16orf71	chromosome 16 open reading frame 71	16
SUM190	NMRAL1	NmrA-like family domain containing 1	16
SUM190	12-Sep	septin 12	16
SUM190	ZNF174	zinc finger protein 174	16
SUM190	MGRN1	mahogunin, ring finger 1	16
SUM190	HMOX2	heme oxygenase (decycling) 2	16
SUM190	ZNF500	zinc finger protein 500	16
SUM190	ROGDI	rogdi homolog (Drosophila)	16
SUM190	DNAJA3	DnaJ (Hsp40) homolog, subfamily A, member 3	16
SUM190	Magmas	mitochondria-associated protein involved in granulocyte-macrophage colony-stimulating factor signal transduction	16
SUM190	ALG1	asparagine-linked glycosylation 1 homolog (S. cerevisiae, beta-1,4-mannosyltransferase)	16
SUM190	BTBD12	BTB (POZ) domain containing 12	16
SUM190	FAM100A	family with sequence similarity 100, member A	16
SUM190	ZNF205	zinc finger protein 205	16
SUM190	C16orf5	chromosome 16 open reading frame 5	16
SUM190	ANKS3	ankyrin repeat and sterile alpha motif domain containing 3	16
SUM190	DNASE1	deoxyribonuclease I	16
SUM190	NUDT16L1	nudix (nucleoside diphosphate linked moiety X)-type motif 16-like 1	16
SUM190	NAGPA	N-acetylglucosamine-1-phosphodiester alpha-N-acetylglucosaminidase	16
SUM190	UBN1	ubinuclein 1	16
SUM190	ZNF213	zinc finger protein 213	16
SUM190	ZNF200	zinc finger protein 200	16
SUM190	CREBBP	CREB binding protein (Rubinstein-Taybi syndrome)	16
SUM190	C16orf68	chromosome 16 open reading frame 68	16
SUM190	ZNF75A	zinc finger protein 75a	16
SUM190	ZNF434	zinc finger protein 434	16
SUM190	ZNF263	zinc finger protein 263	16
SUM190	N-PAC	cytokine-like nuclear factor n-pac	16
SUM190	OR1F1	olfactory receptor, family 1, subfamily F, member 1	16
SUM190	TIGD7	tigger transposable element derived 7	16
SUM190	VASN	vasorin	16
SUM190	TRAP1	TNF receptor-associated protein 1	16
SUM190	CLUAP1	clusterin associated protein 1	16
SUM190	FLJ14154	hypothetical protein FLJ14154	16
SUM190	ZSCAN10	zinc finger and SCAN domain containing 10	16
SUM190	ZNF597	zinc finger protein 597	16
SUM190	CORO7	coronin 7	16

SUM190	PPL	periplakin	16
SUM190	MGC45438	hypothetical protein MGC45438	16
SUM190	ADCY9	adenylate cyclase 9	16
SUM190	A2BP1	ataxin 2-binding protein 1	16
SUM190	GLIS2	GLIS family zinc finger 2	16
SUM190	NLRC3	NLR family, CARD domain containing 3	16
SUM190	OR2C1	olfactory receptor, family 2, subfamily C, member 1	16
SUM190	FAM86A	family with sequence similarity 86, member A	16
SUM190	MEFV	Mediterranean fever	16
SUM190	ABAT	4-aminobutyrate aminotransferase	16
SUM190	TFAP4	transcription factor AP-4 (activating enhancer binding protein 4)	16
SUM190	GOSR1	golgi SNAP receptor complex member 1	17
SUM190	CPD	carboxypeptidase D	17
SUM190	CRLF3	cytokine receptor-like factor 3	17
SUM190	DKFZP434O047	DKFZP434O047 protein	17
SUM190	ERBB2	v-erb-b2 erythroblastic leukemia viral oncogene homolog 2, neuro/glioblastoma derived oncogene homolog (avian)	17
SUM190	C17orf37	chromosome 17 open reading frame 37	17
SUM190	PERLD1	per1-like domain containing 1	17
SUM190	GRB7	growth factor receptor-bound protein 7	17
SUM190	TCAP	titin-cap (telethonin)	17
SUM190	PNMT	phenylethanolamine N-methyltransferase	17
SUM190	STARD3	StAR-related lipid transfer (START) domain containing 3	17
SUM190	ORMDL3	ORM1-like 3 (S. cerevisiae)	17
SUM190	PSMD3	proteasome (prosome, macropain) 26S subunit, non-ATPase, 3	17
SUM190	THRA	thyroid hormone receptor, alpha (erythroblastic leukemia viral (v-erb-a) oncogene homolog, avian)	17
SUM190	GSDML	gasdermin-like	17
SUM190	MED24	mediator complex subunit 24	17
SUM190	NR1D1	nuclear receptor subfamily 1, group D, member 1	17
SUM190	GRB7	growth factor receptor-bound protein 7	17
SUM190	GSDM1	gasdermin 1	17
SUM190	CSF3	colony stimulating factor 3 (granulocyte)	17
SUM190	MSL-1	male-specific lethal-1 homolog	17
SUM190	IKZF3	IKAROS family zinc finger 3 (Aiolos)	17
SUM190	ZPBP2	zona pellucida binding protein 2	17
SUM190	NBR1	neighbor of BRCA1 gene 1	17
SUM190	TMEM106A	transmembrane protein 106A	17
SUM190	BRCA1	breast cancer 1, early onset	17
SUM190	IFI35	interferon-induced protein 35	17
SUM190	RND2	Rho family GTPase 2	17
SUM190	RPL27	ribosomal protein L27	17
SUM190	VAT1	vesicle amine transport protein 1 homolog (T. californica)	17

SUM190	ZNF207	zinc finger protein 207	17
SUM190	PSMD11	proteasome (prosome, macropain) 26S subunit, non-ATPase, 11	17
SUM190	CDK5R1	cyclin-dependent kinase 5, regulatory subunit 1 (p35)	17
SUM190	TMEM98	transmembrane protein 98	17
SUM190	MYO1D	myosin ID	17
SUM190	CCL2	chemokine (C-C motif) ligand 2	17
SUM190	ACCN1	amiloride-sensitive cation channel 1, neuronal (degenerin)	17
SUM190	C17orf75	chromosome 17 open reading frame 75	17
SUM190	SPACA3	sperm acrosome associated 3	17
SUM190	LGALS9	lectin, galactoside-binding, soluble, 9 (galectin 9)	17
SUM190	WSB1	WD repeat and SOCS box-containing 1	17
SUM190	NLK	nemo-like kinase	17
SUM190	FAM27L	family with sequence similarity 27-like	17
SUM190	FLJ40504	hypothetical protein FLJ40504	17
SUM190	KSR1	kinase suppressor of ras 1	17
SUM190	NOS2A	nitric oxide synthase 2A (inducible, hepatocytes)	17
SUM190	LIG3	ligase III, DNA, ATP-dependent	17
SUM190	CCT6B	chaperonin containing TCP1, subunit 6B (zeta 2)	17
SUM190	RFFL	ring finger and FYVE-like domain containing 1	17
SUM190	CCL7	chemokine (C-C motif) ligand 7	17
SUM190	CCDC16	coiled-coil domain containing 16	17
SUM190	CCL8	chemokine (C-C motif) ligand 8	17
SUM190	CCL2	chemokine (C-C motif) ligand 2	17
SUM190	NLE1	notchless homolog 1 (Drosophila)	17
SUM190	RAD51L3	RAD51-like 3 (S. cerevisiae)	17
SUM190	CCL13	chemokine (C-C motif) ligand 13	17
SUM190	UNC45B	unc-45 homolog B (C. elegans)	17
SUM190	SLFN5	schlafen family member 5	17
SUM190	ACCN1	amiloride-sensitive cation channel 1, neuronal (degenerin)	17
SUM190	CCL1	chemokine (C-C motif) ligand 1	17
SUM190	CCL11	chemokine (C-C motif) ligand 11	17
SUM190	FNDC8	fibronectin type III domain containing 8	17
SUM190	AMAC1	acyl-malonyl condensing enzyme 1	17
SUM190	KLHL22	kelch-like 22 (Drosophila)	22
SUM190	ZNF74	zinc finger protein 74	22
SUM190	SNAP29	synaptosomal-associated protein, 29kDa	22
SUM190	MED15	mediator complex subunit 15	22
SUM190	PI4KA	phosphatidylinositol 4-kinase, catalytic, alpha	22
SUM190	LZTR1	leucine-zipper-like transcription regulator 1	22
SUM190	THAP7	THAP domain containing 7	22
SUM190	MGC16703	tubulin, alpha pseudogene	22
SUM190	CRKL	v-crk sarcoma virus CT10 oncogene homolog (avian)-like	22
SUM190	DGCR6L	DiGeorge syndrome critical region gene 6-like	22

SUM190	RTN4R	reticulon 4 receptor	22
SUM190	KIAA1666	KIAA1666 protein	22
SUM190	SERPIND1	serpin peptidase inhibitor, clade D (heparin cofactor), member 1	22
SUM190	SCARF2	scavenger receptor class F, member 2	22
SUM190	AIFM3	apoptosis-inducing factor, mitochondrion-associated, 3	22
SUM190	MPP1	membrane protein, palmitoylated 1, 55kDa	X
SUM190	VBP1	von Hippel-Lindau binding protein 1	X
SUM190	BRCC3	BRCA1/BRCA2-containing complex, subunit 3	X
SUM190	GAB3	GRB2-associated binding protein 3	X
SUM190	CLIC2	chloride intracellular channel 2	X
SUM190	F8A1	coagulation factor VIII-associated (intronic transcript) 1	X
SUM190	SPRY3	sprouty homolog 3 (Drosophila)	X
SUM190	RAB39B	RAB39B, member RAS oncogene family	X
SUM190	F8	coagulation factor VIII, procoagulant component (hemophilia A)	X
SUM190	MTCP1	mature T-cell proliferation 1	X
SUM190	H2AFB2	H2A histone family, member B2	X
SUM190	DKC1	dyskeratosis congenita 1, dyskerin	X
SUM190	FUNDC2	FUN14 domain containing 2	X
SUM190	TMLHE	trimethyllysine hydroxylase, epsilon	X
SUM190	PLXNB3	plexin B3	X
SUM190	ABCD1	ATP-binding cassette, sub-family D (ALD), member 1	X
SUM190	PNCK	pregnancy upregulated non-ubiquitously expressed CaM kinase	X
SUM190	SRPK3	SFRS protein kinase 3	X
SUM190	SSR4	signal sequence receptor, delta (translocon-associated protein delta)	X
SUM190	SLC6A8	solute carrier family 6 (neurotransmitter transporter, creatine), member 8	X
SUM190	IDH3G	isocitrate dehydrogenase 3 (NAD+) gamma	X
SUM190	L1CAM	L1 cell adhesion molecule	X
SUM190	MAGEA1	melanoma antigen family A, 1 (directs expression of antigen MZ2-E)	X
SUM190	PDZD4	PDZ domain containing 4	X
SUM190	FAM58A	family with sequence similarity 58, member A	X
SUM190	TREX2	three prime repair exonuclease 2	X
SUM190	ATP2B3	ATPase, Ca++ transporting, plasma membrane 3	X
SUM190	BCAP31	B-cell receptor-associated protein 31	X
SUM190	UCHL5IP	UCHL5 interacting protein	X
SUM190	BGN	biglycan	X
SUM190	DUSP9	dual specificity phosphatase 9	X

Supplementary Table S4: SUM-225 cell line copy number amplified genes.

Cell line	Symbol	Gene name	Chromosome
SUM225	CXCL5	chemokine (C-X-C motif) ligand 5	4
SUM225	VDP	vesicle docking protein p115	4
SUM225	ART3	ADP-ribosyltransferase 3	4
SUM225	SDAD1	SDA1 domain containing 1	4
SUM225	SCARB2	scavenger receptor class B, member 2	4
SUM225	C4orf26	chromosome 4 open reading frame 26	4
SUM225	BTC	betacellulin	4
SUM225	ANKRD17	ankyrin repeat domain 17	4
SUM225	G3BP2	GTPase activating protein (SH3 domain) binding protein 2	4
SUM225	COX18	COX18 cytochrome c oxidase assembly homolog (S. cerevisiae)	4
SUM225	NUP54	nucleoporin 54kDa	4
SUM225	RCHY1	ring finger and CHY zinc finger domain containing 1	4
SUM225	CXCL9	chemokine (C-X-C motif) ligand 9	4
SUM225	DKFZP564O0823	DKFZP564O0823 protein	4
SUM225	CXCL10	chemokine (C-X-C motif) ligand 10	4
SUM225	CXCL11	chemokine (C-X-C motif) ligand 11	4
SUM225	THAP6	THAP domain containing 6	4
SUM225	ALB	albumin	4
SUM225	AFP	alpha-fetoprotein	4
SUM225	CDKL2	cyclin-dependent kinase-like 2 (CDC2-related kinase)	4
SUM225	EPGN	epithelial mitogen homolog (mouse)	4
SUM225	CXCL6	chemokine (C-X-C motif) ligand 6 (granulocyte chemotactic protein 2)	4
SUM225	SHROOM3	shroom family member 3	4
SUM225	IL8	interleukin 8	4
SUM225	MTHFD2L	methylenetetrahydrofolate dehydrogenase (NADP+ dependent) 2-like	4
SUM225	EREG	epiregulin	4
SUM225	STBD1	starch binding domain 1	4
SUM225	CXCL3	chemokine (C-X-C motif) ligand 3	4
SUM225	PF4	platelet factor 4 (chemokine (C-X-C motif) ligand 4)	4
SUM225	ASAHL	N-acylsphingosine amidohydrolase (acid ceramidase)-like	4
SUM225	RASSF6	Ras association (RalGDS/AF-6) domain family 6	4
SUM225	CXCL1	chemokine (C-X-C motif) ligand 1 (melanoma growth stimulating activity, alpha)	4
SUM225	ADAMTS3	ADAM metallopeptidase with thrombospondin type 1 motif, 3	4
SUM225	AREG	amphiregulin (schwannoma-derived growth factor)	4

SUM225	CXCL2	chemokine (C-X-C motif) ligand 2	4
SUM225	11-Sep	septin 11	4
SUM225	AFM	afamin	4
SUM225	PF4V1	platelet factor 4 variant 1	4
SUM225	PPBP	pro-platelet basic protein (chemokine (C-X-C motif) ligand 7)	4
SUM225	PPEF2	protein phosphatase, EF-hand calcium binding domain 2	4
SUM225	CYP51A1	cytochrome P450, family 51, subfamily A, polypeptide 1	7
SUM225	CASD1	CAS1 domain containing 1	7
SUM225	CCDC132	coiled-coil domain containing 132	7
SUM225	FAM133B	family with sequence similarity 133, member B	7
SUM225	FZD1	frizzled homolog 1 (Drosophila)	7
SUM225	SAMD9L	sterile alpha motif domain containing 9-like	7
SUM225	ANKIB1	ankyrin repeat and IBR domain containing 1	7
SUM225	MTERF	mitochondrial transcription termination factor	7
SUM225	CDK6	cyclin-dependent kinase 6	7
SUM225	DKFZP564O0523	hypothetical protein DKFZp564O0523	7
SUM225	KRIT1	KRIT1, ankyrin repeat containing	7
SUM225	SAMD9	sterile alpha motif domain containing 9	7
SUM225	GATAD1	GATA zinc finger domain containing 1	7
SUM225	BET1	BET1 homolog (S. cerevisiae)	7
SUM225	PEX1	peroxisome biogenesis factor 1	7
SUM225	CALCR	calcitonin receptor	7
SUM225	SGCE	sarcoglycan, epsilon	7
SUM225	AKAP9	A kinase (PRKA) anchor protein (yotiao) 9	7
SUM225	GNG11	guanine nucleotide binding protein (G protein), gamma 11	7
SUM225	TFPI2	tissue factor pathway inhibitor 2	7
SUM225	GNGT1	guanine nucleotide binding protein (G protein), gamma transducing activity polypeptide 1	7
SUM225	COL1A2	collagen, type I, alpha 2	7
SUM225	LOC253012	hypothetical protein LOC253012	7
SUM225	PFTK1	PFTAIRE protein kinase 1	7
SUM225	PDK4	pyruvate dehydrogenase kinase, isozyme 4	7
SUM225	PON3	paraoxonase 3	7
SUM225	PON2	paraoxonase 2	7
SUM225	PON1	paraoxonase 1	7
SUM225	SLC25A13	solute carrier family 25, member 13 (citrin)	7
SUM225	PPP1R9A	protein phosphatase 1, regulatory (inhibitor) subunit 9A	7
SUM225	ASB4	ankyrin repeat and SOCS box-containing 4	7
SUM225	PEG10	paternally expressed 10	7
SUM225	DYNC1I1	dynein, cytoplasmic 1, intermediate chain 1	7
SUM225	RAB11FIP1	RAB11 family interacting protein 1 (class I)	8

SUM225	FKSG2	apoptosis inhibitor	8
SUM225	ERLIN2	ER lipid raft associated 2	8
SUM225	BRF2	BRF2, subunit of RNA polymerase III transcription initiation factor, BRF1-like	8
SUM225	PROSC	proline synthetase co-transcribed homolog (bacterial)	8
SUM225	ZNF703	zinc finger protein 703	8
SUM225	GPR124	G protein-coupled receptor 124	8
SUM225	GOT1L1	glutamic-oxaloacetic transaminase 1-like 1	8
SUM225	C8orf4	chromosome 8 open reading frame 4	8
SUM225	ZMAT4	zinc finger, matrin type 4	8
SUM225	INDO	indoleamine-pyrrole 2,3 dioxygenase	8
SUM225	ADAM2	ADAM metallopeptidase domain 2 (fertilin beta)	8
SUM225	EXOSC4	exosome component 4	8
SUM225	HSF1	heat shock transcription factor 1	8
SUM225	PUF60	poly-U binding splicing factor 60KDa	8
SUM225	CPSF1	cleavage and polyadenylation specific factor 1, 160kDa	8
SUM225	SLC39A4	solute carrier family 39 (zinc transporter), member 4	8
SUM225	KIAA1833	hypothetical protein KIAA1833	8
SUM225	ZNF16	zinc finger protein 16	8
SUM225	ZNF7	zinc finger protein 7	8
SUM225	COMMD5	COMM domain containing 5	8
SUM225	ZNF623	zinc finger protein 623	8
SUM225	ZNF707	zinc finger protein 707	8
SUM225	OPLAH	5-oxoprolinase (ATP-hydrolysing)	8
SUM225	C8orf30A	chromosome 8 open reading frame 30A	8
SUM225	ZNF34	zinc finger protein 34	8
SUM225	PYCRL	pyrroline-5-carboxylate reductase-like	8
SUM225	GPR172A	G protein-coupled receptor 172A	8
SUM225	CYHR1	cysteine/histidine-rich 1	8
SUM225	EPPK1	epiplakin 1	8
SUM225	FBXL6	F-box and leucine-rich repeat protein 6	8
SUM225	C8orf33	chromosome 8 open reading frame 33	8
SUM225	KIAA1688	KIAA1688 protein	8
SUM225	GLI4	GLI-Kruppel family member GLI4	8
SUM225	MAF1	MAF1 homolog (S. cerevisiae)	8
SUM225	LY6E	lymphocyte antigen 6 complex, locus E	8
SUM225	PPP1R16A	protein phosphatase 1, regulatory (inhibitor) subunit 16A	8
SUM225	EEF1D	eukaryotic translation elongation factor 1 delta (guanine nucleotide exchange protein)	8
SUM225	VPS28	vacuolar protein sorting 28 homolog (S. cerevisiae)	8
SUM225	ZNF696	zinc finger protein 696	8
SUM225	ADCK5	aarF domain containing kinase 5	8

SUM225	RPL8	ribosomal protein L8	8
SUM225	CYC1	cytochrome c-1	8
SUM225	RHPN1	rhophilin, Rho GTPase binding protein 1	8
SUM225	SCRIB	scribbled homolog (Drosophila)	8
SUM225	DGAT1	diacylglycerol O-acyltransferase homolog 1 (mouse)	8
SUM225	NRBP2	nuclear receptor binding protein 2	8
SUM225	ZNF250	zinc finger protein 250	8
SUM225	NFKBIL2	nuclear factor of kappa light polypeptide gene enhancer in B-cells inhibitor-like 2	8
SUM225	NAPRT1	nicotinate phosphoribosyltransferase domain containing 1	8
SUM225	GRINA	glutamate receptor, ionotropic, N-methyl D-asparate-associated protein 1 (glutamate binding)	8
SUM225	TSTA3	tissue specific transplantation antigen P35B	8
SUM225	LRRC24	leucine rich repeat containing 24	8
SUM225	ZFP41	zinc finger protein 41 homolog (mouse)	8
SUM225	KIFC2	kinesin family member C2	8
SUM225	GPAA1	glycosylphosphatidylinositol anchor attachment protein 1 homolog (yeast)	8
SUM225	RECQL4	RecQ protein-like 4	8
SUM225	PARP10	poly (ADP-ribose) polymerase family, member 10	8
SUM225	FAM83H	family with sequence similarity 83, member H	8
SUM225	TOP1MT	topoisomerase (DNA) I, mitochondrial	8
SUM225	ZC3H3	zinc finger CCCH-type containing 3	8
SUM225	CYP11B1	cytochrome P450, family 11, subfamily B, polypeptide 1	8
SUM225	LOC338328	high density lipoprotein-binding protein	8
SUM225	BOP1	block of proliferation 1	8
SUM225	LY6H	lymphocyte antigen 6 complex, locus H	8
SUM225	PLEC1	plectin 1, intermediate filament binding protein 500kDa	8
SUM225	TIGD5	tigger transposable element derived 5	8
SUM225	SCRT1	scratch homolog 1, zinc finger protein (Drosophila)	8
SUM225	GPT	glutamic-pyruvate transaminase (alanine aminotransferase)	8
SUM225	MFSD3	major facilitator superfamily domain containing 3	8
SUM225	C8orf31	chromosome 8 open reading frame 31	8
SUM225	MAPK15	mitogen-activated protein kinase 15	8
SUM225	MAFA	v-maf musculoaponeurotic fibrosarcoma oncogene homolog A (avian)	8
SUM225	FOXH1	forkhead box H1	8
SUM225	GSDMDC1	gasdermin domain containing 1	8
SUM225	ZNF251	zinc finger protein 251	8
SUM225	CYP11B2	cytochrome P450, family 11, subfamily B, polypeptide 2	8

SUM225	PXMP3	peroxisomal membrane protein 3, 35kDa (Zellweger syndrome)	8
SUM225	ZFHX4	zinc finger homeobox 4	8
SUM225	HNF4G	hepatocyte nuclear factor 4, gamma	8
SUM225	PKIA	protein kinase (cAMP-dependent, catalytic) inhibitor alpha	8
SUM225	WDR21C	WD repeat domain 21C	8
SUM225	TMEM65	transmembrane protein 65	8
SUM225	EIF2C2	eukaryotic translation initiation factor 2C, 2	8
SUM225	LYNX1	Ly6/neurotoxin 1	8
SUM225	TRMT12	tRNA methyltransferase 12 homolog (S. cerevisiae)	8
SUM225	SLC26A7	solute carrier family 26, member 7	8
SUM225	SQLE	squalene epoxidase	8
SUM225	JRK	jerky homolog (mouse)	8
SUM225	AZIN1	antizyme inhibitor 1	8
SUM225	SLC30A8	solute carrier family 30 (zinc transporter), member 8	8
SUM225	KHDRBS3	KH domain containing, RNA binding, signal transduction associated 3	8
SUM225	POLR2K	polymerase (RNA) II (DNA directed) polypeptide K, 7.0kDa	8
SUM225	ATP6V1C1	ATPase, H+ transporting, lysosomal 42kDa, V1 subunit C1	8
SUM225	RNF139	ring finger protein 139	8
SUM225	TTC35	tetratricopeptide repeat domain 35	8
SUM225	DERL1	Der1-like domain family, member 1	8
SUM225	EBAG9	estrogen receptor binding site associated, antigen, 9	8
SUM225	DPYS	dihydropyrimidinase	8
SUM225	WDSOF1	WD repeats and SOF1 domain containing	8
SUM225	SNTB1	syntrophin, beta 1 (dystrophin-associated protein A1, 59kDa, basic component 1)	8
SUM225	FBXO32	F-box protein 32	8
SUM225	FAM91A1	family with sequence similarity 91, member A1	8
SUM225	YWHAZ	tyrosine 3-monooxygenase/tryptophan 5-monooxygenase activation protein, zeta polypeptide	8
SUM225	ZNF572	zinc finger protein 572	8
SUM225	LY6D	lymphocyte antigen 6 complex, locus D	8
SUM225	RNF19A	ring finger protein 19A	8
SUM225	DEPDC6	DEP domain containing 6	8
SUM225	C8orf37	chromosome 8 open reading frame 37	8
SUM225	ZHX1	zinc fingers and homeoboxes 1	8
SUM225	SLC45A4	solute carrier family 45, member 4	8
SUM225	KIAA0143	KIAA0143 protein	8
SUM225	ADCY8	adenylate cyclase 8 (brain)	8

SUM225	HAS2	hyaluronan synthase 2	8
SUM225	C8orf53	chromosome 8 open reading frame 53	8
SUM225	ANGPT1	angiopoietin 1	8
SUM225	TRIB1	tribbles homolog 1 (Drosophila)	8
SUM225	NSMCE2	non-SMC element 2, MMS21 homolog (S. cerevisiae)	8
SUM225	TMEM67	transmembrane protein 67	8
SUM225	TSPYL5	TSPY-like 5	8
SUM225	INTS8	integrator complex subunit 8	8
SUM225	RAD21	RAD21 homolog (S. pombe)	8
SUM225	ZNF706	zinc finger protein 706	8
SUM225	C8orf76	chromosome 8 open reading frame 76	8
SUM225	ANXA13	annexin A13	8
SUM225	RIMS2	regulating synaptic membrane exocytosis 2	8
SUM225	STK3	serine/threonine kinase 3 (STE20 homolog, yeast)	8
SUM225	FAM49B	family with sequence similarity 49, member B	8
SUM225	PTP4A3	protein tyrosine phosphatase type IVA, member 3	8
SUM225	KLF10	Kruppel-like factor 10	8
SUM225	PABPC1	poly(A) binding protein, cytoplasmic 1	8
SUM225	PHF20L1	PHD finger protein 20-like 1	8
SUM225	ZFAT1	ZFAT zinc finger 1	8
SUM225	PTK2	PTK2 protein tyrosine kinase 2	8
SUM225	TSNARE1	t-SNARE domain containing 1	8
SUM225	TAF2	TAF2 RNA polymerase II, TATA box binding protein (TBP)-associated factor, 150kDa	8
SUM225	WWP1	WW domain containing E3 ubiquitin protein ligase 1	8
SUM225	RPL30	ribosomal protein L30	8
SUM225	ARC	activity-regulated cytoskeleton-associated protein	8
SUM225	NDRG1	N-myc downstream regulated gene 1	8
SUM225	UBR5	ubiquitin protein ligase E3 component n-recognin 5	8
SUM225	PPM2C	protein phosphatase 2C, magnesium-dependent, catalytic subunit	8
SUM225	OSR2	odd-skipped related 2 (Drosophila)	8
SUM225	OTUD6B	OTU domain containing 6B	8
SUM225	NIBP	NIK and IKK{beta} binding protein	8
SUM225	NPAL2	NIPA-like domain containing 2	8
SUM225	TNFRSF11B	tumor necrosis factor receptor superfamily, member 11b (osteoprotegerin)	8
SUM225	RUNX1T1	runt-related transcription factor 1; translocated to, 1 (cyclin D-related)	8
SUM225	CHRAC1	chromatin accessibility complex 1	8
SUM225	COL22A1	collagen, type XXII, alpha 1	8
SUM225	MRPL13	mitochondrial ribosomal protein L13	8
SUM225	NDUFB9	NADH dehydrogenase (ubiquinone) 1 beta subcomplex, 9, 22kDa	8

SUM225	LOC441376	AARD protein	8
SUM225	FAM84B	family with sequence similarity 84, member B	8
SUM225	KIAA1429	KIAA1429	8
SUM225	DDEF1	development and differentiation enhancing factor 1	8
SUM225	GDF6	growth differentiation factor 6	8
SUM225	ANKRD46	ankyrin repeat domain 46	8
SUM225	MTERFD1	MTERF domain containing 1	8
SUM225	KCNQ3	potassium voltage-gated channel, KQT-like subfamily, member 3	8
SUM225	PGCP (2X)	plasma glutamate carboxypeptidase	8
SUM225	RIPK2	receptor-interacting serine-threonine kinase 2	8
SUM225	RBM12B	RNA binding motif protein 12B	8
SUM225	EIF3E	eukaryotic translation initiation factor 3, subunit E	8
SUM225	LAPTM4B	lysosomal associated protein transmembrane 4 beta	8
SUM225	MLZE	melanoma-derived leucine zipper, extra-nuclear factor	8
SUM225	UQCRB	ubiquinol-cytochrome c reductase binding protein	8
SUM225	EIF3H	eukaryotic translation initiation factor 3, subunit H	8
SUM225	RGS22	regulator of G-protein signaling 22	8
SUM225	C8orf47	chromosome 8 open reading frame 47	8
SUM225	FLJ43860	FLJ43860 protein	8
SUM225	CCNE2	cyclin E2	8
SUM225	SPAG1	sperm associated antigen 1	8
SUM225	C8orf55	chromosome 8 open reading frame 55	8
SUM225	MMP16	matrix metallopeptidase 16 (membrane-inserted)	8
SUM225	LY6K	lymphocyte antigen 6 complex, locus K	8
SUM225	PLEKHF2	pleckstrin homology domain containing, family F (with FYVE domain) member 2	8
SUM225	ATAD2	ATPase family, AAA domain containing 2	8
SUM225	EFCBP1	EF-hand calcium binding protein 1	8
SUM225	TG	thyroglobulin	8
SUM225	SLA	Src-like-adaptor	8
SUM225	DENND3	DENN/MADD domain containing 3	8
SUM225	PSCA	prostate stem cell antigen	8
SUM225	KIAA0196	KIAA0196	8
SUM225	NUDCD1	NudC domain containing 1	8
SUM225	COL14A1	collagen, type XIV, alpha 1 (undulin)	8
SUM225	FZD6	frizzled homolog 6 (Drosophila)	8
SUM225	SLC7A13	solute carrier family 7, (cationic amino acid transporter, y+ system) member 13	8
SUM225	SLC25A32	solute carrier family 25, member 32	8
SUM225	ST3GAL1	ST3 beta-galactoside alpha-2,3-sialyltransferase 1	8
SUM225	OXR1	oxidation resistance 1	8

SUM225	TMEM55A	transmembrane protein 55A	8
SUM225	NBN	nibrin	8
SUM225	CPNE3	copine III	8
SUM225	SDC2	syndecan 2	8
SUM225	TMEM71	transmembrane protein 71	8
SUM225	C8orf38	chromosome 8 open reading frame 38	8
SUM225	ATP6V0D2	ATPase, H+ transporting, lysosomal 38kDa, V0 subunit d2	8
SUM225	GRHL2	grainyhead-like 2 (Drosophila)	8
SUM225	C8orf32	chromosome 8 open reading frame 32	8
SUM225	ZFPM2	zinc finger protein, multitype 2	8
SUM225	TP53INP1	tumor protein p53 inducible nuclear protein 1	8
SUM225	SLURP1	secreted LY6/PLAUR domain containing 1	8
SUM225	ZHX2	zinc fingers and homeoboxes 2	8
SUM225	BAALC	brain and acute leukemia, cytoplasmic	8
SUM225	DPY19L4	dpy-19-like 4 (C. elegans)	8
SUM225	COX6C	cytochrome c oxidase subunit VIc	8
SUM225	WDR67	WD repeat domain 67	8
SUM225	KCNK9	potassium channel, subfamily K, member 9	8
SUM225	WISP1	WNT1 inducible signaling pathway protein 1	8
SUM225	GOLSYN	Golgi-localized protein	8
SUM225	HRSP12	heat-responsive protein 12	8
SUM225	PTDSS1	phosphatidylserine synthase 1	8
SUM225	MGC39715	hypothetical protein MGC39715	8
SUM225	VPS13B	vacuolar protein sorting 13 homolog B (yeast)	8
SUM225	EXT1	exostoses (multiple) 1	8
SUM225	MTBP	Mdm2, transformed 3T3 cell double minute 2, p53 binding protein (mouse) binding protein, 104kDa	8
SUM225	OSGIN2	oxidative stress induced growth inhibitor family member 2	8
SUM225	MAL2	mal, T-cell differentiation protein 2	8
SUM225	RAD54B	RAD54 homolog B (S. cerevisiae)	8
SUM225	NOV	nephroblastoma overexpressed gene	8
SUM225	MTSS1	metastasis suppressor 1	8
SUM225	CTHRC1	collagen triple helix repeat containing 1	8
SUM225	GPR20	G protein-coupled receptor 20	8
SUM225	CSMD3	CUB and Sushi multiple domains 3	8
SUM225	TM7SF4	transmembrane 7 superfamily member 4	8
SUM225	MATN2	matrilin 2	8
SUM225	ENY2	enhancer of yellow 2 homolog (Drosophila)	8
SUM225	GML	GPI anchored molecule like protein	8
SUM225	FAM83A	family with sequence similarity 83, member A	8
SUM225	ENPP2	ectonucleotide pyrophosphatase/ phosphodiesterase 2 (autotaxin)	8
SUM225	PKHD1L1	polycystic kidney and hepatic disease 1 (autosomal recessive)-like 1	8

SUM225	COLEC10	collectin sub-family member 10 (C-type lectin)	8
SUM225	RBM35A	RNA binding motif protein 35A	8
SUM225	LRRC6	leucine rich repeat containing 6	8
SUM225	DECR1	2,4-dienoyl CoA reductase 1, mitochondrial	8
SUM225	MYC	v-myc myelocytomatosis viral oncogene homolog (avian)	8
SUM225	LYPD2	LY6/PLAUR domain containing 2	8
SUM225	FAM82B	family with sequence similarity 82, member B	8
SUM225	TATDN1	TatD DNase domain containing 1	8
SUM225	NCALD	neurocalcin delta	8
SUM225	GEM	GTP binding protein overexpressed in skeletal muscle	8
SUM225	TMEM64	transmembrane protein 64	8
SUM225	CALB1	calbindin 1, 28kDa	8
SUM225	TRPS1	trichorhinophalangeal syndrome I	8
SUM225	RSPO2	R-spondin 2 homolog (Xenopus laevis)	8
SUM225	DCC1	defective in sister chromatid cohesion homolog 1 (S. cerevisiae)	8
SUM225	TMEM74	transmembrane protein 74	8
SUM225	MED30	mediator complex subunit 30	8
SUM225	POP1	processing of precursor 1, ribonuclease P/MRP subunit (S. cerevisiae)	8
SUM225	LRP12	low density lipoprotein-related protein 12	8
SUM225	MTDH	metadherin	8
SUM225	RRM2B	ribonucleotide reductase M2 B (TP53 inducible)	8
SUM225	BAI1	brain-specific angiogenesis inhibitor 1	8
SUM225	PSKH2	protein serine kinase H2	8
SUM225	CNGB3	cyclic nucleotide gated channel beta 3	8
SUM225	CNBD1	cyclic nucleotide binding domain containing 1	8
SUM225	CDH17	cadherin 17, LI cadherin (liver-intestine)	8
SUM225	KCNS2	potassium voltage-gated channel, delayed-rectifier, subfamily S, member 2	8
SUM225	ODF1	outer dense fiber of sperm tails 1	8
SUM225	ABRA	actin-binding Rho activating protein	8
SUM225	TRHR	thyrotropin-releasing hormone receptor	8
SUM225	KCNV1	potassium channel, subfamily V, member 1	8
SUM225	FAM135B	family with sequence similarity 135, member B	8
SUM225	YARS2	tyrosyl-tRNA synthetase 2, mitochondrial	12
SUM225	DNM1L	dynamin 1-like	12
SUM225	SYT10	synaptotagmin X	12
SUM225	ALG10	asparagine-linked glycosylation 10 homolog (yeast, alpha-1,2-glucosyltransferase)	12
SUM225	FGD4	FYVE, RhoGEF and PH domain containing 4	12
SUM225	PKP2	plakophilin 2	12
SUM225	BICD1	bicaudal D homolog 1 (Drosophila)	12
SUM225	ERGIC2	ERGIC and golgi 2	12

SUM225	IPO8	importin 8	12
SUM225	MLSTD1	male sterility domain containing 1	12
SUM225	TMTC1	transmembrane and tetratricopeptide repeat containing 1	12
SUM225	CCDC91	coiled-coil domain containing 91	12
SUM225	CX3CL1	chemokine (C-X3-C motif) ligand 1	16
SUM225	POLR2C	polymerase (RNA) II (DNA directed) polypeptide C, 33kDa	16
SUM225	DOK4	docking protein 4	16
SUM225	NUDT21	nudix (nucleoside diphosphate linked moiety X)-type motif 21	16
SUM225	BRD7	bromodomain containing 7	16
SUM225	NDRG4	NDRG family member 4	16
SUM225	COQ9	coenzyme Q9 homolog (S. cerevisiae)	16
SUM225	NIP30	NEFA-interacting nuclear protein NIP30	16
SUM225	MMP15	matrix metallopeptidase 15 (membrane-inserted)	16
SUM225	HEATR3	HEAT repeat containing 3	16
SUM225	N4BP1	Nedd4 binding protein 1	16
SUM225	IRX6	iroquois homeobox 6	16
SUM225	NKD1	naked cuticle homolog 1 (Drosophila)	16
SUM225	BBS2	Bardet-Biedl syndrome 2	16
SUM225	PAPD5	PAP associated domain containing 5	16
SUM225	ITFG1	integrin alpha FG-GAP repeat containing 1	16
SUM225	C16orf80	chromosome 16 open reading frame 80	16
SUM225	CNOT1	CCR4-NOT transcription complex, subunit 1	16
SUM225	PHKB	phosphorylase kinase, beta	16
SUM225	IRX3	iroquois homeobox 3	16
SUM225	CBLN1	cerebellin 1 precursor	16
SUM225	DNAJA2	DnaJ (Hsp40) homolog, subfamily A, member 2	16
SUM225	LONP2	lon peptidase 2, peroxisomal	16
SUM225	CYLD	cylindromatosis (turban tumor syndrome)	16
SUM225	FLJ10815	amino acid transporter	16
SUM225	CIAPIN1	cytokine induced apoptosis inhibitor 1	16
SUM225	SIAH1	seven in absentia homolog 1 (Drosophila)	16
SUM225	OGFOD1	2-oxoglutarate and iron-dependent oxygenase domain containing 1	16
SUM225	NLRC5	NLR family, CARD domain containing 5	16
SUM225	ZNF319	zinc finger protein 319	16
SUM225	NUP93	nucleoporin 93kDa	16
SUM225	CCDC102A	coiled-coil domain containing 102A	16
SUM225	CHD9	chromodomain helicase DNA binding protein 9	16
SUM225	GOT2	glutamic-oxaloacetic transaminase 2, mitochondrial (aspartate aminotransferase 2)	16
SUM225	TMEM188	transmembrane protein 188	16
SUM225	CCL17	chemokine (C-C motif) ligand 17	16
SUM225	GPR56	G protein-coupled receptor 56	16
SUM225	HERPUD1	homocysteine-inducible, endoplasmic reticulum stress-inducible, ubiquitin-like domain member 1	16

SUM225	C16orf57	chromosome 16 open reading frame 57	16
SUM225	CES7	carboxylesterase 7	16
SUM225	GPT2	glutamic pyruvate transaminase (alanine aminotransferase) 2	16
SUM225	FTO	fat mass and obesity associated	16
SUM225	GINS3	GINS complex subunit 3 (Psf3 homolog)	16
SUM225	KATNB1	katanin p80 (WD repeat containing) subunit B 1	16
SUM225	LOC388272	similar to RIKEN cDNA 4921524J17	16
SUM225	ABCC11	ATP-binding cassette, sub-family C (CFTR/MRP), member 11	16
SUM225	AKTIP	AKT interacting protein	16
SUM225	NOD2	nucleotide-binding oligomerization domain containing 2	16
SUM225	IRX5	iroquois homeobox 5	16
SUM225	RBL2	retinoblastoma-like 2 (p130)	16
SUM225	MT1F	metallothionein 1F	16
SUM225	SALL1	sal-like 1 (Drosophila)	16
SUM225	CSNK2A2	casein kinase 2, alpha prime polypeptide	16
SUM225	MT1M	metallothionein 1M	16
SUM225	CPNE2	copine II	16
SUM225	AMFR	autocrine motility factor receptor	16
SUM225	RPGRIP1L	RPGRIP1-like	16
SUM225	CNGB1	cyclic nucleotide gated channel beta 1	16
SUM225	AYTL1	acyltransferase like 1	16
SUM225	CCDC113	coiled-coil domain containing 113	16
SUM225	GPR114	G protein-coupled receptor 114	16
SUM225	KIFC3	kinesin family member C3	16
SUM225	MMP2	matrix metallopeptidase 2 (gelatinase A, 72kDa gelatinase, 72kDa type IV collagenase)	16
SUM225	NETO2	neuropilin (NRP) and tolloid (TLL)-like 2	16
SUM225	SETD6	SET domain containing 6	16
SUM225	CAPNS2	calpain, small subunit 2	16
SUM225	MT3	metallothionein 3	16
SUM225	CES1	carboxylesterase 1 (monocyte/macrophage serine esterase 1)	16
SUM225	TEPP	testis/prostate/placenta-expressed protein	16
SUM225	ARL2BP	ADP-ribosylation factor-like 2 binding protein	16
SUM225	ZNF423	zinc finger protein 423	16
SUM225	MT2A	metallothionein 2A	16
SUM225	GPR97	G protein-coupled receptor 97	16
SUM225	MT1X	metallothionein 1X	16
SUM225	MT1H	metallothionein 1H	16
SUM225	ADCY7	adenylate cyclase 7	16
SUM225	MT1A	metallothionein 1A	16
SUM225	MT1G	metallothionein 1G	16
SUM225	CETP	cholesteryl ester transfer protein, plasma	16

SUM225	MT1B	metallothionein 1B	16
SUM225	CCDC135	coiled-coil domain containing 135	16
SUM225	SLC12A3	solute carrier family 12 (sodium/chloride transporters), member 3	16
SUM225	GNAO1	guanine nucleotide binding protein (G protein), alpha activating activity polypeptide O	16
SUM225	MT1E	metallothionein 1E	16
SUM225	C16orf78	chromosome 16 open reading frame 78	16
SUM225	PLLP	plasma membrane proteolipid (plasmolipin)	16
SUM225	ABCC12	ATP-binding cassette, sub-family C (CFTR/MRP), member 12	16
SUM225	SLIC1	selectin ligand interactor cytoplasmic-1	16
SUM225	SLC6A2	solute carrier family 6 (neurotransmitter transporter, noradrenalin), member 2	16
SUM225	MT4	metallothionein 4	16
SUM225	CCL22	chemokine (C-C motif) ligand 22	16
SUM225	Klkbl4	plasma kallikrein-like protein 4	16
SUM225	CDH8	cadherin 8, type 2	16
SUM225	C16orf70	chromosome 16 open reading frame 70	16
SUM225	CENPT	centromere protein T	16
SUM225	PSKH1	protein serine kinase H1	16
SUM225	HSPC171	HSPC171 protein	16
SUM225	WWP2	WW domain containing E3 ubiquitin protein ligase 2	16
SUM225	HSF4	heat shock transcription factor 4	16
SUM225	DYNC1LI2	dynein, cytoplasmic 1, light intermediate chain 2	16
SUM225	GFOD2	glucose-fructose oxidoreductase domain containing 2	16
SUM225	FHOD1	formin homology 2 domain containing 1	16
SUM225	DPEP3	dipeptidase 3	16
SUM225	SMPD3	sphingomyelin phosphodiesterase 3, neutral membrane (neutral sphingomyelinase II)	16
SUM225	LOC653319	hypothetical protein LOC653319	16
SUM225	CMTM2	CKLF-like MARVEL transmembrane domain containing 2	16
SUM225	ATP6V0D1	ATPase, H+ transporting, lysosomal 38kDa, V0 subunit d1	16
SUM225	LRRC36	leucine rich repeat containing 36	16
SUM225	RANBP10	RAN binding protein 10	16
SUM225	NOL3	nucleolar protein 3 (apoptosis repressor with CARD domain)	16
SUM225	NQO1	NAD(P)H dehydrogenase, quinone 1	16
SUM225	SLC9A5	solute carrier family 9 (sodium/hydrogen exchanger), member 5	16
SUM225	SLC7A6	solute carrier family 7 (cationic amino acid transporter, y+ system), member 6	16
SUM225	SNTB2	syntrophin, beta 2 (dystrophin-associated protein A1, 59kDa, basic component 2)	16

SUM225	GOT2	glutamic-oxaloacetic transaminase 2, mitochondrial (aspartate aminotransferase 2)	16
SUM225	PLEKHG4	pleckstrin homology domain containing, family G (with RhoGef domain) member 4	16
SUM225	CYB5B	cytochrome b5 type B (outer mitochondrial membrane)	16
SUM225	HSD11B2	hydroxysteroid (11-beta) dehydrogenase 2	16
SUM225	VPS4A	vacuolar protein sorting 4 homolog A (S. cerevisiae)	16
SUM225	CTCF	CCCTC-binding factor (zinc finger protein)	16
SUM225	NFATC3	nuclear factor of activated T-cells, cytoplasmic, calcineurin-dependent 3	16
SUM225	CMTM4	CKLF-like MARVEL transmembrane domain containing 4	16
SUM225	LONP2	core-binding factor, beta subunit	16
SUM225	NFAT5	nuclear factor of activated T-cells 5, tonicity-responsive	16
SUM225	PSMB10	proteasome (prosome, macropain) subunit, beta type, 10	16
SUM225	RBM35B	RNA binding motif protein 35B	16
SUM225	DDX28	DEAD (Asp-Glu-Ala-Asp) box polypeptide 28	16
SUM225	CDH3	cadherin 3, type 1, P-cadherin (placental)	16
SUM225	APPBP1	amyloid beta precursor protein binding protein 1	16
SUM225	CDH1	cadherin 1, type 1, E-cadherin (epithelial)	16
SUM225	COG8	component of oligomeric golgi complex 8	16
SUM225	CES2	carboxylesterase 2 (intestine, liver)	16
SUM225	AGRP	agouti related protein homolog (mouse)	16
SUM225	CES3	carboxylesterase 3 (brain)	16
SUM225	TERF2	telomeric repeat binding factor 2	16
SUM225	RLTPR	RGD, leucine-rich repeat, tropomodulin and proline-rich containing protein	16
SUM225	RRAD	Ras-related associated with diabetes	16
SUM225	PDP2	pyruvate dehydrogenase phosphatase isoenzyme 2	16
SUM225	MGC4655	hypothetical protein MGC4655	16
SUM225	E2F4	E2F transcription factor 4, p107/p130-binding	16
SUM225	THAP11	THAP domain containing 11	16
SUM225	CKLF	chemokine-like factor	16
SUM225	CMTM3	CKLF-like MARVEL transmembrane domain containing 3	16
SUM225	TK2	thymidine kinase 2, mitochondrial	16
SUM225	CDH5	cadherin 5, type 2, VE-cadherin (vascular epithelium)	16
SUM225	EDC4	enhancer of mRNA decapping 4	16
SUM225	CIRH1A	cirrhosis, autosomal recessive 1A (cirhin)	16
SUM225	LYPLA3	lysophospholipase 3 (lysosomal phospholipase A2)	16
SUM225	FAM96B	family with sequence similarity 96, member B	16
SUM225	C16orf48	chromosome 16 open reading frame 48	16

SUM225	TMCO7	transmembrane and coiled-coil domains 7	16
SUM225	DUS2L	dihydrouridine synthase 2-like, SMM1 homolog (S. cerevisiae)	16
SUM225	CDH11	cadherin 11, type 2, OB-cadherin (osteoblast)	16
SUM225	NIP7	nuclear import 7 homolog (S. cerevisiae)	16
SUM225	LOC388284	hypothetical gene supported by BC032064; BC041612	16
SUM225	PDPR	pyruvate dehydrogenase phosphatase regulatory subunit	16
SUM225	NUTF2	nuclear transport factor 2	16
SUM225	TSNAXIP1	translin-associated factor X interacting protein 1	16
SUM225	CDH16	cadherin 16, KSP-cadherin	16
SUM225	FLJ37464	hypothetical protein FLJ37464	16
SUM225	CTF8	chromosome transmission fidelity factor 8 homolog (S. cerevisiae)	16
SUM225	HAS3	hyaluronan synthase 3	16
SUM225	TMED6	transmembrane emp24 protein transport domain containing 6	16
SUM225	PARD6A	par-6 partitioning defective 6 homolog alpha (C. elegans)	16
SUM225	LCAT	lecithin-cholesterol acyltransferase	16
SUM225	NRN1L	neuritin 1-like	16
SUM225	NOB1	NIN1/RPN12 binding protein 1 homolog (S. cerevisiae)	16
SUM225	CTRL	chymotrypsin-like	16
SUM225	ZDHHC1	zinc finger, DHHC-type containing 1	16
SUM225	ELMO3	engulfment and cell motility 3	16
SUM225	DPEP2	dipeptidase 2	16
SUM225	SLC12A4	solute carrier family 12 (potassium/chloride transporters), member 4	16
SUM225	ACD	adrenocortical dysplasia homolog (mouse)	16
SUM225	EXOC3L	exocyst complex component 3-like	16
SUM225	FAM65A	family with sequence similarity 65, member A	16
SUM225	TPPP3	tubulin polymerization-promoting protein family member 3	16
SUM225	CDH8	cadherin 8, type 2	16
SUM225	CA7	carbonic anhydrase VII	16
SUM225	LRRC29	leucine rich repeat containing 29	16
SUM225	VPS35	vacuolar protein sorting 35 homolog (S. cerevisiae)	16
SUM225	LOC388272	similar to RIKEN cDNA 4921524J17	16
SUM225	ORC6L	origin recognition complex, subunit 6 like (yeast)	16
SUM225	MLCK	MLCK protein	16
SUM225	SHCBP1	SHC SH2-domain binding protein 1	16
SUM225	STARD3	StAR-related lipid transfer (START) domain containing 3	17
SUM225	NEUROD2	neurogenic differentiation 2	17
SUM225	TCAP	titin-cap (telethonin)	17

SUM225	C17orf37	chromosome 17 open reading frame 37	17
SUM225	ERBB2	v-erb-b2 erythroblastic leukemia viral oncogene homolog 2, neuro/glioblastoma derived oncogene homolog (avian)	17
SUM225	PNMT	phenylethanolamine N-methyltransferase	17
SUM225	PERLD1	per1-like domain containing 1	17
SUM225	GRB7	growth factor receptor-bound protein 7	17
SUM225	IKZF3	IKAROS family zinc finger 3 (Aiolos)	17
SUM225	PPP1R1B	protein phosphatase 1, regulatory (inhibitor) subunit 1B (dopamine and cAMP regulated phosphoprotein, DARPP-32)	17
SUM225	CRKRS	Cdc2-related kinase, arginine/serine-rich	17
SUM225	ZPBP2	zona pellucida binding protein 2	17
SUM225	SLC35B1	solute carrier family 35, member B1	17
SUM225	MYST2	MYST histone acetyltransferase 2	17
SUM225	SPOP	speckle-type POZ protein	17
SUM225	FAM117A	family with sequence similarity 117, member A	17
SUM225	PHB	prohibitin	17
SUM225	NGFR	nerve growth factor receptor (TNFR superfamily, member 16)	17
SUM225	ZNF652	zinc finger protein 652	17
SUM225	NXPH3	neurexophilin 3	17
SUM225	PHOSPHO1	phosphatase, orphan 1	17
SUM225	ALG12	asparagine-linked glycosylation 12 homolog (S. cerevisiae, alpha-1,6-mannosyltransferase)	22
SUM225	CRELD2	cysteine-rich with EGF-like domains 2	22
SUM225	ZBED4	zinc finger, BED-type containing 4	22
SUM225	TRABD	TraB domain containing	22
SUM225	MOV10L1	Mov10l1, Moloney leukemia virus 10-like 1, homolog (mouse)	22
SUM225	PIM3	pim-3 oncogene	22
SUM225	PANX2	pannexin 2	22
SUM225	BRD1	bromodomain containing 1	22
SUM225	FAM19A5	family with sequence similarity 19 (chemokine (C-C motif)-like), member A5	22
SUM225	MLC1	megalencephalic leukoencephalopathy with subcortical cysts 1	22
SUM225	EIF4ENIF1	eukaryotic translation initiation factor 4E nuclear import factor 1	22
SUM225	MORC2	MORC family CW-type zinc finger 2	22
SUM225	RNF185	ring finger protein 185	22
SUM225	YWHAH	tyrosine 3-monooxygenase/tryptophan 5-monooxygenase activation protein, eta polypeptide	22
SUM225	PIK3IP1	phosphoinositide-3-kinase interacting protein 1	22
SUM225	SFI1	Sfi1 homolog, spindle assembly associated (yeast)	22
SUM225	C22orf30	chromosome 22 open reading frame 30	22
SUM225	LIMK2	LIM domain kinase 2	22
SUM225	DEPDC5	DEP domain containing 5	22

SUM225	SLC5A1	solute carrier family 5 (sodium/glucose cotransporter), member 1	22
SUM225	SMTN	smoothelin	22
SUM225	PATZ1	POZ (BTB) and AT hook containing zinc finger 1	22
SUM225	SELM	selenoprotein M	22
SUM225	DRG1	developmentally regulated GTP binding protein 1	22
SUM225	PISD	phosphatidylserine decarboxylase	22
SUM225	PLA2G3	phospholipase A2, group III	22
SUM225	C22orf24	chromosome 22 open reading frame 24	22
SUM225	OSBP2	oxysterol binding protein 2	22

Supplementary Table S5: SUM-52 cell line shRNA screen results

Supplementary Table S6: SUM-185 cell line shRNA screen results

Supplementary Table S7: SUM-190 cell line shRNA screen results

Supplementary Table S8: SUM-225 cell line shRNA screen results
